# Automated Mini-Column Solid-Phase Extraction Cleanup for High-Throughput Analysis of Chemical Contaminants in Foods by Low-Pressure Gas Chromatography—Tandem Mass Spectrometry

**DOI:** 10.1007/s10337-016-3116-y

**Published:** 2016-06-13

**Authors:** Steven J. Lehotay, Lijun Han, Yelena Sapozhnikova

**Affiliations:** 1 US Department of Agriculture, Agricultural Research Service, Eastern Regional Research Center, 600 East Mermaid Lane, Wyndmoor, PA 19038 USA; 2 College of Science, China Agricultural University, Beijing, 100193 China

**Keywords:** High-throughput automation, Solid-phase extraction cleanup, Pesticide residue analysis, QuEChERS sample preparation, Fast GC-MS/MS, Analyte protectants, Environmental contaminants, Foods

## Abstract

This study demonstrated the application of an automated high-throughput mini-cartridge solid-phase extraction (mini-SPE) cleanup for the rapid low-pressure gas chromatography—tandem mass spectrometry (LPGC-MS/MS) analysis of pesticides and environmental contaminants in QuEChERS extracts of foods. Cleanup efficiencies and breakthrough volumes using different mini-SPE sorbents were compared using avocado, salmon, pork loin, and kale as representative matrices. Optimum extract load volume was 300 µL for the 45 mg mini-cartridges containing 20/12/12/1 (w/w/w/w) anh. MgSO_4_/PSA (primary secondary amine)/C_18_/CarbonX sorbents used in the final method. In method validation to demonstrate high-throughput capabilities and performance results, 230 spiked extracts of 10 different foods (apple, kiwi, carrot, kale, orange, black olive, wheat grain, dried basil, pork, and salmon) underwent automated mini-SPE cleanup and analysis over the course of 5 days. In all, 325 analyses for 54 pesticides and 43 environmental contaminants (3 analyzed together) were conducted using the 10 min LPGC-MS/MS method without changing the liner or retuning the instrument. Merely, 1 mg equivalent sample injected achieved <5 ng g^−1^ limits of quantification. With the use of internal standards, method validation results showed that 91 of the 94 analytes including pairs achieved satisfactory results (70–120 % recovery and RSD ≤ 25 %) in the 10 tested food matrices (*n* = 160). Matrix effects were typically less than ±20 %, mainly due to the use of analyte protectants, and minimal human review of software data processing was needed due to summation function integration of analyte peaks. This study demonstrated that the automated mini-SPE + LPGC-MS/MS method yielded accurate results in rugged, high-throughput operations with minimal labor and data review.

**Electronic supplementary material:**

The online version of this article (doi:10.1007/s10337-016-3116-y) contains supplementary material, which is available to authorized users.

## Introduction

Trade of food products continues to increase globally [1], which is leading to greater food safety concerns [2, 3], and recent legislation [4] places greater emphasis on a higher rate of monitoring by private as well as regulatory laboratories to test for pesticide residues and other contaminants in the commodities. However, the cost of monitoring adds to the price of the food to the consumer, and delays in the analysis of perishable items reduces shelf life and sales of the product. Yet, more pesticides are being registered monthly for different crops worldwide [5], while human health and ecotoxicological risk assessment studies lead to frequent modifications of maximum residue limits (MRLs) [6], which places great demands on labs and methods to achieve high quality results, including analyte identification [7] of an ever expanding scope of ultra-trace contaminants in diverse, complicated food matrices. High economic, legal, and health risks are at stake, and the goal of the routine monitoring lab is to provide accurate results in the most efficient (cost-effective, high-throughput, automated) process possible.

Streamlined sample preparation with approaches such as QuEChERS (quick, easy, cheap, effective, rugged, and safe) [8, 9], and expanding scope of analytes to include environmental and emerging contaminants as well as pesticides [10], provides value in both higher sample throughput and savings by performing fewer methods for the same samples. Automation of sample cleanup conducted in parallel with the analytical step typically decreases labor, improves precision, and yields higher sample throughput. As a result, a number of different robotic instruments and approaches are available for automated sample preparation in food analysis applications [11].

Morris and Schriner [12] described an automated mini-cartridge solid-phase extraction (mini-SPE) cleanup approach called “instrument top sample preparation” (ITSP). They developed and demonstrated automated mini-SPE of QuEChERS extracts for liquid chromatography–tandem mass spectrometry (LC–MS/MS) analysis of 263 pesticide analytes in avocado, citrus and buttercup squash [12]. Cartridge-based SPE (c-SPE) usually provides better cleanup than dispersive-SPE (d-SPE) that is often incorporated in QuEChERS sample preparation [9]. In manual batch applications, d-SPE is usually cheaper, easier, and faster than traditional c-SPE, especially filter-vial d-SPE which conveniently cleans and filters extracts in autosampler vials [13]. However, online SPE techniques that perform cleanup at the same time as the analytical step have the added potential to reduce analyte degradation possible in batch operations when many hours can pass before final extracts are analyzed in long sequences.

Ultrahigh-performance (UHP) LC–MS/MS usually takes about 10–15 min for analysis of hundreds of analytes [14, 15], but conventional gas chromatography (GC)–MS/(MS) commonly entails 25–50 min per sample [16]. However, low-pressure (LP) GC-MS(/MS) only takes 10 min per analysis of the same number of analytes with an acceptably small loss of separation efficiency, while providing increased sample capacity, sensitivity, and ruggedness [17]. In high-throughput analyses involving both LC- and GC-amenable analytes, the sample throughput is limited by the slower method, and LPGC-MS/MS is fast enough to keep pace with UHPLC-MS/MS [18].

The aim of this study was to evaluate, optimize, and validate automated mini-SPE cleanup of QuEChERS extracts, and demonstrate the feasibility of the robotic sample preparation coupled with fast LPGC-MS/MS using analyte protectants and summation function integration to provide high-throughput monitoring of pesticides and other residues in foods with minimal human involvement. A state-of-the-art GC-MS/MS was used to reduce the amount of equivalent sample injected and extend maintenance-free operation of the instrument while still meeting <10 ng g^−1^ quantification limit requirements.

## Experimental

### Chemicals and Materials

HPLC-grade acetonitrile (MeCN) was purchased from Fisher Scientific (Pittsburgh, PA; USA) and deionized water of 18.2 MΩ-cm came from a Barnstead/Thermolyne (Dubuque, IA; USA) E-Pure Model D4641. Ammonium formate (HCO_2_NH_4_) was purchased from Sigma-Aldrich (Saint Louis, MO; USA). Pesticide standards were obtained from the Environmental Protection Agency’s National Pesticide Repository (Fort Meade, MD; USA), ChemService (West Chester, PA; USA), or Dr. Ehrenstorfer GmbH (Augsburg; Germany). Standards of 14 flame retardants (FRs), 14 polychlorinated biphenyls (PCBs), and 15 polycyclic aromatic hydrocarbons (PAHs) were purchased from Sigma-Aldrich, AccuStandard (New Haven, CT; USA), and Santa Cruz Biotechnology (Santa Cruz, CA; USA). Table 1 lists all the analytes in the different categories (pesticides, FRs, PCBs, and PAHs).

**Table 1:** Parameters in LPGC-MS/MS analysis of the 94 analytes including three pairs (*CE* collision energy)

Nos.	Analyte	MS/MS segment	*t*_R_ (min)	Quantifier ion (*m/z*)	CE (V)	Qualifier ion (*m/z*)	CE (V)	Internal standard (IS)
Pesticides								
1	Acephate	2	3.275	136 → 42	10	136 → 94	10	Atrazine-*d*_5_
2	Aldrin	4	4.600	263 → 193	40	263 → 228	20	Atrazine-*d*_5_
3	Atrazine	3	3.999	215 → 200	10	215 → 138	10	Atrazine-*d*_5_
4	Bifenthrin	7, 8	5.479	181 → 165	30	181 → 166	15	Atrazine-*d*_5_
5	Carbofuran	3	3.985	164 → 103	25	164 → 149	5	Atrazine-*d*_5_
6	Carbophenothion	6	5.259	342 → 157	5	157 → 75	40	Atrazine-*d*_5_
7	Chlorothalonil	3	4.220	266 → 133	40	266 → 168	25	Atrazine-*d*_5_
8	Chlorpyrifos	4	4.581	199 → 171	20	314 → 258	20	Atrazine-*d*_5_
9	Coumaphos	8, 9	5.890	362 → 109	15	362 → 81	40	Atrazine-*d*_5_
10	Cyfluthrin^a^	8	5.857	163 → 127	5	163 → 91	15	Atrazine-*d*_5_
11	Cypermethrin^a^	9	6.043	181 → 152	20	163 → 91	15	Atrazine-*d*_5_
12	Cyprodinil	4	4.712	224 → 208	25	224 → 197	25	Atrazine-*d*_5_
13	*o*,*p*′-DDE	4	4.867	246 → 176	35	318 → 248	25	Atrazine-*d*_5_
14	*p*,*p*′-DDE	5	5.004	246 → 176	35	318 → 248	25	Atrazine-*d*_5_
15	Deltamethrin^a^	8, 9	6.435	181 → 152	25	181 → 127	25	Atrazine-*d*_5_
16	Diazinon	3	4.111	179 → 137	20	304 → 179	15	Atrazine-*d*_5_
17	Dicrotophos	2	3.828	127 → 109	10	127 → 95	15	Atrazine-*d*_5_
18	Dimethoate	3	3.978	93 → 63	5	125 → 93	15	Atrazine-*d*_5_
19	Diphenylamine	2	3.736	169 → 168	10	169 → 140	40	Atrazine-*d*_5_
20	Endosulfan I	4	4.956	241 → 206	15	241 → 170	30	Atrazine-*d*_5_
21	Endosulfan II	5, 6	5.165	241 → 206	15	241 → 170	30	Atrazine-*d*_5_
22	Endosulfan sulfate	6, 7	5.323	387 → 253	15	387 → 206	30	Atrazine-*d*_5_
23	Esfenvalerate^a^	9	6.259	167 → 125	5	167 → 89	35	Atrazine-*d*_5_
24	Ethalfluralin	2, 3	3.771	276 → 202	15	276 → 105	25	Atrazine-*d*_5_
25	Ethoprop	2	3.749	242 → 158	5	242 → 127	10	Atrazine-*d*_5_
26	Fenpropathrin	7, 8	5.493	181 → 152	20	181 → 77	40	Atrazine-*d*_5_
27	Fipronil	4	4.751	367 → 213	35	367 → 255	20	Atrazine-*d*_5_
28	Flutriafol	4, 5	4.954	219 → 123	20	219 → 95	40	Atrazine-*d*_5_
29	Heptachlor	4	4.430	272 → 237	20	272 → 117	35	Atrazine-*d*_5_
30	Heptachlor epoxide	4	4.775	353 → 263	15	353 → 253	25	Atrazine-*d*_5_
31	Heptenophos	2	3.611	124 → 89	10	124 → 63	35	Atrazine-*d*_5_
32	Hexachlorobenzene (HCB)	3	3.981	284 → 214	35	284 → 249	20	Atrazine-*d*_5_
33	Imazalil	5	4.986	215 → 173	10	215 → 145	30	Atrazine-*d*_5_
34	Kresoxim-methyl	5	5.019	116 → 89	20	206 → 131	20	Atrazine-*d*_5_
35	Lindane (γ-BHC or γ-HCH)	3	4.092	219 → 183	5	183 → 147	15	Atrazine-*d*_5_
36	Methamidophos	1	2.855	141 → 95	5	95 → 79	10	Atrazine-*d*_5_
37	Methoxychlor	7, 8	5.507	227 → 169	30	227 → 141	30	Atrazine-*d*_5_
38	Mirex	8	5.728	272 → 237	15	272 → 143	40	Atrazine-*d*_5_
39	Myclobutanil	5	5.016	179 → 125	15	179 → 90	35	Atrazine-*d*_5_
40	*cis*-Nonachlor	6	5.208	409 → 300	25	409 → 145	15	Atrazine-*d*_5_
41	*trans*-Nonachlor	*5*	4.962	409 → 300	25	409 → 145	15	Atrazine-*d*_5_
42	Omethoate	2	3.666	110 → 79	15	156 → 110	5	Atrazine-*d*_5_
43	Penconazole	4	4.742	159 → 89	35	248 → 157	25	Atrazine-*d*_5_
44	Pentachlorothioanisole	3	3.996	280 → 237	20	280 → 265	10	Atrazine-*d*_5_
45	Permethrin (*cis* + *trans*)^a^	8, 9	5.835	183 → 168	15	183 → 153	15	Atrazine-*d*_5_
46	*o*-Phenylphenol	2	3.485	170 → 115	35	170 → 141	20	Atrazine-*d*_5_
47	Piperonyl butoxide	6, 7	5.357	176 → 103	25	176 → 131	10	Atrazine-*d*_5_
48	Propargite	6, 7	5.353	135 → 107	10	135 → 95	10	Atrazine-*d*_5_
49	Pyridaben	8, 9	5.865	147 → 117	20	147 → 91	40	Atrazine-*d*_5_
50	Pyriproxyfen	8	5.634	136 → 78	30	136 → 96	10	Atrazine-*d*_5_
51	Tebuconazole	6, 7	5.357	251 → 125	20	251 → 127	25	Atrazine-*d*_5_
52	Tetraconazole	4	4.599	336 → 218	15	336 → 156	30	Atrazine-*d*_5_
53	Thiabendazole	4	4.939	201 → 174	15	174 → 65	30	Atrazine-*d*_5_
54	Tribufos	5	4.982	169 → 113	10	169 → 95	30	Atrazine-*d*_5_
Flame retardants (FRs)								
1	BDE 183	10	7.205	720 → 562	20	720 → 560	20	FBDE 126
2	Dechlorane plus (syn and anti)^a^	11	8.300	272 → 237	20	272 → 235	20	FBDE 126
3	PBB 153	9	6.315	468 → 308	40	468 → 310	30	FBDE 126
4	Pentabromoethylbenzene (PBEB)	6, 7	5.289	500 → 406	40	500 → 261	40	Atrazine-*d*_5_
5	Pentabromotoluene (PBT)	6	5.210	486 → 407	30	486 → 326	30	FBDE 126
6	2-Ethylhexyl-2,3,4,5-tetrabromobenzoate (TBB)	8, 9	5.994	421 → 393	10	421 → 314	20	FBDE 126
7	1,2,5,6-Tetrabromocyclooctane (TBCO)	4, 5	4.901	267 → 105	30	267 → 91	30	Atrazine-*d*_5_
8	1,2-Dibromo-4-(1,2-dibromoethyl) cyclohexane (TBECH)	4	4.702	267 → 79	40	267 → 105	40	Atrazine-*d*_5_
9	Tribromoneopentyl alcohol (TBNPA)	2	3.665	214 → 133	10	214 → 135	10	Atrazine-*d*_5_
10	1,2,4,5-Tetrabromo-3,6-dimethylbenzene (TBX)	4	4.809	422 → 102	40	422 → 182	40	Atrazine-*d*_5_
11	*tris*(2-chloroethyl)phosphate (TCEP)	3	4.015	249 → 63	20	249 → 99	20	Atrazine-*d*_5_
12	*tris*(1-chloro-2-propyl)phosphate (TCPP)	3	4.127	277 → 125	20	277 → 99	20	Atrazine-*d*_5_
13	*tris*(1,3-dichloro-2-propyl)phosphate (TDCPP)	6, 7	5.248	381 → 159	20	381 → 79	20	Atrazine-*d*_5_
14	Triphenyl phosphate (TPP)	6, 7	5.380	326 → 169	30	326 → 77	30	Atrazine-*d*_5_
Polycyclic aromatic hydrocarbons (PAHs)								
1	Acenaphthene	2	3.465	153 → 152	30	153 → 151	30	Acenaphthalene-*d*_8_
2	Acenaphthylene	2	3.380	152 → 151	30	152 → 126	20	Acenaphthylene-*d*_8_
3	Anthracene	4	4.183	178 → 176	40	178 → 152	40	Phenanthrene-*d*_10_
4	Benz(a)anthracene + chrysene	7, 8	5.563	228 → 226	25	228 → 227	25	Pyrene-*d*_10_
5	Benzo(a)pyrene	9	6.231	252 → 250	40	252 → 226	40	Benzo(a)pyrene-*d*_12_
6	Benzo(b + k)fluoranthene	8	6.064	252 → 250	40	252 → 226	40	Benzo(a)pyrene-*d*_12_
7	Benzo(g,h,i)perylene	10	7.115	276 → 274	40	276 → 275	40	Benzo(g,h,i)perylene-*d*_12_
8	Dibenz(a,h)anthracene	10	6.985	278 → 252	40	278 → 250	40	Benzo(a)pyrene-*d*_12_
9	Fluoranthene	4	4.809	202 → 200	40	202 → 201	40	Fluoranthene-*d*_10_
10	Fluorene	2	3.675	165 → 164	30	165 → 163	30	Phenanthrene-d10
11	Indeno(1,2,3-c,d)pyrene	10	6.926	276 → 274	30	276 → 275	30	Benzo(g,h,i)perylene-*d*_12_
12	Naphthalene	1	2.775	128 → 102	20	128 → 127	20	Naphthalene-*d*_8_
13	Phenanthrene	3	4.160	178 → 176	40	178 → 152	40	Phenanthrene-*d*_10_
14	Pyrene	5	4.943	202 → 200	40	202 → 201	40	Pyrene-*d*_10_
Polychlorinated biphenyls (PCBs)								
1	PCB 77	6	5.049	292 → 222	40	292 → 220	25	^13^C_12_-PCB 153
2	PCB 81	5	5.010	292 → 222	40	292 → 220	25	^13^C_12_-PCB 153
3	PCB 105	7	5.251	326 → 256	30	326 → 254	30	^13^C_12_-PCB 153
4	PCB 114	6	5.193	326 → 256	30	326 → 254	30	^13^C_12_-PCB 153
5	PCB 118 + 123	5	5.144	326 → 254	30	326 → 256	30	^13^C_12_-PCB 153
6	PCB 126	7	5.366	326 → 256	40	326 → 254	40	^13^C_12_-PCB 153
7	PCB 156 + 157	7	5.538	360 → 288	40	360 → 290	40	^13^C_12_-PCB 153
8	PCB 167	8	5.421	360 → 290	40	360 → 288	40	^13^C_12_-PCB 153
9	PCB 169	8	5.650	360 → 288	40	360 → 290	40	^13^C_12_-PCB 153
10	PCB 170	7, 8	5.695	394 → 324	40	394 → 322	40	^13^C_12_-PCB 153
11	PCB 180	8	5.575	394 → 324	40	394 → 322	40	^13^C_12_-PCB 153
12	PCB 189	9	5.786	394 → 324	40	394 → 322	40	^13^C_12_-PCB 153
Internal standards (IS) and quality control (QC) standard								
1	^13^C_12_-PCB 153 (IS)	6	5.185	372 → 302	40			
2	Acenaphthalene-*d*_8_ (IS)	2	3.339	160 → 158	30			
3	Atrazine-*d*_5_ (IS)	3	3.956	205 → 105	15			
4	Benzo(a)pyrene-*d*_12_ (IS)	8, 9	6.261	264 → 260	40			
5	Benzo(g,h,i)perylene-*d*_12_ (IS)	10	7.122	288 → 284	40			
6	FBDE 126 (IS)	9	6.223	582 → 315	40			
7	Fenthion-*d*_6_ (IS)	4	4.500	284 → 115	20			
8	Fluoranthene-*d*_10_ (IS)	4	4.89	212 → 208	40			
9	Naphthalene-*d*_8_ (IS)	1	2.744	136 → 80	40			
10	Phenanthrene-*d*_10_ (IS)	3	4.11	188 → 160	20			
11	Pyrene-*d*_10_ (IS)	5	4.89	212 → 210	40			
12	*p*-Terphenyl-*d*_14_ (QC)	5	4.985	244 → 212	40			

All ion transitions used wide resolution setting and 4 ms dwell times. Segment start times were: 1 = 2.35 min; 2 = 3.1; 3 = 3.9; 4 = 4.35; 5 = 4.9; 6 = 5.15; 7 = 5.35; 8 = 5.5; 9 = 6; 10 = 6.75; and 11 = 8; end 9 min

^a^Multiple peaks

A working standard of the 97 pesticides and environmental contaminants was prepared at 5 ng µL^−1^ (0.5 ng µL^−1^ for PCBs) in MeCN solution. This mixed standard served as the highest level spiking solution (100 ng mL^−1^ in extracts), and it was also used to prepare the 2.5, 1.25, and 0.5 ng µL^−1^ standard solutions for lower spiking levels (50, 25, and 10 ng mL^−1^, respectively).

For use as internal standards (IS), atrazine-*d*_5_ and fenthion-*d*_6_ were from C/D/N Isotopes (Pointe-Claire, Quebec; Canada). ^13^C_12_-PCB 153 and a PAH mixture of acenaphthylene-*d*_8_, benzo[a]pyrene-*d*_12_, benzo[g,h,i]perylene-*d*_12_, fluoranthene-*d*_10_, naphthalene-*d*_8_, phenanthrene-*d*_10_, and pyrene-*d*_10_ were purchased from Cambridge Isotope Laboratories (Andover, MA; USA). Another IS, 5′-fluoro-3,3′,4,4′,5-pentabromodiphenyl ether (FBDE 126), was obtained from AccuStandard. The IS mixture solution was prepared in MeCN solution at 5 ng µL^−1^ except 0.5 ng µL^−1^ for ^13^C_12_ PCB 153.

Analyte protectants (APs) [19] containing 25 mg mL^−1^ ethylglycerol, 2.5 mg mL^−1^ each of gulonolactone and d-sorbitol, and 1.25 mg mL^−1^ shikimic acid (all from Sigma-Aldrich) was prepared in 3/2 (v/v) MeCN/water containing 1.1 % formic acid to enhance pesticide stability of final extract [20]. As a post-cleanup quality control (QC) standard, *p*-terphenyl-*d*_14_ (from AccuStandard) was included in the APs mixture at 0.88 ng µL^−1^.

Eleven different food matrices including Gala apple, kiwi, kale, carrot, navel orange, canned black olives, wheat grain, dried basil, pork loin, salmon, and avocado were purchased from local grocery stores. The samples were comminuted with dry ice using a Robot Coupe (Ridgeway, MS; USA) RSI 2Y1 chopper and stored in glass jars at −20 °C until analysis.

### Sample Extraction

Comminuted samples of apple, kiwi, carrot, kale, orange, black olive, pork loin, salmon, and avocado (15 g) were individually weighed in 50 mL polypropylene tubes along with 7.5 g HCO_2_NH_4_, which was extracted for 10 min with 15 mL MeCN using a Glas-Col (Terre Haute, IN; USA) platform pulse mixer at 80 % setting with maximum pulsation. For wheat grain and dried basil, 5 g sample + 15 mL water + 7.5 g HCO_2_NH_4_ was added to the tubes with 15 mL MeCN and extraction time was 60 min using the platform shaker (capacity of 50 tubes at a time). For reagent blanks, 15 mL water represented the sample. Then, centrifugation at 4150 rpm (3711 rcf) at room temperature for 3 min was conducted using a Thermo Fisher (Waltham, MA; USA) Sorvall Legend RT centrifuge (capacity of twenty 50 mL tubes at a time). Extracts of individual matrices were combined and spiked (or not) with the analytes and IS to evaluate the automated mini-SPE cleanup step. The initial extracts (spiked or not) were transferred to 1.8 mL standard ambler glass autosampler (AS) vials, which were closed with split septa caps.

### Automated Mini-SPE Cleanup

As previously described [12], two different types of SPE mini-cartridges were purchased for evaluation from ITSP solutions (Hartwell, GA; USA): (1) 45 mg anh. MgSO_4_/primary secondary amine (PSA)/C_18_/CarbonX (in the ratio of 20/12/12/1, w/w/w/w, respectively); and (2) 30 mg C_18_/Z-Sep/CarbonX (20.7/8.3/1, w/w/w, respectively). The mini-cartridges, as shown in the Table of Contents graphic and Supplemental information, were 3.5 cm long with a 0.8 cm diameter.

Automated mini-SPE was conducted using a Gerstel (Linthicum, MD; USA) robotic MultiPurpose Sampler (MPS) liquid handling system [also known as a PAL3-RTC from CTC Analytics (Zwingen, Switzerland)]. PAL Sample Control (CTC Analytics) software was used to program and operate the device. The steps in the final automated mini-SPE cleanup method using the 45 mg 4-sorbent mixture (#1 above) are shown in Table 2. For matrix-matched calibration standards, Step 9 to add 25 µL MeCN was not done (Step 10 to add the APs + QC solution was still done), and instead, 25 µL of the appropriate calibration standard solutions were added manually to the matrix blank extracts.

**Table 2:** Steps and time for the 8 min automated mini-SPE method

Step	Description	Time (s)
1	Wash the 1 mL syringe with MeCN (2 pumps of 0.5 mL each)	30
2	Load 300 µL extract from AS vial in Tray1 into 1 mL syringe	10
3	Place mini-cartridge above collection AS vial (with glass insert) in Tray2	10
4	Elute extract through mini-cartridge at 2 µL s^−1^	150
5	Discard mini-cartridge into waste receptacle	5
6	Wash the 1 mL syringe with 1/1/1 MeCN/MeOH/water (2 pumps of 0.5 mL each)	30
7	Wash the 1 mL syringe with MeCN (4 pumps of 0.5 mL each)	45
8	Switch to 100 µL syringe and wash with MeCN (2 pumps of 50 µL each)	50
9	Add 25 µL MeCN to collection AS vial (with glass insert) in Tray2	10
10	Add 25 µL AP + QC sol’n to collection AS vial (with glass insert) in Tray2	10
11	Wash the 100 µL syringe with 1/1/1 MeCN/MeOH/water (5 pumps of 50 µL each)	50
12	Wash the 100 µL syringe with MeCN (3 pumps of 50 µL each)	40
13	Switch to 1 mL syringe and move to home position	40

The cleanup itself only took ≈3 min, and syringe wash and exchange steps took ≈5 min

Procedurally, the AS vials containing the initial extracts were placed into a 54 position tray (Tray1), and the corresponding collection AS vials with glass micro-inserts (300 µL) and split septa caps were placed into a second 54 position tray (Tray2). An ITSP vial guide cover for the mini-cartridges was placed atop the vials in Tray2. A third tray (Tray3) contained 96 mini-cartridges placed above a solvent waste drain (if pre-conditioning with solvent is desired). The same tray holder contained all 3 trays, and our system was fitted with two tray holders for a potential capacity of 108 samples for sequential unattended automated cleanup.

The robotic liquid handler was fitted with 3 interchangeable glass syringes (gastight with 57 mm long, 22 gage, straight tip needles) in different slots: 10, 1, and 0.1 mL. As shown in Table 2, only the 1 and 0.1 mL syringes were used in the experiments, and if we had chosen to install the device onto the LPGC-MS/MS instrument as designed as an option by the manufacturers, we would have chosen to replace the 10 mL syringe with a 5–10 µL syringe for direct injection of final extracts after cleanup. Instead, we chose to use the device in stand-alone fashion at this time (greater flexibility in independent operations for both LC and GC analyses).

The collection vials were pre-weighed to the nearest mg prior to and after conducting mini-SPE, and weight differences were recorded to assess consistency of the liquid transfers and final extract volumes. After weighing and preparation of calibration standards, the vials were recapped with non-slit septa, vortexed ≈1 s to mix, and placed on the autosampler tray for LPGC-MS/MS analysis. Equivalent sample concentrations of final extracts dehydrated by anh. MgSO_4_ in mini-SPE were 1 g mL^−1^ for fruits and vegetables, and 0.33 g mL^−1^ for wheat grain and dried basil.

### Fast LPGC-MS/MS Analysis

Table 1 lists the 97 targeted analytes (three analyzed together) plus 12 internal and quality control standards and their conditions in LPGC-MS/MS analysis. An Agilent (Little Falls, DE; USA) 7890A/7010 gas chromatograph/triple quadrupole mass spectrometer was employed using electron ionization (EI) at −70 eV and 100 µA filament current. The separation was achieved on a 15 m × 0.53 mm i.d. × 1 µm film thickness Phenomenex (Torrance, CA; USA) ZB-5MSi analytical column connected using an Agilent Ultimate union to a 5 m × 0.18 mm, i.e., uncoated restrictor/guard column from Restek (Bellefonte, PA, USA). The calculated virtual column length was 5.5 m × 0.18 mm, i.e., and constant flow rate of He (99.999 %) carrier gas was 2 mL min^−1^. Details about the theory and practice of LPGC-MS/MS have been reported previously [17].

The GC oven temperature program was 70 °C for 1.5 min, ramped at 80–180 °C min^−1^, then 40–250 °C min^−1^, followed by 70–320 °C min^−1^, held for 4.4 min (10.025 min total). Cool down and re-equilibration time was 3 min. The transfer line temperature was 280 °C, ion source was 320 °C, and quadrupoles were kept at 150 °C. Collision gas flow rate was 1.5 mL min^−1^ N_2_ and quench gas was 2.25 mL min^−1^ He. The Agilent multimode inlet conditions were the same as reported previously [10, 18], except injection volume was reduced to 1.0 µL plugged between 1.5 μL air above and below in the syringe. An Agilent Ultra-inert 2 mm dimpled splitless liner was placed in the inlet and Agilent Mass Hunter version B07 software was used for instrument control and data processing.

When using APs, extensive post-injection washing of the syringe with aqueous solution is very important to avoid sticking of the plunger if the sugar derivatives precipitate onto surfaces. In this study, the wash steps of the 10 µL syringe entailed 10 × 5 µL of 1/1/1 (v/v/v) MeCN/MeOH/water followed by 10 × 5 µL of MeCN after every injection. The pre-injection wash steps called for another 5 µL of the aqueous wash solution and 2 × 5 µL of MeCN, each contained in 100 mL wash bottles.

### Method Optimization and Validation Experiments

To evaluate the consistency and performance of the automated mini-SPE application, and to assess cleanup efficiency and analyte retention vs. elution volumes for the two different cartridges, the 97 analytes were spiked into extracts of four different matrices (kale, avocado, pork, and salmon). Spiking level was 100 ng mL^−1^ (10 ng mL^−1^ for PCBs), and five different volumes (200, 300, 400, 500, and 600 µL) of extracts in triplicate were loaded into the cartridges at 2 µL s^−1^. Proportional amounts of AP + QC mix were added to the final extracts depending on elution volumes, and the cleanup efficiency was compared in full scan LPGC-MS and UV–Vis measurements. A Synergy HT Multi-detection microplate reader from Bio-Tek (Winooski, VT; USA) was used for optical density measurements. Relative recoveries of the analytes vs. extract load/elution volumes were measured using LPGC-MS/MS.

The final mini-SPE cleanup method was validated by spiking QuEChERS extracts of 10 sample matrices (Gala apple, kiwi, kale, carrot, navel orange, canned black olive, wheat grain, dried basil, pork loin, and salmon) at 10, 25, 50, and 100 ng mL^−1^ (ten times lower for PCBs) with *n* = 4 at each level and matrix. A pair of chemists prepared and analyzed 47 samples (65 injections) of two matrices per day (LPGC-MS/MS sequences took ≈14 h). The recoveries and RSDs were determined from peak areas generated from summation function integration normalized (or not, for comparison purposes) to the corresponding IS listed in Table 1 for each analyte. Matrix-matched (MM) and reagent-only (RO) calibration standards (7 levels each: 0, 5, 10, 25, 50, 100, and 150 ng mL^−1^; tenfold lower for PCBs) were prepared by adding 25 µL calibration standards to final matrix blank extracts (MM) or 220 µL MeCN + 25 µL AP + QC solution (RO). The calibration solutions in MeCN each contained 0.88 ng µL^−1^ IS and 0, 0.044, 0.088, 0.22, 0.44, 0.88, and 1.32 ng µL^−1^ concentrations of the analytes (tenfold lower for PCBs and ^13^C_12_-PCB 153 as the IS) to yield the seven levels listed above. Matrix effects (MEs) were calculated as the % difference in least-linear squared calibration slopes of the MM vs. RO calibration standards.

## Results and Discussion

### LPGC-MS/MS Analysis

In previous LPGC-MS/MS studies in our group, we used a 7000A MS/MS instrument [10, 21], which was upgraded to a 7000B [13, 18], and now to a 7010 system. In regulatory monitoring of pesticide residues in foods, the need of analysis calls for LOQ <10 ng g^−1^ [22]. The injection of the least amount of sample equivalent to meet the desired LOQ improves the ruggedness of the method and reduces instrument maintenance demands. Originally with the 7000A instrument, 10 mg sample equivalent (1 mg µL^−1^ QuEChERS extracts in MeCN) was injected [10, 21], which was reduced to 2.5 mg when using the 7000B [18]. In this study, the 7010 upgrade enabled injection of 1 mg (1 µL) sample equivalent to still achieve <5 ng g^−1^ LOQs for all analytes in the fruit and vegetable matrices. Cold inlet conditions using programmable temperature vaporization were used in each method thus far, but in the near future, we plan to investigate hot split mode injections to further speed the analysis, possibly improve performance, and reduce the amount of matrix components being introduced into the instrument [23].

We started to estimate LOQs from the final method in this study, but recognized quickly that the calculations were inaccurate due to ultra-trace carry over or artifacts that infiltrated the background for nearly all analytes in MeCN, reagent, and matrix blanks. The carry over averaged 0.4 % of the previous injection, and likely originated from the injection syringe needle contaminating the 100 mL wash bottles on the autosampler, not from the mini-SPE procedure which prepared 14 blank extracts prior to the reagent blank in its sequence.

Peak integration was conducted using the summation function in the Agilent MassHunter software, which simply drew a baseline at the lowest point between analyst-defined start and stop times to cover the *t*_R_ and peak width for each analyte. The integration start and stop times were set by ensuring that they fully covered the analyte peaks for all 325 injections over the course of all five sequences in the validation study. The peak area was always positive using this software’s summation integration function, which led to positive responses even when only noise was present. However, inspection of the MRM chromatograms and ion ratios clearly indicated that the background peaks in reagent and solvent blanks originated from the analytes in most cases, not simply electronic or chemical noise. The equivalent background concentrations of analytes in RO blanks averaged 0.5 ng mL^−1^ for the pesticides, FRs, and PAHs, and 0.05 ng mL^−1^ for PCBs, which did not affect the results of the study, but also did not permit an accurate estimation of LOQs. The likely source of carryover can be eliminated using a newer type of flowing solvent wash station that rinses the analytes coating the needle to waste rather than allowing them to contaminate the fixed volume wash solutions. LOQs can be better estimated after this issue is resolved, but the 1 µL injection clearly met analytical purposes in this application. We can conclude that the LPGC-MS/MS method yielded LOQs <0.5 ng g^−1^ (<0.05 ng g^−1^ for PCBs) for all but a few analytes, such as fenpropathrin, endosulfans, and BDE 183 which gave LOQs ≈1 ng g^−1^.

### Automated Mini-SPE

Morris and Schriner [12] reported that mini-SPE cartridges containing 20 mg anh. MgSO_4_, 12 mg PSA and C_18_ each, and 1 mg CarbonX (45 mg total) provided satisfactory cleanup and recoveries for a wide range of pesticides and matrices in GC-MS/MS analysis. For LC-MS/MS, they chose to use cartridges containing 20.7 mg C_18_, 8.3 mg Z-Sep, and 1 mg CarbonX (30 mg total), which provided acceptable cleanup and pesticide recoveries for monitoring purposes [12]. In their method, they pre-conditioned the mini-cartridge with 150 µL MeCN, added 150 µL QuEChERS extract, then eluted with another 150 µL elution solvent.

In d-SPE of QuEChERS extracts, no pre-conditioning or extra solvent elution steps are done, and the MeCN extract itself serves as the elution solvent. Ideally, the sorbents only adsorb co-extracted matrix components and not analytes. We decided to streamline and speed the method of Morris and Schriner [12] by eliminating the MeCN pre-conditioning and solvent elution steps for the same mini-cartridges they developed, which are commercially available. They conducted very thorough studies to set the sorbent combinations, and we had previously found similar sorbent mixtures work well for cleanup and analysis pesticides and environmental contaminants in seafood matrices using filter-vial d-SPE [13]. We have since extended the filter-vial d-SPE method to other animal tissues (cattle, pork, and chicken muscle), but found that only filtering of initial extracts was needed for analysis of the 99 LC-amenable pesticides in our recent approach [18].

**Fig. 1:**
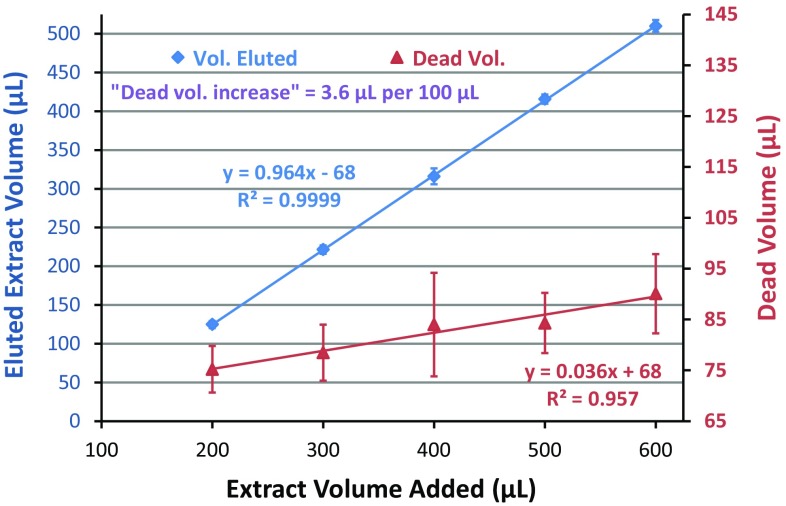
Eluted extract and dead volumes of the mini-SPE cartridges in cleanup of QuEChERS extracts of kale, avocado, pork, and salmon (*n* = 12 for each extract load volume)

In this evaluation, we focused on 97 representative GC-amenable analytes and compared the two different mini-cartridges for their analysis in QuEChERS extracts of kale, salmon, pork, and avocado, and using extract load volumes of 200, 300, 400, 500, and 600 µL. To determine elution and dead volumes of the mini-cartridges, weights of the collection vials were compared before and after cleanup. Figure 1 shows the results in which measured dead volume (based on measured solution densities) was found to increase slightly as more extract was loaded into the mini-cartridges at 2 µL s^−1^. The reason for this observance was very likely because more interstitial spaces in the sorbent beds were being filled as more solution was passed through the cartridges. In any case, the dead volumes were much the same for both types of mini-cartridges, and averaged 75 ± 5 to 90 ± 8 µL from 200 to 600 µL extract load volumes, respectively. Thus, the measured extract elution volumes were 125 ± 5 to 510 ± 8 µL from 200 to 600 µL extract load volumes, respectively, which provided known and rather consistent elution volumes in the method.

In the validation study involving 235 mini-SPE cartridges, final extracts averaged 278 ± 5 μL (1.9 % RSD), in which only 4 final extracts had ≈25 μL reduced volume. The robotic liquid handler operated flawlessly throughout the study, and perhaps vial weights were transcribed improperly, bubbles materialized in the syringe, or mini-cartridge dead volumes were larger, which caused those four outliers.

### Mini-SPE Cleanup

Full scan LPGC-MS chromatograms of the reagent blank, kale, avocado, salmon, and pork QuEChERS extracts were compared before and after the mini-SPE cleanup using the 200–600 µL load volumes. Results indicated that the 30 mg C_18_/Z-Sep/CarbonX (20.7/8.3/1, w/w/w) mini-cartridges provided little cleanup for any of the 4 matrices independent of extract load/elution volumes (see Supplemental information). The chromatograms appeared much the same in each injection. These mini-cartridges were devised for LC-MS/MS [12], and this LPGC-MS result is probably due to the lack of anh. MgSO_4_ in the cartridges to reduce water content in the final extracts. Density measurements of final extracts indicated that the 45 mg mini-cartridges containing 20 mg anh. MgSO_4_ led to cleaned extracts (before addition of AP + QC solution) of ≈2 % water (0.79 mg μL^−1^ density of the MeCN extracts) and the 30 mg cartridges without anh. MgSO_4_ maintained the same extract density (0.82 mg μL^−1^) as the initial extracts, which were ≈15 % moisture. Drier solutions are better for GC analyses and provide stronger adsorption properties for common sorbents (e.g. PSA, Z-Sep, silica, Florisil, Alumina).

**Fig. 2:**
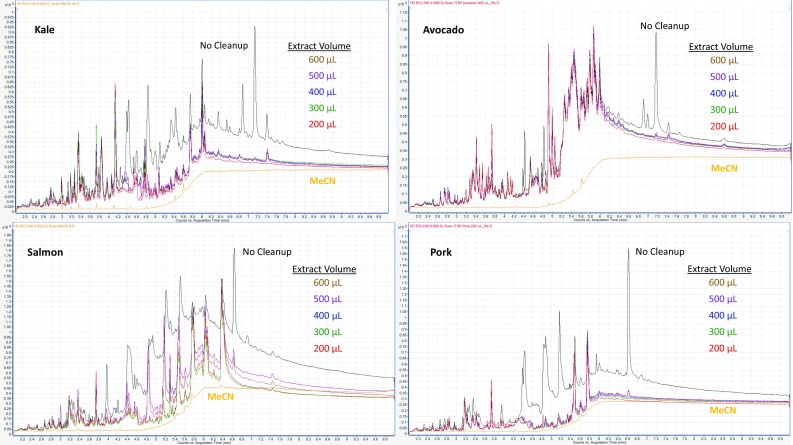
Total ion chromatograms in full scan LPGC-MS of the QuEChERS extracts of 4 matrixes before and after cleanup with the 45 mg mini-SPE cartridges using different extract load volumes

In the case of the 45 mg mini-cartridges of 20/12/12/1 anh. MgSO_4_/PSA/C_18_/CarbonX, significant removal of matrix co-extractives was observed in the LPGC-MS chromatograms of kale, pork, and salmon, but little difference was observed for avocado (see Fig. 2). As would be expected, greater cleanup took place using smaller load/elution volumes before breakthrough began to occur. Depending on analyte recoveries, 200–300 µL would be preferably chosen to provide more cleanup and shorter time than achieved with larger volumes.

**Fig. 3:**
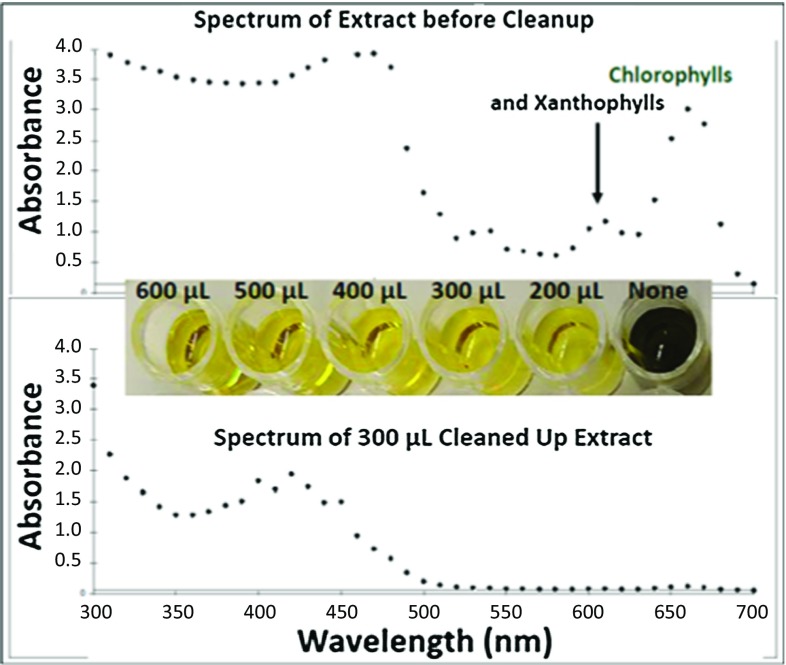
Comparison of the UV–Vis spectra before and after cleanup of kale extracts using the 45 mg anh. MgSO_4_/PSA/C_18_/CarbonX mini-SPE cartridges

Unlike LPGC-MS, UV–Vis absorbance spectrometry of the 200–600 µL final extracts (200 µL of combined replicates in 96-well plates) showed similar cleanup efficiencies using either the 45 or 30 mg mini-cartridges (see Supplemental information). As shown in Fig. 3, chlorophyll and xanthophyll co-extractives from the kale in particular were nearly eliminated by the sorbents, mainly due to CarbonX. Salmon and pork extracts were nearly colorless, and measured UV–Vis cleanup efficiency was small compared to full scan LPGC-MS results. In the case of avocado as shown in Fig. 2, LPGC-MS showed little differences in full scan chromatograms, but UV–Vis spectra exhibited strong reduction in absorbance readings from 300 to 700 nm (see Supplemental information). Kale co-extractives were dramatically reduced in both types of measurement; especially by the 45 mg mini-cartridges (see Figs. 2, 3).

### Analyte Retention in Mini-SPE

**Fig. 4:**
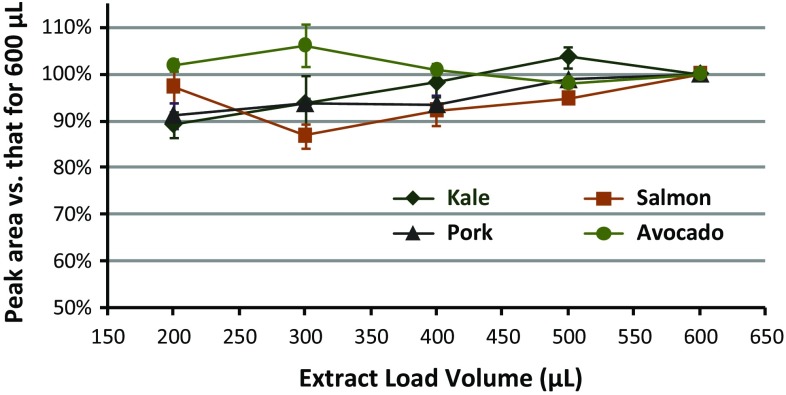
Relative recoveries of bifenthrin in extracts of the 4 matrices (100 ng mL^−1^,* n* = 3 each) after automated cleanup using the 45 mg mini-cartridges

**Fig. 5:**
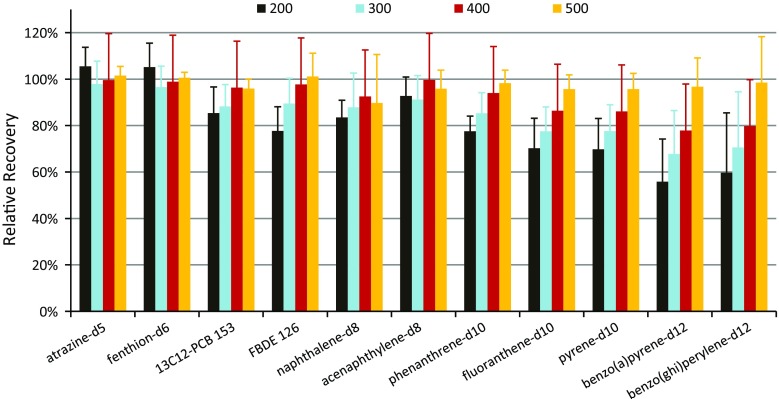
Relative recoveries vs. the 600 μL extract load volumes of the 11 IS spiked at 100 ng mL^−1^ in the avocado, kale, pork, and salmon extracts using different load volumes with the 45 mg mini-cartridges (*n* = 12 each)

**Fig. 6:**
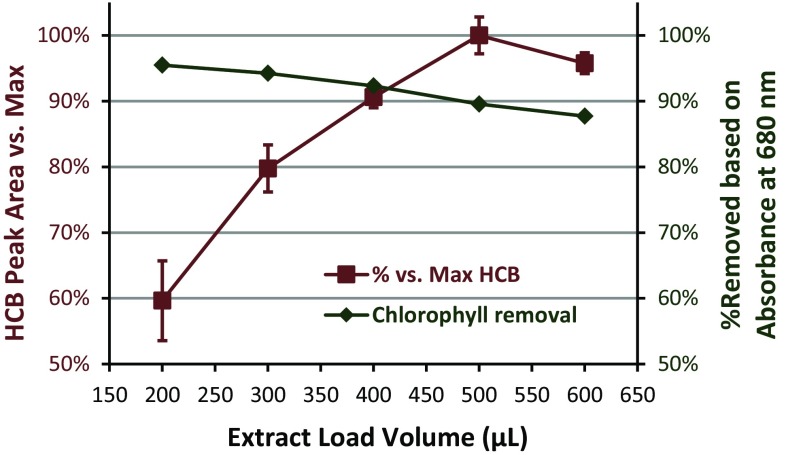
Elution of hexachlorobenzene (HCB) and removal of chlorophyll from kale extracts passed through the 45 mg anh. MgSO_4_/PSA/C_18_/CarbonX mini-SPE cartridges

The extract elution volume not only affected the cleanup efficiency in mini-SPE, but analyte recoveries also depend on load/elution volume. LPGC-MS/MS was used to compare relative recoveries (w/o using IS) normalized to the average 600 μL result with respect to extract load volumes in triplicate for each of the four matrices. Figure 4 shows an example of typical results that were representative of nearly all of the pesticides, demonstrating that neither load/elution volume nor matrix made a significant difference in recoveries. The 30 mg 3-sorbent mixture mini-cartridges also gave similar analyte results as the 45 mg 4-sorbent mini-cartridges. Figure 5 shows the results for the IS with respect to extract load volumes. Only PAHs and a few other analytes with co-planar chemical structures gave less than complete elution, mainly due to partial retention on CarbonX. Hexachlorobenzene (HCB) is one the few pesticides that gave incomplete elution at 200 μL extract load volume. Figure 6 shows how 300 μL load volume (≈220 μL elution) yielded ≈80 % relative HCB recovery while still removing ≈95 % of co-extracted chlorophyll from initial QuEChERS extracts of kale. PBDE 183 and co-planar PCBs (126 and 169) yielded similar results as HCB, but large PAHs (≥3 rings) did not fully elute even with >500 μL load volumes. Similar results were achieved with the 30 mg mini-cartridges, indicating that 1 mg CarbonX was mostly responsible for the retention of the PAHs. As shown in Supplemental information, no significant differences were observed in the relative recoveries for the analytes when using either type of mini-cartridge. Independent of extract volumes, isotopically labeled IS of several PAHs were expected to help compensate for reduced actual recoveries in the final method (see Fig. 5).

Considering the notable cleanup efficiency observed in both UV–Vis and LPGC-MS for the 45 mg mini-cartridges, especially at smaller load/elution volumes, and lack of retention of most analytes in QuEChERS extracts, we chose to use 300 µL load volumes with those mini-cartridges in the final method.

### Method Validation and Summation Integration

For validation of the automated high-throughput cleanup and LPGC-MS/MS method, 10 representative food matrices were chosen using SANTE/11945/2015 [22] as a guide. The final method was conducted for two matrices per day in the following sequence: Day (1) Gala apple and kiwi; (2) carrot and kale; (3) navel orange and canned black olive; (4) wheat grain and dried basil; and (5) pork loin and salmon. QuEChERS has been extensively validated [24] and implemented in many labs [25], and to isolate the mini-SPE cleanup step in this study, initial QuEChERS extracts were spiked (or not) with the 97 analytes at 10, 25, 50, and 100 ng mL^−1^ (*n* = 4 at each level and matrix). Each sequence began with seven blanks of each matrix, which were used for MM calibration standards, followed by the reagent blank and 16 spiked extracts of each matrix, for a total of 47 samples daily, and 235 altogether in 5 days sequentially. The LPGC-MS/MS sequences were conducted each day overnight, which included an additional 7 RO standards conducted at the beginning and end of each sequence, plus a system suitability standard as the first injection and 3 MeCN blanks injected after the 150 ng mL^−1^ (ng g^−1^) RO standards to check for carry over. A total of 65 LPGC-MS/MS analyses were conducted each night, and 325 within 5 days. The same injection liner and autotune parameters were used in each sequence.

**Fig. 7:**
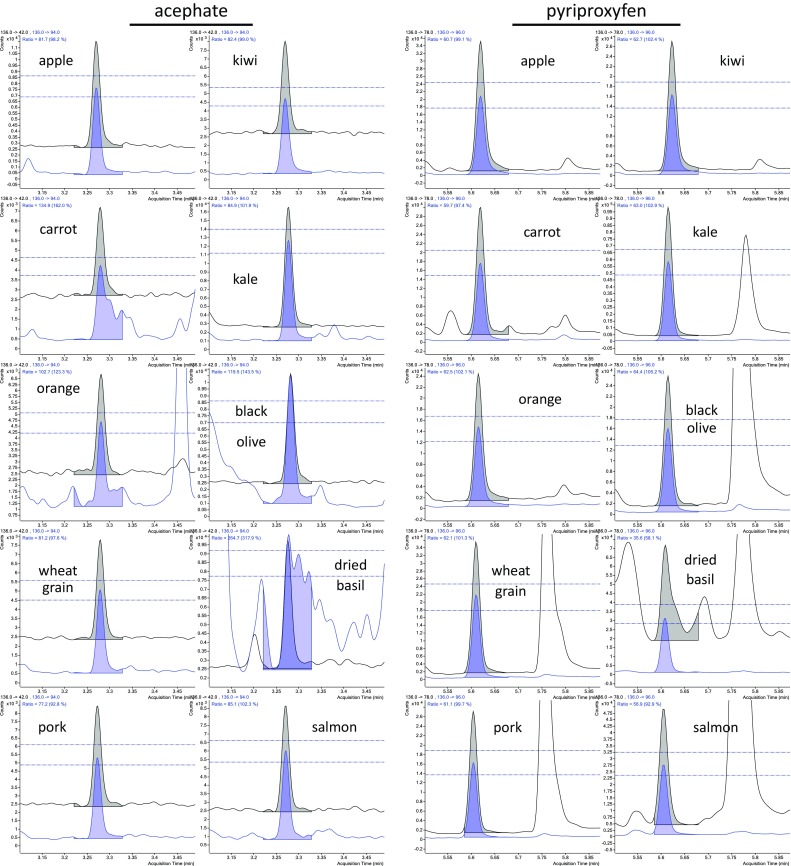
Summation integration of 10 ng mL^−1^ spikes for acephate and pyriproxyfen in the 10 matrices over the course of 5 days. The qualifier ion met the ±10 % ratio relative to the fixed reference ratio for all, but acephate in black olive and dried basil in these examples, as shown by the* horizontal lines*

Summation integration function start and stop times for each analyte were set after the conclusion of all 325 analyses by ensuring that all analyte peaks fell within the integration windows. The use of APs in LPGC-MS/MS led to highly consistent *t*_R_ and peak shapes for all analytes, and relatively few chemical interferences in the chromatograms for each MS/MS ion transition provided leeway to each side of the peak without significantly impacting the integrated peak areas. Figure 7 and Supplemental information show many examples of how the summation integration approach worked for different analytes and matrices. Dechlorane plus, (es)fenvalerate, permethrin, deltamethrin, cyfluthrin, and cypermethrin gave multiple peaks with the same ion transitions, several of which could have been integrated separately, but we chose to integrate them together as a single analyte. Sapozhnikova and Lehotay previously presented several examples of LPGC-MS/MS separations of different closely eluting analytes with isobaric ion transitions comparing columns from different manufacturers [17] (Figs. 4, 5, 6).

The Agilent summation function does not find the deflection point between unresolved peaks, which does not always occur at the exact same time in each chromatogram. Thus, benz(a)anthracene + chrysene and PCBs 118 + 123 and 156 + 157 could be distinguished individually by *t*_R_, but were integrated together because they closely co-eluted. We could have also integrated phenanthrene and anthracene together, but we chose to treat them separately and apply the default Agile2 integrator for purposes of comparison. Dozens of manual re-integrations each of phenanthrene and anthracene were needed, especially at the lowest levels, to correct the mistakes by the default program when it selected the wrong peak or used an inconsistent baseline. In the case of the other 107 analytes, IS, and QC standard, only spot-checking of integrations was conducted out of curiosity and no manual adjustments were made.

The 5-day validation experiment generated 66,950 data points (ion transition peaks) that would have taken an analyst several days to check that each peak was integrated (or not) appropriately and consistently. Using the summation integration function, the analyst spent only a few hours to set parameters, and all results were generated within a matter of minutes. Not only was human review of results not needed, the consistency of the summation integrations was superior to human capabilities and led to reliable quantitative results (see Supplemental information). Chemical interferences could be observed, especially in dried basil, orange, and black olive, but such interferants complicate peak integrations and worsen quality of results for any integration function.

Different means of post-run data processing batches were considered before settling on the final approach to report results calculated with and without normalization to the IS (see Table 1) using MM calibration standards for the same matrix. Potentially better results could have been obtained by narrowing the summation integration time windows to exactly accommodate the peak widths for each matrix, which were analyzed within hours of each other, but the wider windows that fully covered all peaks for all 325 injections were used in all cases. One drawback of the current software version was that the reported analyte *t*_R_ was the midpoint between the integration start and stop times rather than the actual apex of the peak, and this should be corrected in the future.

### Validation Results

Table 3 lists the compiled results with normalization to the IS for all 94 analytes (including pairs) in the 10 matrices spiked at different levels (*n* = 40 each), as well as overall results for all levels and matrices (*n* = 160). Underlined font emphasizes when overall results yielded 100 % ± 10 % recoveries, and bold text indicates when <70 or >120 % recovery and ≥25 % RSD were obtained. When using the IS, 42 out of 97 analytes gave 90–110 % recoveries and ≤10 % RSD, which is exceptional considering that 230 injections of 10 complex matrices at ultra-trace spiking levels were made without manual re-integrations or instrument maintenance over 5 days sequentially. For comparison, the overall average recoveries and RSD for each analyte without normalization to the IS are also presented in Table 3. Clearly, the IS were needed to provide better precision in the results, and to account for partial retention of the larger PAHs. For nearly all pesticides and most FRs and PCBs, the IS only helped to account for volume fluctuations in the mini-SPE cleanup step. The variability of the elution volume can be observed in Fig. 1, and Table 3 shows how well the IS compensated for these fluctuations from day-to-day and matrix-to-matrix. The differing amounts of water in the different matrices and initial and final extracts also likely contributed substantially to the variations without the IS.

**Table 3:** Average mini-SPE % recoveries (and %RSD) of the 94 analytes (including three pairs) in 10 matrices at four spiking levels (*n* = 40 at each level, and *n* = 160 overall) in the validation study

Nos.	Analyte	10 ng mL^−1^	25 ng mL^−1^	50 ng mL^−1^	100 ng mL^−1^	Overall vs. IS	Overall w/o IS
Pesticides							
1	Acephate	98 (16)	90 (14)	93 (20)	92 (20)	95 (17)	103 (41)
2	Aldrin	91 (12)	89 (10)	91 (11)	93 (8)	90 (11)	93 (24)
3	Atrazine	99 (8)	95 (5)	96 (3)	97 (4)	98 (6)	104 (26)
4	Bifenthrin	93 (8)	91 (5)	93 (9)	93 (9)	93 (9)	99 (30)
5	Carbofuran	102 (11)	97 (9)	98 (9)	101 (11)	101 (11)	106 (34)
6	Carbophenothion	99 (10)	94 (5)	97 (9)	97 (9)	98 (10)	105 (32)
7	Chlorothalonil	95 (16)	87 (14)	88 (16)	90 (17)	91 (16)	97 (31)
8	Chlorpyrifos	96 (8)	93 (4)	95 (4)	97 (4)	96 (6)	101 (25)
9	Coumaphos	94 (10)	91 (8)	92 (12)	91 (11)	93 (11)	101 (30)
10	Cyfluthrin	92 (8)	91 (7)	92 (10)	92 (9)	92 (9)	100 (29)
11	Cypermethrin	95 (14)	88 (8)	92 (13)	91 (11)	93 (13)	100 (37)
12	Cyprodinil	87 (8)	86 (5)	87 (7)	88 (7)	88 (7)	93 (28)
13	Deltamethrin	111 (**50**)	92 (22)	93 (19)	88 (22)	98 (**34**)	119 (59)
14	*o,p′*-DDE	94 (7)	91 (5)	92 (5)	94 (4)	93 (6)	98 (24)
15	*p,p′*-DDE	92 (8)	89 (7)	92 (5)	93 (4)	91 (7)	98 (30)
16	Diazinon	101 (10)	96 (7)	95 (5)	97 (5)	98 (8)	105 (29)
17	Dicrotophos	109 (20)	97 (11)	94 (9)	96 (9)	101 (15)	107 (35)
18	Dimethoate	101 (8)	96 (5)	96 (6)	98 (6)	99 (7)	106 (30)
19	Diphenylamine	102 (15)	97 (8)	98 (8)	100 (6)	100 (11)	106 (25)
20	Endosulfan I	98 (14)	93 (6)	94 (6)	95 (6)	95 (9)	100 (26)
21	Endosulfan II	97 (8)	93 (8)	94 (8)	95 (6)	96 (8)	102 (28)
22	Endosulfan sulfate	96 (18)	95 (9)	96 (11)	97 (8)	98 (13)	104 (31)
23	Esfenvalerate	96 (11)	91 (8)	93 (12)	93 (11)	94 (12)	103 (33)
24	Ethalfluralin	101 (12)	96 (9)	96 (10)	97 (7)	98 (10)	105 (25)
25	Ethoprop	99 (24)	98 (12)	97 (9)	98 (8)	98 (15)	104 (28)
26	Fenpropathrin	91 (**70**)	93 (**34**)	89 (**26**)	89 (17)	92 (**41**)	119 (90)
27	Fipronil	105 (12)	98 (10)	99 (13)	98 (12)	101 (12)	109 (32)
28	Flutriafol	99 (9)	95 (5)	96 (11)	96 (9)	98 (9)	105 (32)
29	Heptachlor	94 (14)	92 (13)	93 (11)	94 (10)	94 (12)	99 (25)
30	Heptachlor epoxide	98 (9)	95 (8)	95 (6)	97 (5)	97 (8)	102 (23)
31	Heptenophos	103 (15)	96 (10)	97 (12)	100 (8)	100 (13)	105 (27)
32	Hexachlorobenzene	83 (20)	87 (10)	89 (11)	90 (11)	86 (14)	88 (26)
33	Imazalil	83 (**21**)	81 (13)	83 (14)	83 (10)	83 (15)	87 (37)
34	Kresoxim-methyl	100 (10)	96 (6)	97 (8)	98 (7)	99 (8)	106 (29)
35	Lindane	100 (11)	97 (7)	97 (7)	99 (6)	99 (9)	104 (23)
36	Methamidophos	97 (16)	91 (15)	92 (16)	95 (16)	96 (16)	103 (35)
37	Methoxychlor	95 (**25**)	92 (23)	94 (**26**)	91 (25)	93 (25)	101 (39)
38	Mirex	83 (9)	82 (10)	85 (12)	85 (12)	83 (11)	88 (33)
39	Myclobutanil	99 (7)	95 (5)	96 (9)	96 (8)	98 (8)	105 (30)
40	*cis*-Nonachlor	96 (9)	91 (6)	92 (6)	94 (6)	94 (7)	101 (28)
41	*trans*-Nonachlor	93 (8)	91 (6)	92 (6)	94 (5)	93 (7)	98 (25)
42	Omethoate	103 (15)	96 (12)	90 (20)	93 (15)	97 (16)	106 (35)
43	*o*-Phenylphenol	102 (19)	97 (11)	98 (11)	100 (8)	100 (14)	104 (30)
44	Penconazole	94 (8)	92 (4)	93 (8)	94 (7)	94 (7)	101 (29)
45	Pentachlorothioanisole	93 (15)	92 (10)	94 (11)	96 (9)	93 (11)	96 (22)
46	Permethrin	92 (16)	92 (7)	93 (10)	92 (8)	93 (11)	101 (36)
47	Piperonyl butoxide	96 (8)	92 (6)	94 (10)	93 (9)	95 (9)	103 (31)
48	Propargite	101 (15)	94 (9)	95 (14)	97 (14)	97 (16)	103 (30)
49	Pyridaben	93 (8)	89 (6)	91 (11)	91 (9)	92 (10)	100 (31)
50	Pyriproxyfen	93 (8)	91 (6)	92 (11)	92 (9)	93 (9)	102 (31)
51	Tebuconazole	93 (14)	87 (9)	90 (13)	89 (10)	91 (12)	100 (34)
52	Tetraconazole	99 (7)	97 (5)	97 (7)	98 (7)	99 (7)	105 (27)
53	Thiabendazole	**63** (**31**)	**64** (25)	**69** (**26**)	70 (25)	**67** (**27**)	71 (41)
54	Tribufos	95 (11)	88 (7)	91 (11)	90 (8)	92 (10)	99 (32)
FRs							
1	BDE 183	101 (**36**)	100 (18)	100 (13)	101 (10)	100 (21)	76 (37)
2	Dechlorane plus (syn + anti)	99 (12)	99 (14)	99 (11)	100 (10)	98 (11)	80 (32)
3	PBB 153	103 (11)	101 (10)	103 (9)	105 (9)	102 (10)	81 (31)
4	PBEB	**63** (20)	**63** (11)	**67** (10)	**67** (15)	**64** (15)	**67** (33)
5	PBT	92 (11)	91 (9)	93 (10)	96 (12)	92 (11)	72 (29)
6	TBB	86 (10)	85 (7)	87 (6)	87 (8)	86 (8)	**69** (33)
7	TBCO	102 (**43**)	93 (**28**)	91 (24)	93 (21)	96 (**30**)	104 (43)
8	TBECH	100 (25)	94 (17)	96 (16)	95 (12)	97 (18)	104 (32)
9	TBNPA	106 (**26**)	98 (14)	98 (16)	100 (11)	101 (18)	103 (34)
10	TBX	70 (12)	69 (8)	73 (7)	74 (10)	70 (11)	73 (26)
11	TCEP	101 (5)	97 (4)	96 (3)	98 (5)	99 (5)	105 (28)
12	TCPP	100 (7)	96 (4)	97 (3)	98 (4)	99 (6)	105 (28)
13	TDCPP	101 (9)	97 (7)	98 (11)	98 (10)	99 (10)	107 (32)
14	Triphenylphosphate	90 (**39**)	96 (**27**)	98 (25)	98 (23)	96 (**28**)	105 (46)
PAHs							
1	Acenaphthene	100 (16)	99 (6)	100 (5)	101 (5)	100 (9)	100 (27)
2	Acenaphthalene	97 (6)	96 (5)	97 (3)	98 (3)	97 (5)	99 (22)
3	Anthracene	100 (11)	92 (5)	95 (5)	96 (4)	96 (8)	85 (27)
4	Benz(a)anthracene + Chrysene	**69** (13)	**66** (12)	**67** (13)	**68** (13)	**68** (13)	**40** (44)
5	Benzo(a)pyrene	109 (9)	97 (6)	95 (3)	97 (4)	100 (8)	**22** (53)
6	Benzo(b + k)fluoranthene	**123** (6)	115 (5)	113 (3)	115 (3)	117 (6)	**27** (48)
7	Benzo(g,h,i)perylene	120 (15)	103 (8)	100 (4)	102 (4)	106 (12)	**14** (64)
8	Dibenz(ah)anthracene	100 (13)	84 (8)	82 (5)	82 (5)	87 (13)	**19** (58)
9	Fluoranthene	104 (5)	97 (4)	96 (3)	97 (3)	99 (5)	**63** (32)
10	Fluorene	115 (17)	116 (10)	113 (7)	114 (8)	114 (12)	98 (24)
11	Indeno(1,2,3-c,d)pyrene	117 (14)	109 (12)	108 (8)	114 (12)	113 (12)	**17** (61)
12	Naphthalene	**128** (**95**)	118 (**78**)	117 (**29**)	108 (**28**)	115 (**63**)	114 (71)
13	Phenanthrene	114 (16)	101 (5)	99 (4)	99 (3)	104 (11)	89 (30)
14	Pyrene	108 (6)	99 (5)	97 (2)	98 (3)	101 (6)	**57** (36)
PCBs							
1	PCB 77	97 (17)	96 (7)	97 (8)	98 (9)	97 (11)	85 (29)
2	PCB 81	100 (8)	96 (8)	97 (6)	99 (7)	98 (8)	85 (27)
3	PCB 105	106 (7)	104 (8)	107 (6)	108 (6)	106 (7)	91 (26)
4	PCB 114	106 (5)	104 (7)	106 (7)	108 (8)	106 (7)	90 (26)
5	PCB 118 + 123	103 (8)	102 (7)	106 (7)	107 (7)	104 (8)	89 (28)
6	PCB 126	88 (12)	85 (9)	87 (11)	88 (11)	86 (11)	75 (29)
7	PCB 156 + 157	100 (8)	98 (8)	100 (9)	101 (9)	99 (9)	85 (26)
8	PCB 167	98 (7)	96 (8)	100 (8)	100 (9)	97 (9)	83 (28)
9	PCB 169	75 (19)	72 (13)	75 (13)	74 (14)	74 (15)	**63** (30)
10	PCB 170	98 (8)	97 (9)	99 (9)	100 (10)	98 (9)	83 (29)
11	PCB 180	100 (8)	95 (9)	99 (9)	98 (11)	96 (10)	82 (28)
12	PCB 189	92 (16)	92 (12)	91 (16)	93 (15)	91 (15)	76 (25)

Bold text indicates recovery <70 % or RSD >25 %

Underlining emphasizes overall recoveries of 90–110 % and RSD of 10 % or less

When using the IS, only three analytes (PBEB, benz(a)anthracene + chrysene, and thiabendazole) had <70 % overall average recoveries (64, 68, and 67 %, respectively) and only six analytes (naphthalene, TPP, TBCO, fenpropathrin, deltamethrin, and thiabendazole) had overall RSDs >25 %. TPP and naphthalene were ubiquitous in all of the reagent blanks at varying concentrations averaging 7 and 75 ng mL^−1^, respectively, and similar levels in the final matrix extracts. Analytical conditions for fenpropathrin were not optimal, and an interferant averaging 18 ng g^−1^ equivalent response adversely affected its results. Deltamethrin is known to degrade in the injection inlet [20], which was the main cause of its more variable results, which was also the case for methoxychlor to a lesser extent. Last, thiabendazole and TBCO (and TBECH) gave very broad chromatographic peaks. Despite the inconsistent results for those six analytes, the chromatographic peak shapes and results were very good for other notoriously difficult analytes in GC, such as methamidophos, acephate, omethoate, dimethoate, carbofuran, chlorothalonil, imazalil, and myclobutanil (see Supplemental information). In fact, dimethoate was among those analytes that gave the best results, and cyfluthrin was another analyte that gave surprisingly high quality results.

Despite the much greater variability, the overall recoveries without normalization to the IS were the same as when using the IS except PBEB and PCB 169 were 67 and 63 %, respectively, and 8 PAHs had 14–63 % uncompensated recoveries. As stated earlier, the 1 mg CarbonX was probably the cause for the lower recoveries of structurally (co-)planar analytes, but the isotopically labeled IS worked well to improve accuracy in all cases, albeit an ideal IS was not obtained for benz(a)anthracene + chrysene or benzo(b + k)fluoranthene.

In addition to its high quality of results, the automated mini-SPE + LPGC-MS/MS method provided excellent ruggedness in the analysis of all 325 samples. Due to the clean extracts, small injection volumes, mega-bore analytical column, and use of APs, the same injection liner and septum was used for all 325 injections in 10 matrices over the course of 5 days. A picture of the used liner and septum appears in Supplemental information. Even the fatty salmon matrix, which was analyzed last, showed very good peak shapes and perfectly linear calibration with the same high quality results as the other matrices (see Supplemental information).

### Matrix Effects (MEs)

**Fig. 8:**
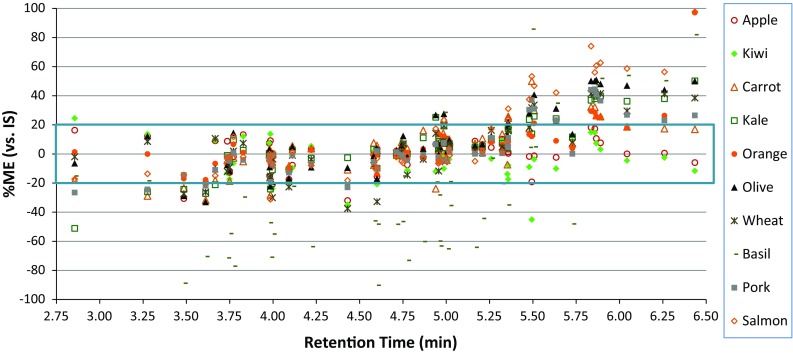
Matrix effects (MEs) of the 54 pesticides plotted vs. *t*_R_ in LPGC-MS/MS using APs after automated mini-SPE cleanup of QuEChERS extracts of 10 matrixes

MEs were measured both with and without normalization of integrated peak areas to the IS. As in the case of recoveries, the IS helped to greatly reduce imprecision in the measurements. As shown in Supplemental information, the PCBs, PAHs, and nearly all FRs did not undergo MEs (<|±20 %|), mainly due to their nonpolar nature (relatively polar analytes are more susceptible to MEs). Figure 8 shows MEs in the case of pesticides in each matrix plotted vs. increasing *t*_R_. When MEs <|±20 %| occur (shown by the box in the figure), then RO standards can be used to yield similar results as MM calibration standards. Despite the common practice in residue analysis to use MM standards, they require storage of many blank matrices and take more time for sample preparation, plus the extra materials, labor, and costs involved, than simply preparing RO calibration standards. Use of MM standards also causes more matrixes to be introduced to the instrument during analytical batches, as well as complications to match given matrices when different commodities are analyzed in the same sequence. Calibration using RO standards is more advantageous in practice, provided that MEs do not lead to unacceptable accuracy.

Unlike LC–MS/MS in which ion suppression occurs due to MEs [26], signal enhancement commonly occurs to susceptible analytes in GC analysis [27]. However, as shown in Fig. 8, basil induced a severe response diminishment effect, presumably due to the sheer amount of co-extractives from the dried herb even after mini-SPE cleanup. Herbs and spices are unique matrices that require MM calibration even after extensive cleanup in any GC-MS or LC–MS method. With few exceptions between 2.5 and 5.5 min *t*_R_, MEs for the analytes fell within |±20 %| (see Fig. 8). As described previously [19], the APs can overcome MEs for the analytes with *t*_R_ less than about permethrin, but a good AP for the late-eluting pyrethroids has not been found yet. The current mixture of APs (and their breakdown products) is too volatile to co-elute with the late-eluting analytes, which is shown clearly in Fig. 8. In this study, 10 pesticides underwent >20 % MEs for all matrices except the least complex fruits and vegetables (apple, kiwi, and carrot). These analytes consist of coumaphos, pyridaben, methoxychlor, and the pyrethroids: bifenthrin, fenpropathrin, permethrin, cyfluthrin, cypermethrin, esfenvalerate, and deltamethrin). Similarly, late-eluting PCBs, PAHs, and FRs yielded insignificant MEs, especially when using IS with *t*_R_ near the analytes. Despite the nonpolar nature of these PCBs, PAHs, and FRs, MEs were observed to a greater extent without normalization to the IS, which leads us to hypothesize that use of an appropriate IS for pyrethroids may solve this problem with excessive MEs.

## Conclusions

The automated mini-SPE cleanup coupled with LPGC-MS/MS analysis not only achieved high quality results for diverse type of analytes and foods, the approach also enabled reliable, high-throughput operations without much labor or instrument maintenance. The use of a state-of-the-art GC-MS/MS instrument permitted injection of merely 1 mg sample equivalent while still achieving <5 ng g^−1^ LOQs for all analytes (LOQ < 0.5 ng g^−1^ for most analytes). The use of APs also helped lead to very consistent *t*_R_ and peak shape for the analytes, even after hundreds of sample injections, which enabled use of summation function integration to eliminate human review of chromatographic peak integrations. This data processing approach saved many hours of analyst time, which is usually the rate limiting in real-world practice.

Using the final method in unattended instrument operation, two analysts conducted the full validation study involving 235 samples in 5 days. One of the analysts performed initial QuEChERS extractions, and because we do not have barcode reading, the other analyst’s time in the lab was spent labeling vials and entering sample names into long sequences. In the final method, the automated mini-SPE step took 8 min per sample, and the LPGC-MS/MS step was 13 min including oven re-equilibration. In the future, we intend to substantially reduce the time needed for both operations.

Last, some incurred analytes were quantified and identified >10 ng g^−1^ in the tested commodities: imazalil (1243 ng g^−1^) and thiabendazole (640 ng g^−1^) were detected in the oranges, *p,p′*-DDE (56 ng g^−1^) was determined in kale, and diphenylamine (17 ng g^−1^) and thiabendazole (16 ng g^−1^) were detected in the Gala apple. A few other pesticides were identified between the 5 ng g^−1^ lowest calibrated level and 10 ng g^−1^ reporting level. The practical application of this automated approach has been demonstrated to meet real-world monitoring needs.

## Electronic supplementary material

Below is the link to the electronic supplementary material.

Supplementary material 1 (PDF 4212 kb)
