# Supplementary material for: Automated Mini-Column Solid-Phase Extraction Cleanup for High-Throughput Analysis of Chemical Contaminants in Foods by Low-Pressure Gas Chromatography—Tandem Mass Spectrometry
Source: Chromatographia. 2016 Jun 13;79(17):1113–30. doi: 10.1007/s10337-016-3116-y (PMC4995235; doi:10.1007/s10337-016-3116-y)

Supplemental information for

“Automated mini-column solid-phase extraction cleanup for rapid analysis of chemical contaminants in foods by low-pressure gas chromatography – tandem mass spectrometry”

by Steven J. Lehotay, Lijun Han, and Yelena Sapozhnikova

USDA Agricultural Research Service
Eastern Regional Research Center
Wyndmoor, Pennsylvania; USA

Contact: Steven.Lehotay@ars.usda.gov

Automated Mini-SPE of QuEChERS **Reagent Blank** Extracts
GC-MS Full Scan *m/z* 100-500, 1 µL injection including **APs**

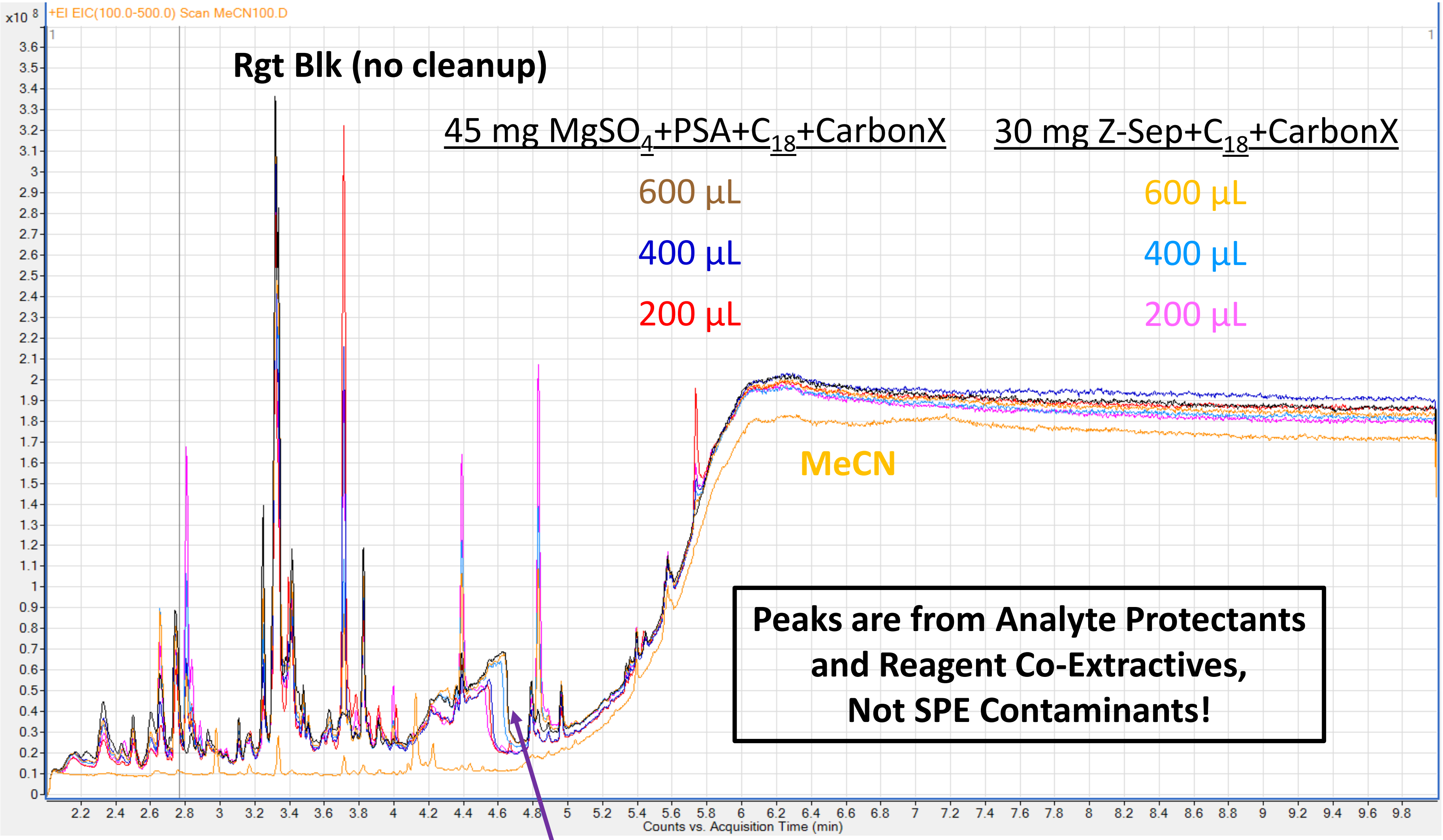

Water in extracts when $MgSO_4$ not included in SPE yields worse cleanup

30 mg C_{18}+Z-Sep+CarbonX Mini-SPE of QuEChERS **Kale** Extracts
GC-MS Full Scan *m/z* 100-500, 1 µL injection including APs

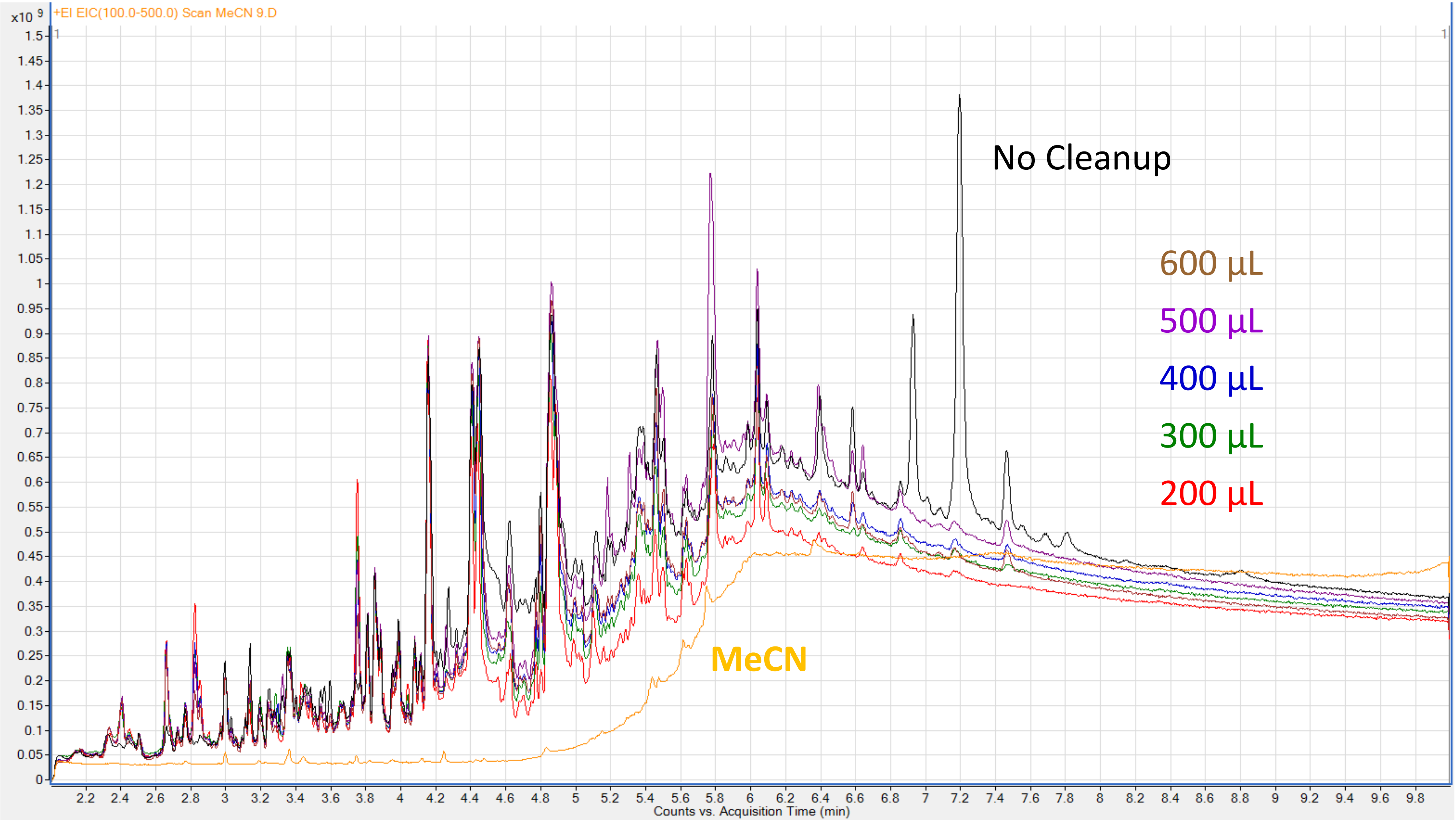

Conclusion: Not much cleanup, but 200-300 µL is ok

30 mg C_{18}+Z-Sep+CarbonX Mini-SPE of QuEChERS **Salmon** Extracts
GC-MS Full Scan *m/z* 100-500, 1 µL injection including APs

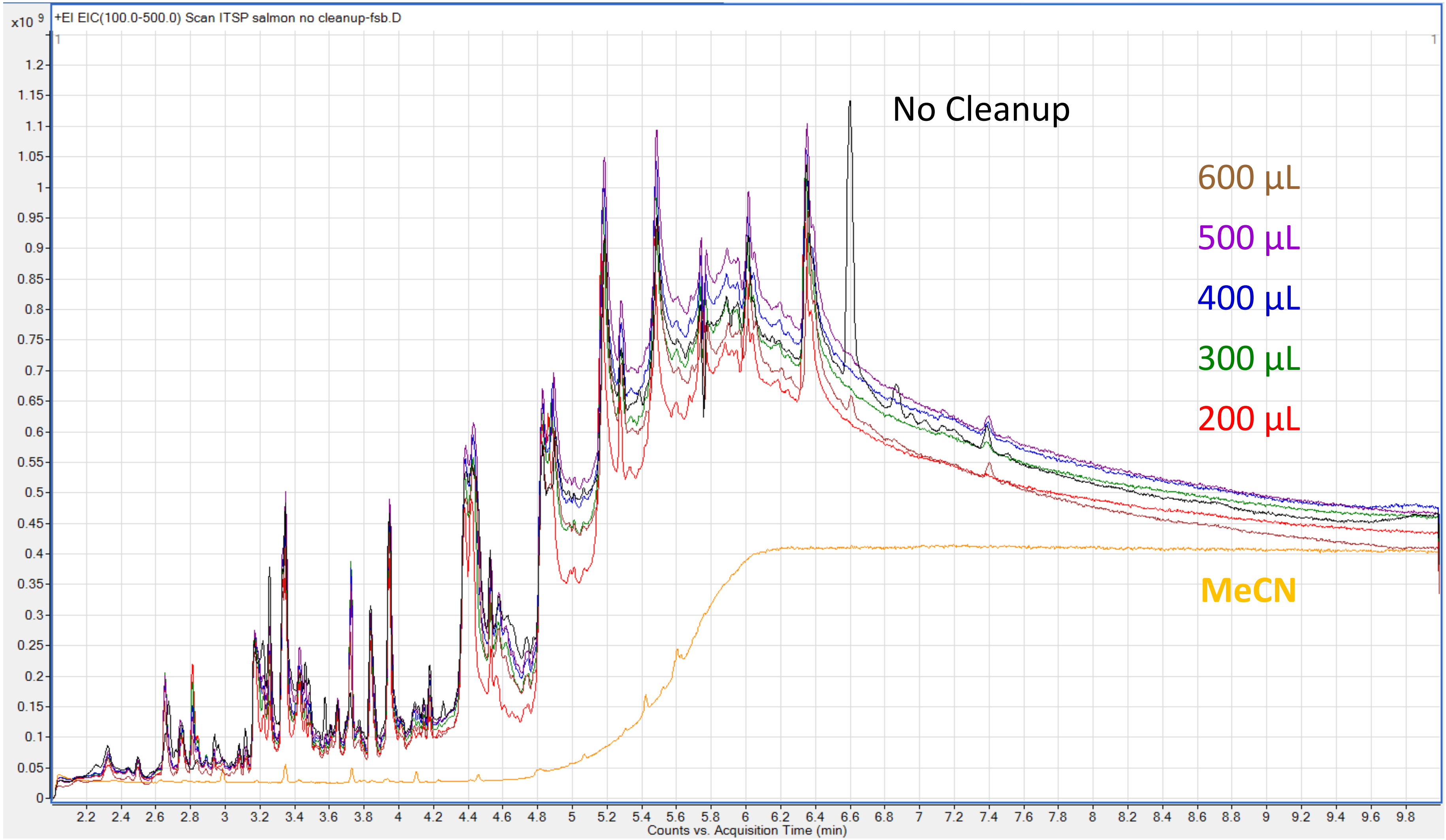

Conclusion: Not much cleanup

30 mg C_{18}+Z-Sep+CarbonX Mini-SPE of QuEChERS Pork Extracts
GC-MS Full Scan *m/z* 100-500, 1 µL injection including APs

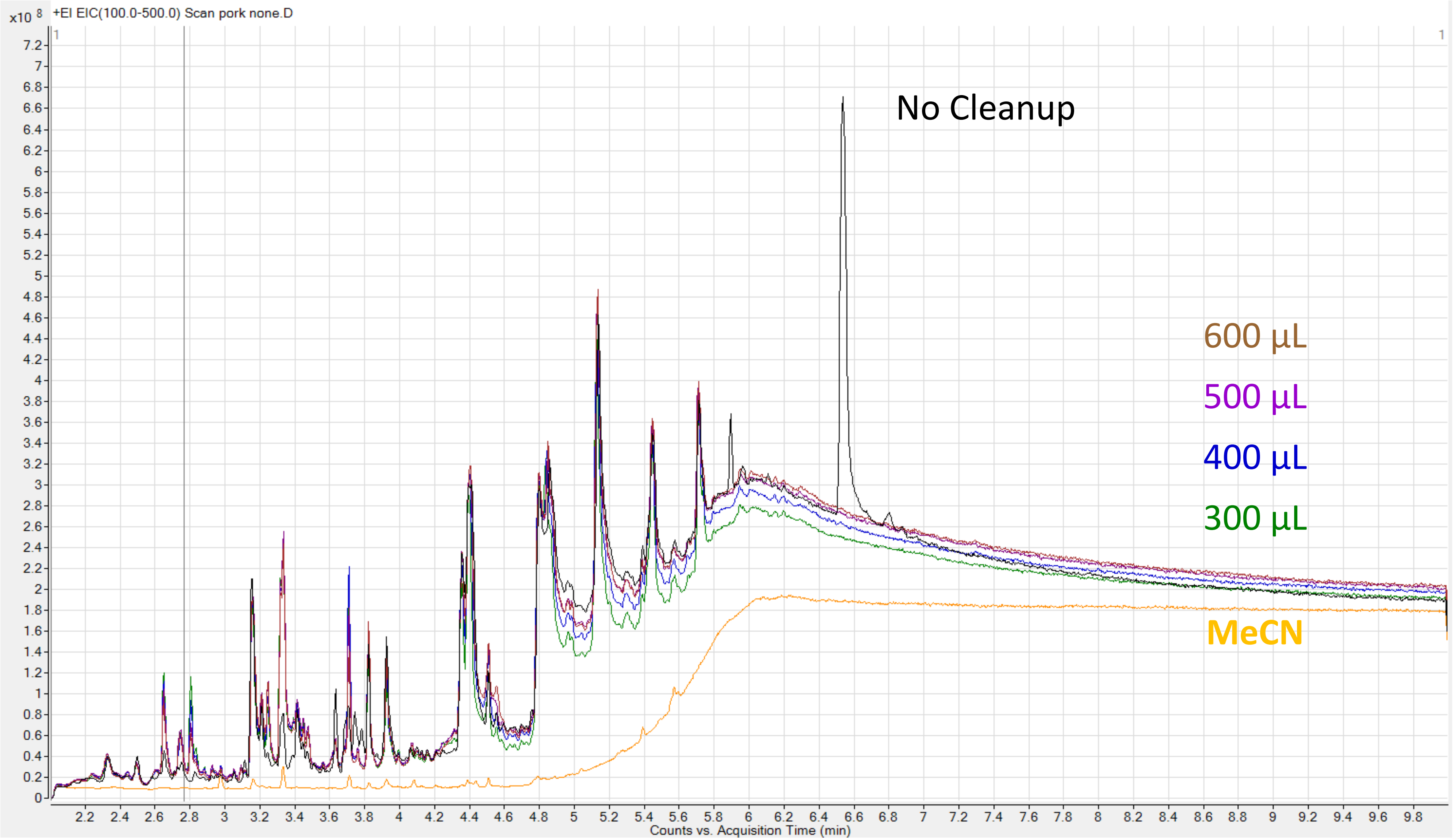

Conclusion: Not much cleanup

30 mg C_{18}+Z-Sep+CarbonX Mini-SPE of QuEChERS **Avocado** Extracts

GC-MS Full Scan *m/z* 100-500, 1 µL injection including APs

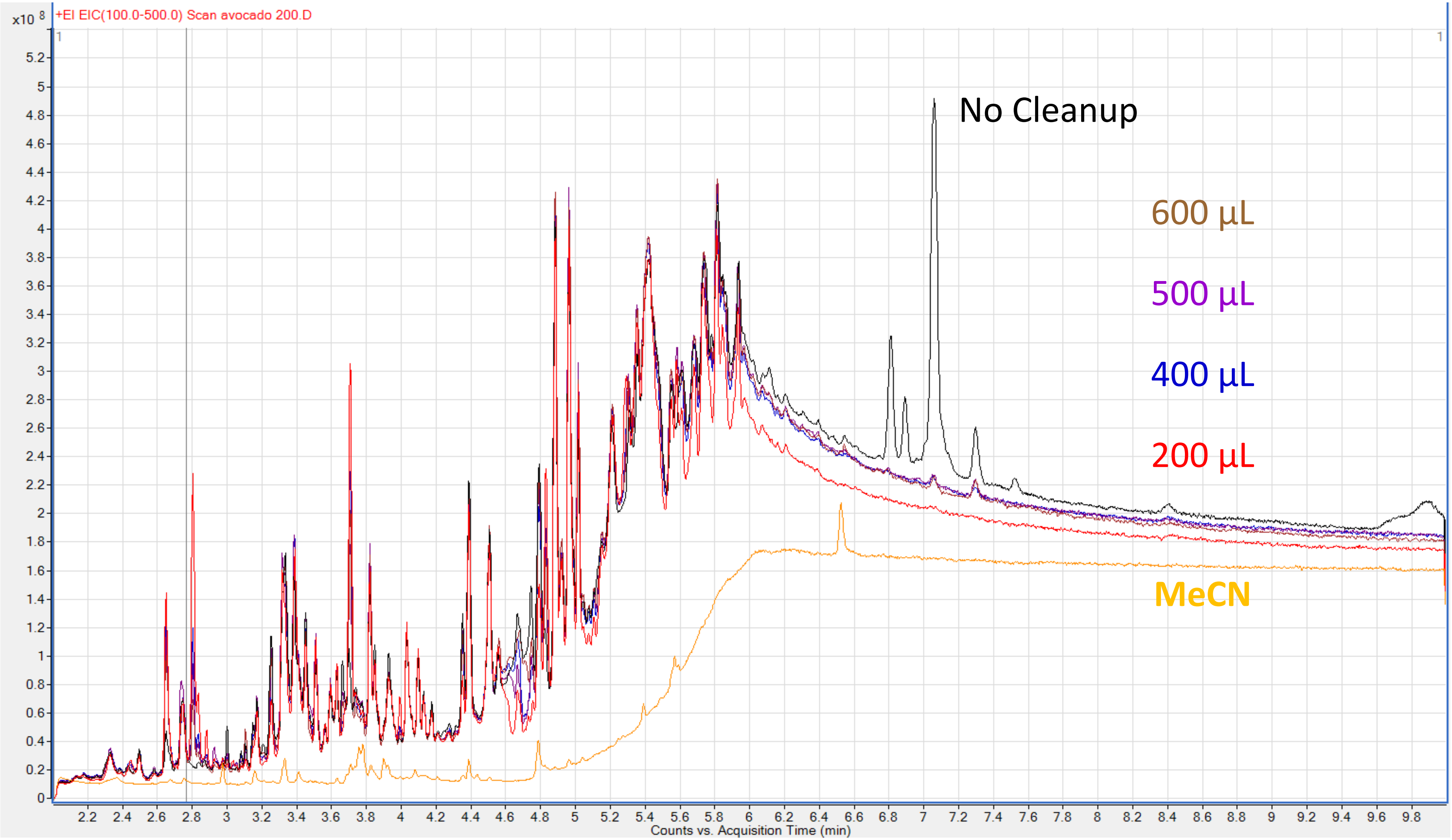

Conclusion: Not much cleanup

30 mg C_{18}+Z-Sep+CarbonX Mini-SPE of QuEChERS **Kale** Extracts

UV/Vis Absorbance Results

Spectrum of Extract before Cleanup

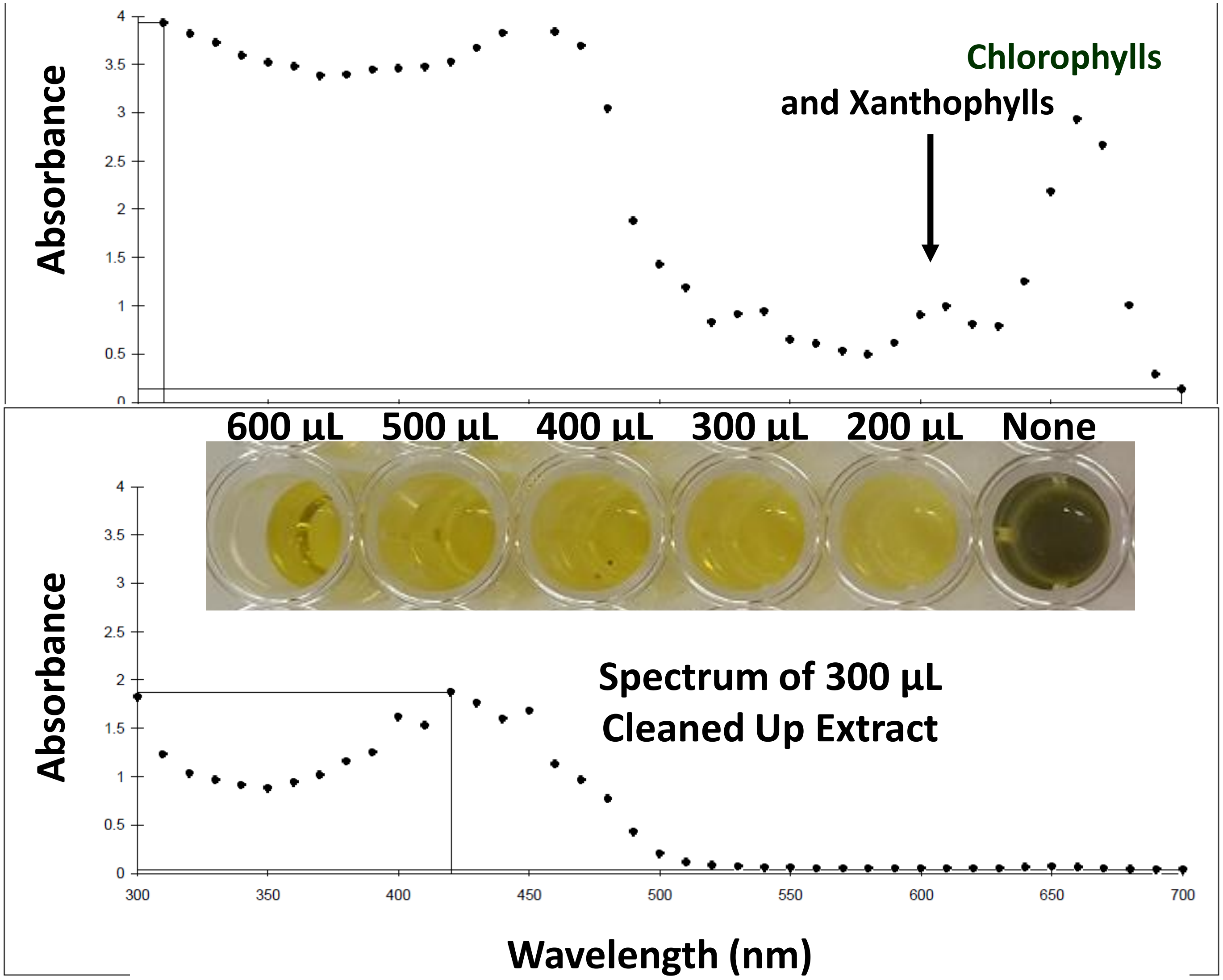

30 mg C_{18}+Z-Sep+CarbonX Mini-SPE of QuEChERS **Salmon** Extracts

UV/Vis Absorbance Results

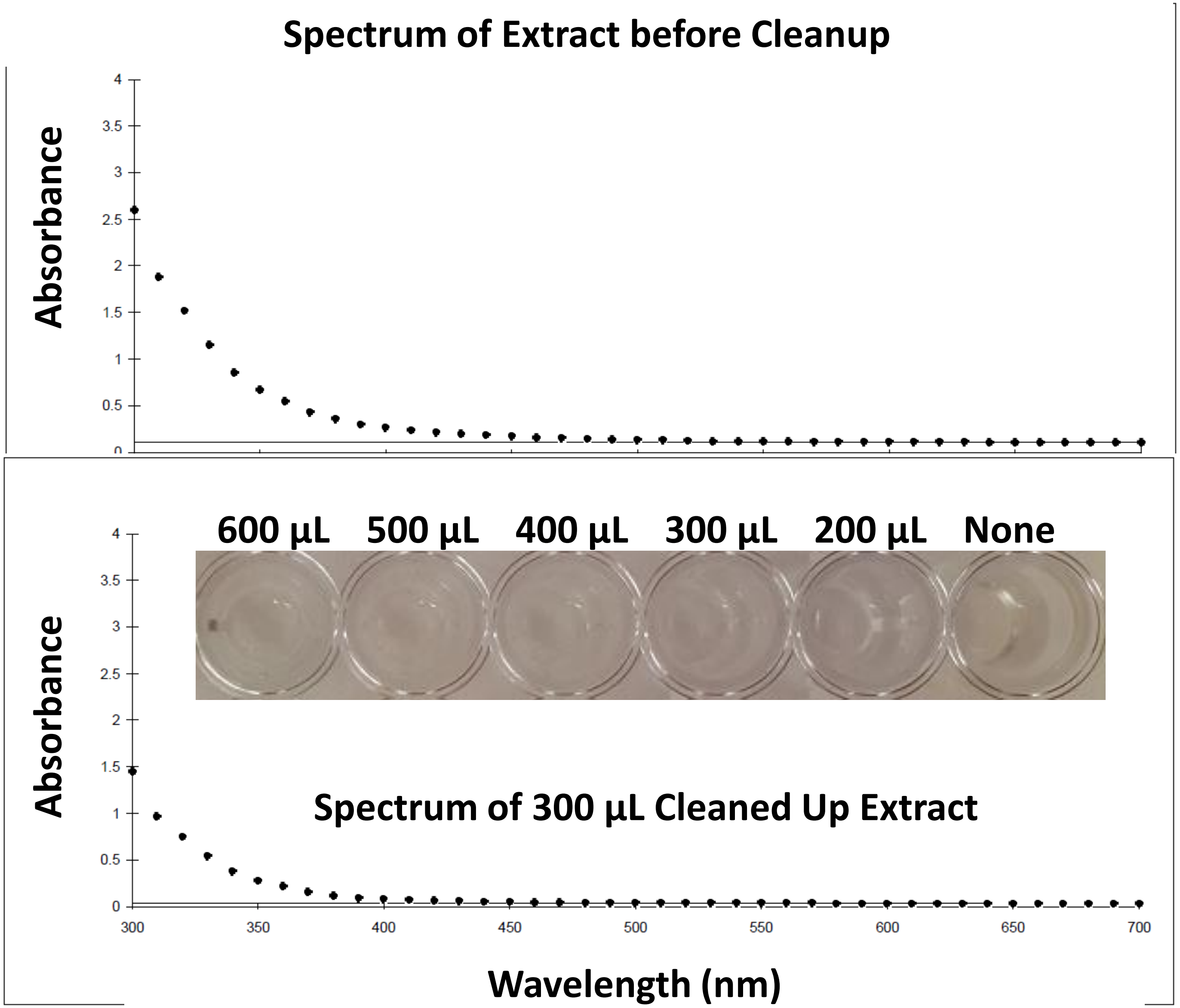

45 mg $MgSO_4$+C_{18}+PSA+CarbonX Mini-SPE of Salmon Extracts

UV/Vis Absorbance Results

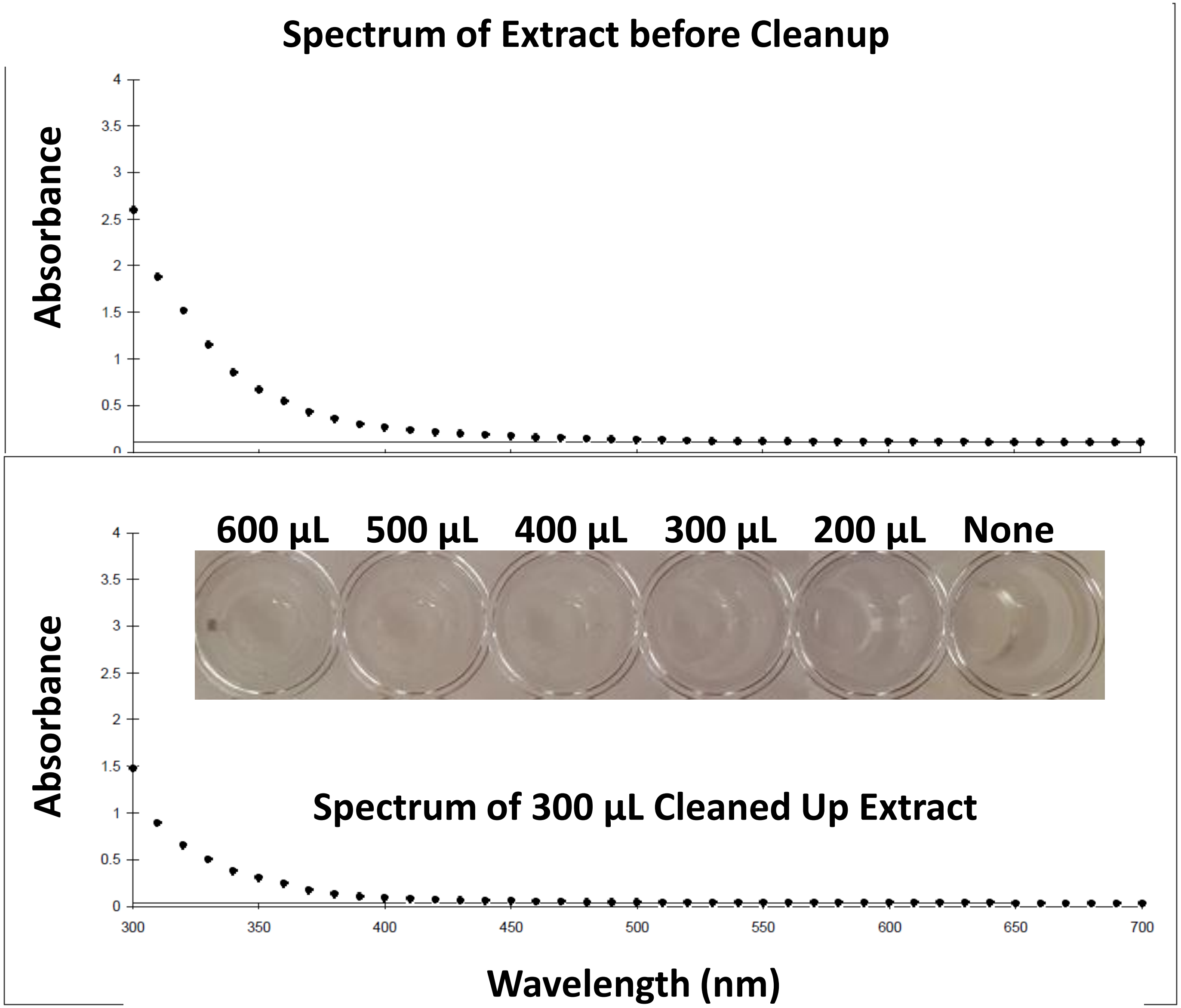

30 mg C_{18}+Z-Sep+CarbonX Mini-SPE of QuEChERS Pork Extracts

UV/Vis Absorbance Results

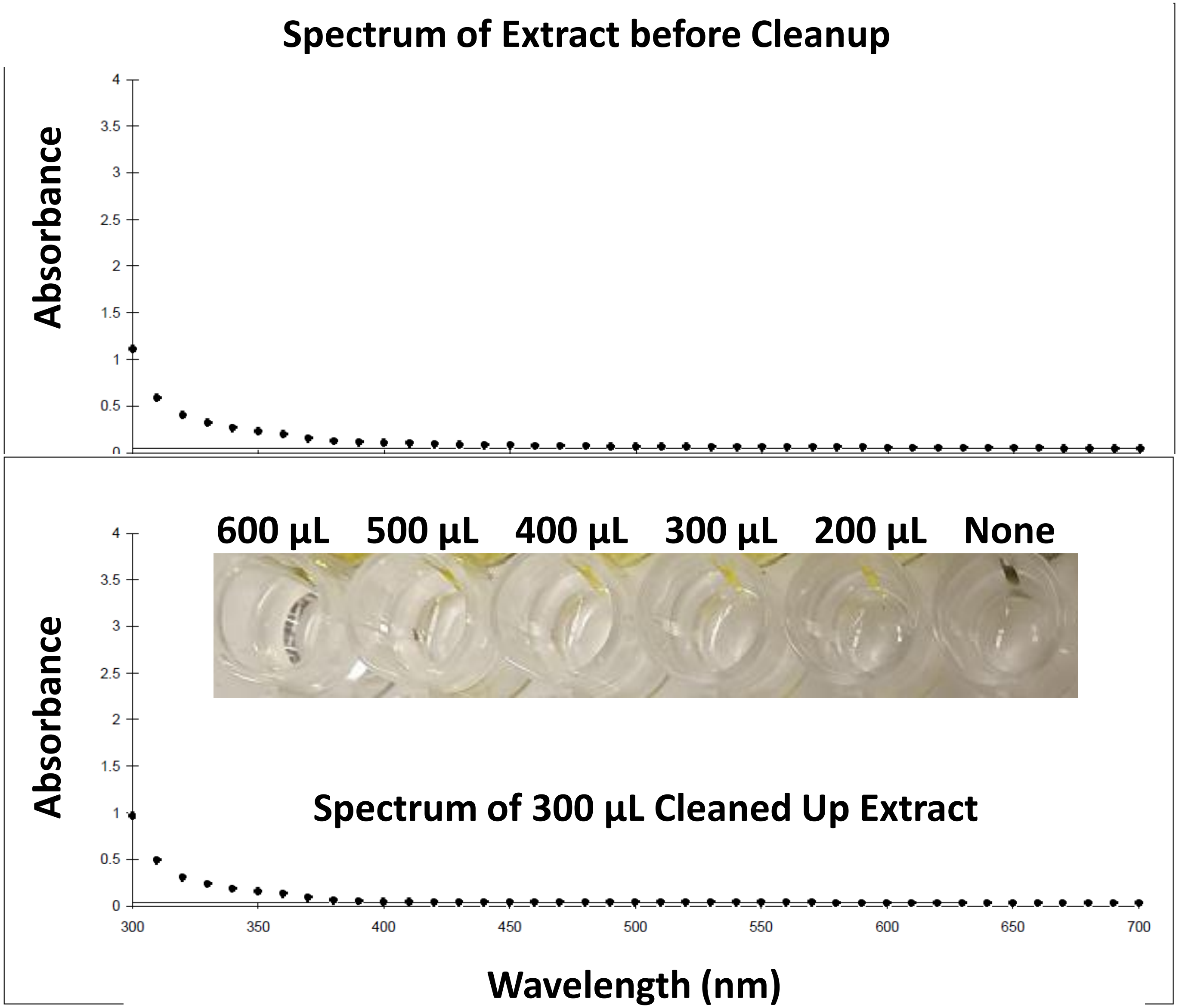

45 mg $MgSO_4$+C_{18}+PSA+CarbonX Mini-SPE of QuEChERS **Pork** Extracts

UV/Vis Absorbance Results

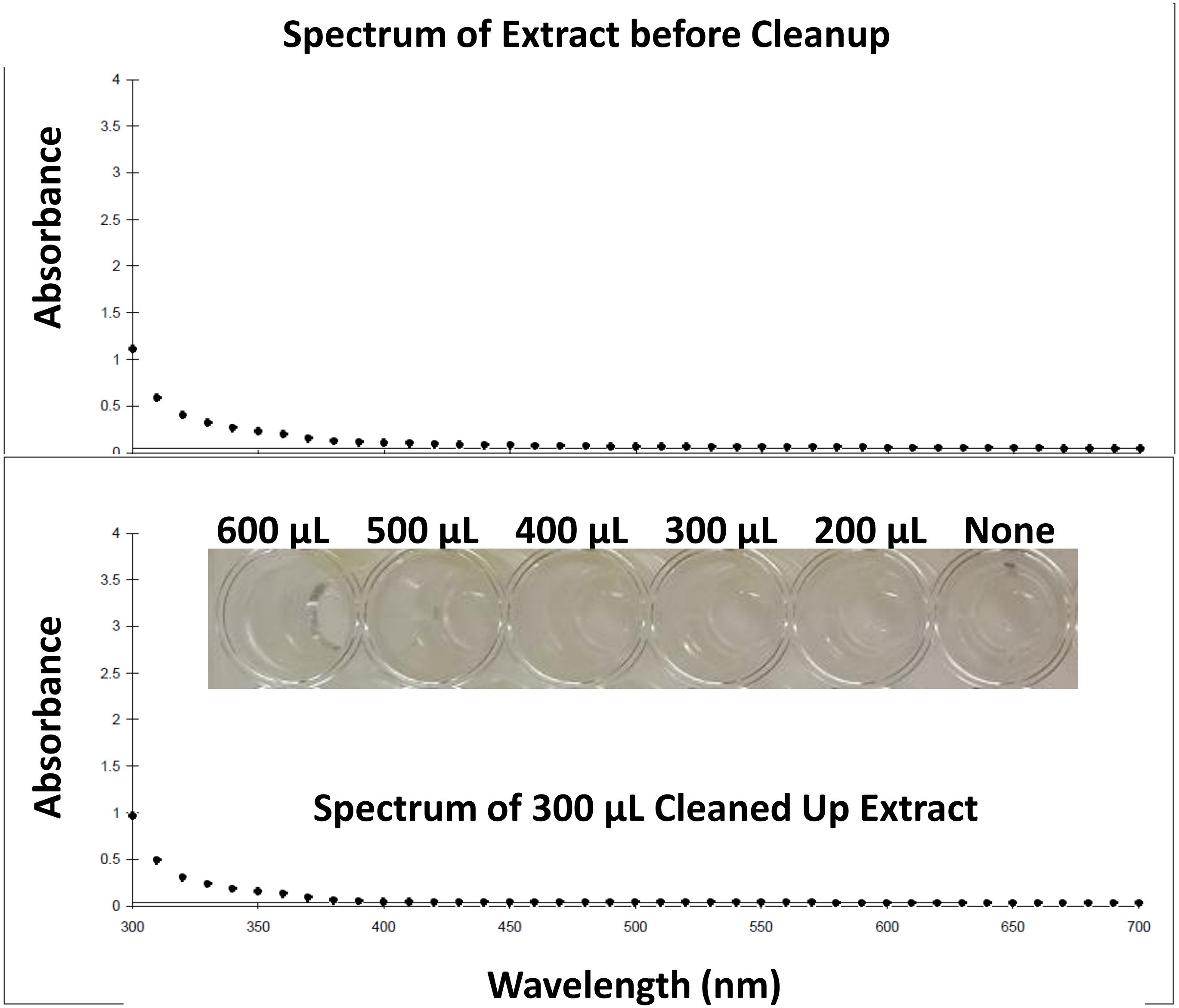

30 mg C_{18}+Z-Sep+CarbonX Mini-SPE of QuEChERS **Avocado** Extracts

UV/Vis Absorbance Results

Spectrum of Extract before Cleanup

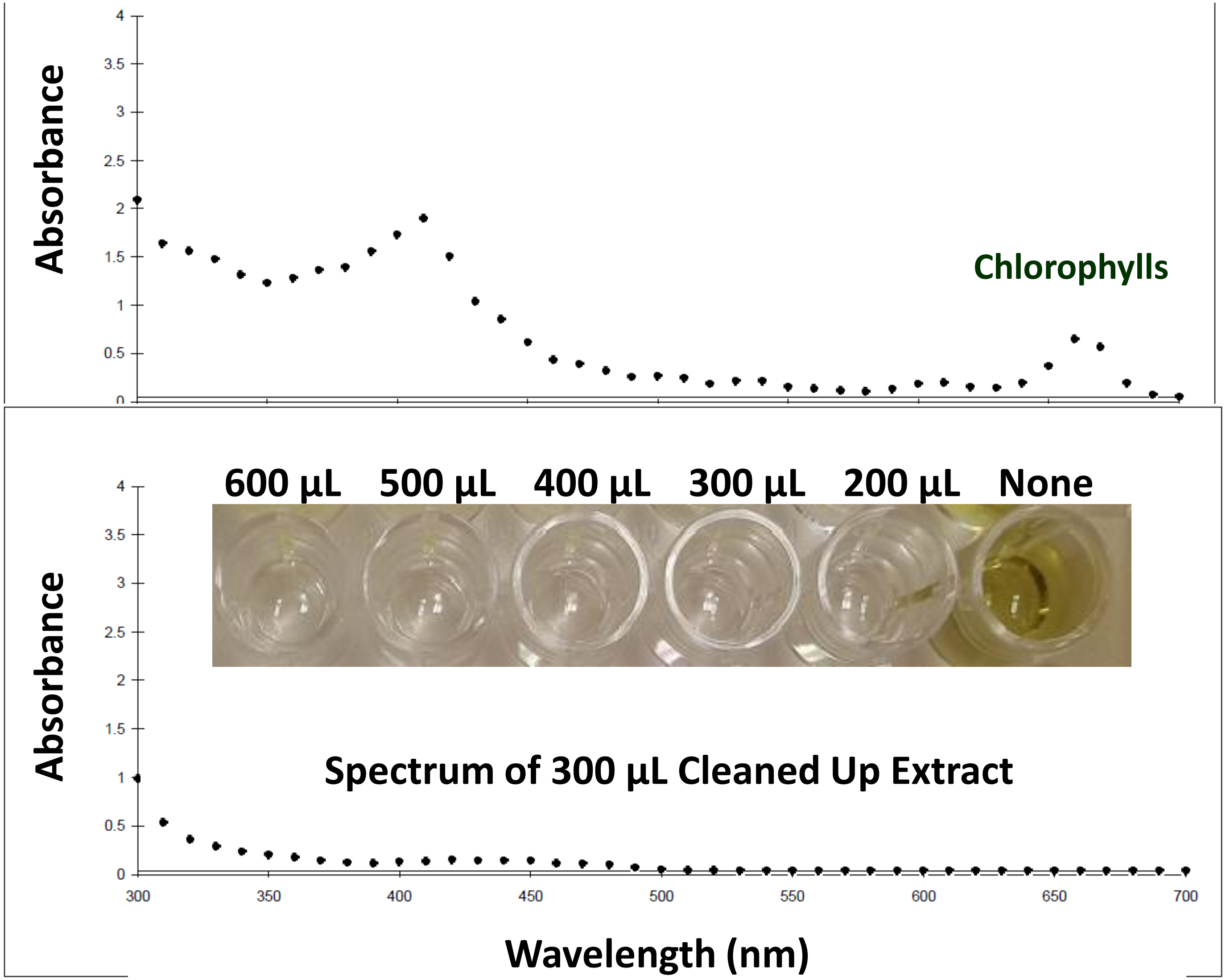

45 mg $MgSO_4$+C_{18}+PSA+CarbonX Mini-SPE of **Avocado** Extracts
UV/Vis Absorbance Results

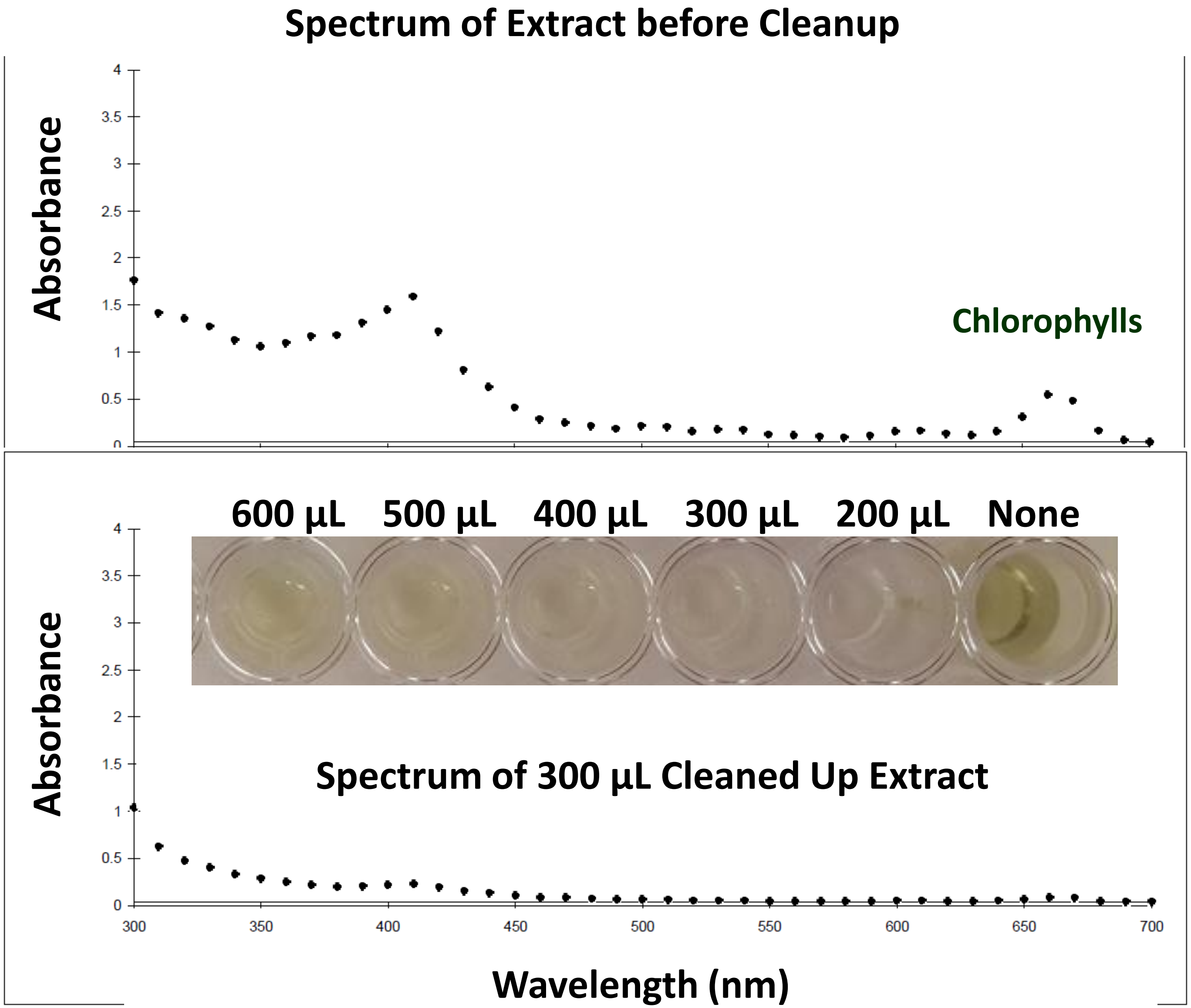

Comparison of Mini-Cartridges to Remove Chlorophyll

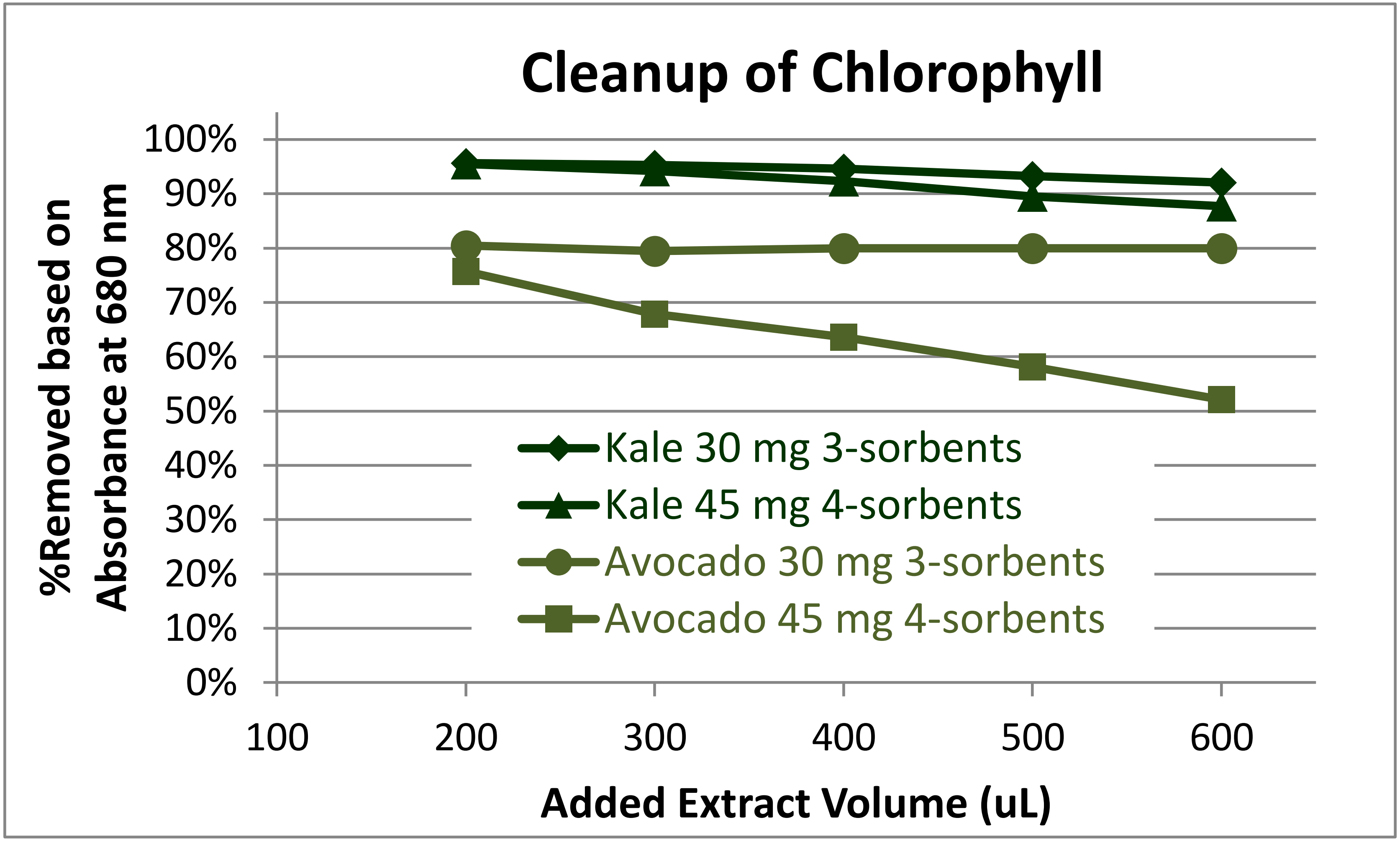

30 mg C_{18}+Z-Sep+CarbonX Mini-SPE of QuEChERS **Kale** Extracts

Chlorophyll Removal and HCB Results vs. Extract Vol. Added

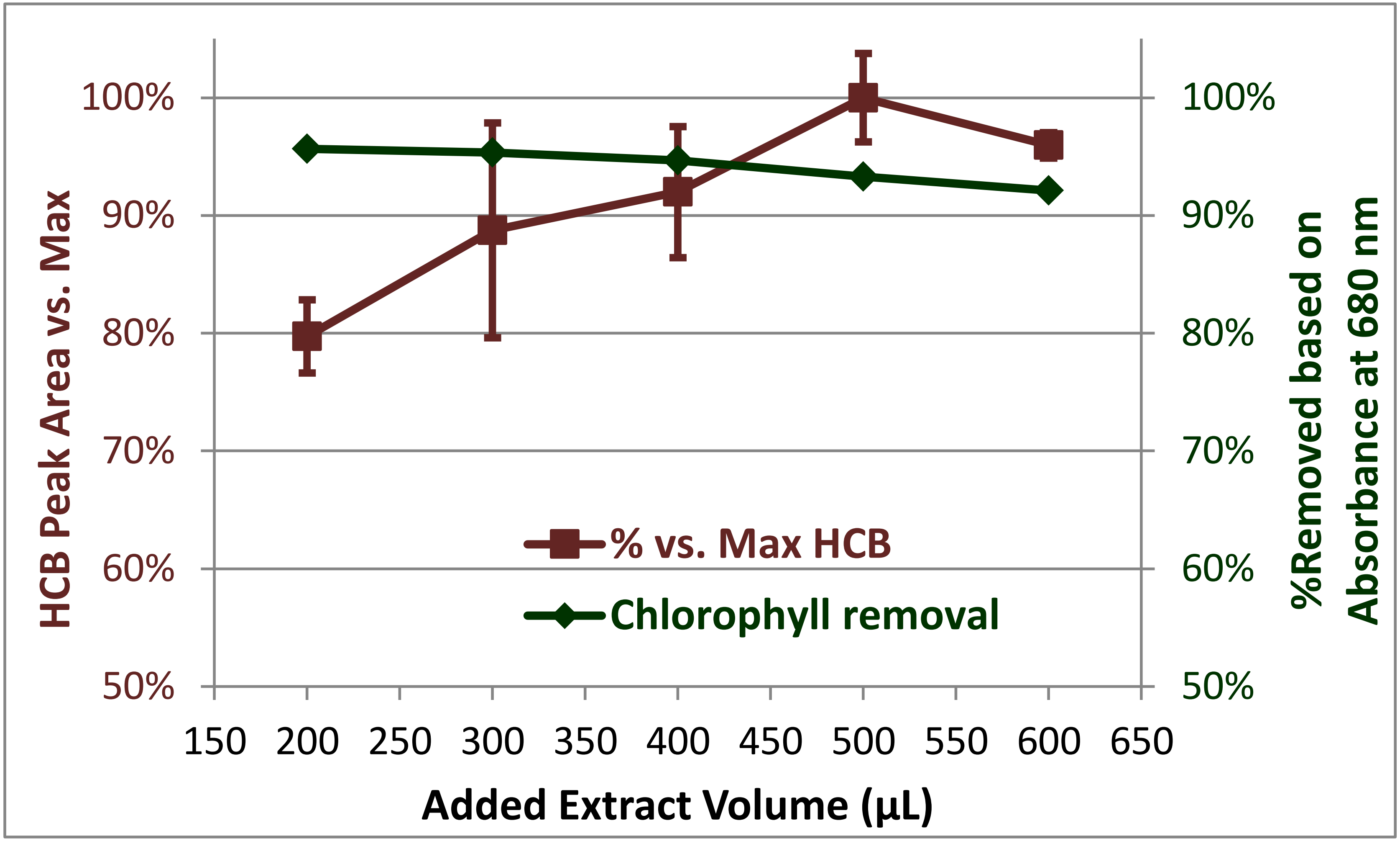

Conclusion: 200 µL extract needed for 80% HCB elution

30 mg C_{18}+Z-Sep+CarbonX Mini-SPE of QuEChERS Extracts

Recovery vs. Extract Vol. Added

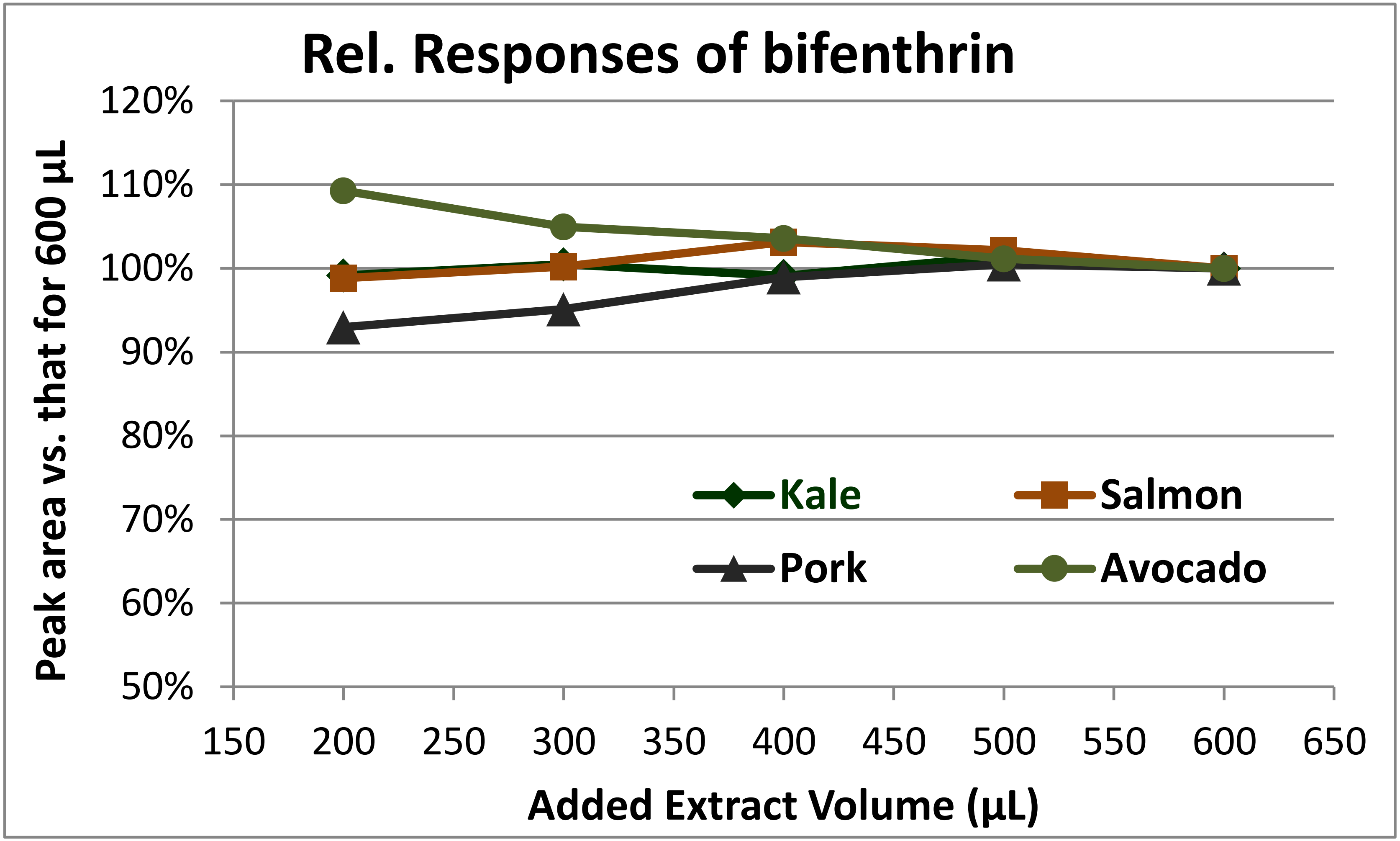

Conclusion: most analytes were not retained by the sorbents

30 mg C_{18}+Z-Sep+CarbonX Mini-SPE of QuEChERS Extracts

<u>Recovery vs. Extract Vol. Added</u>

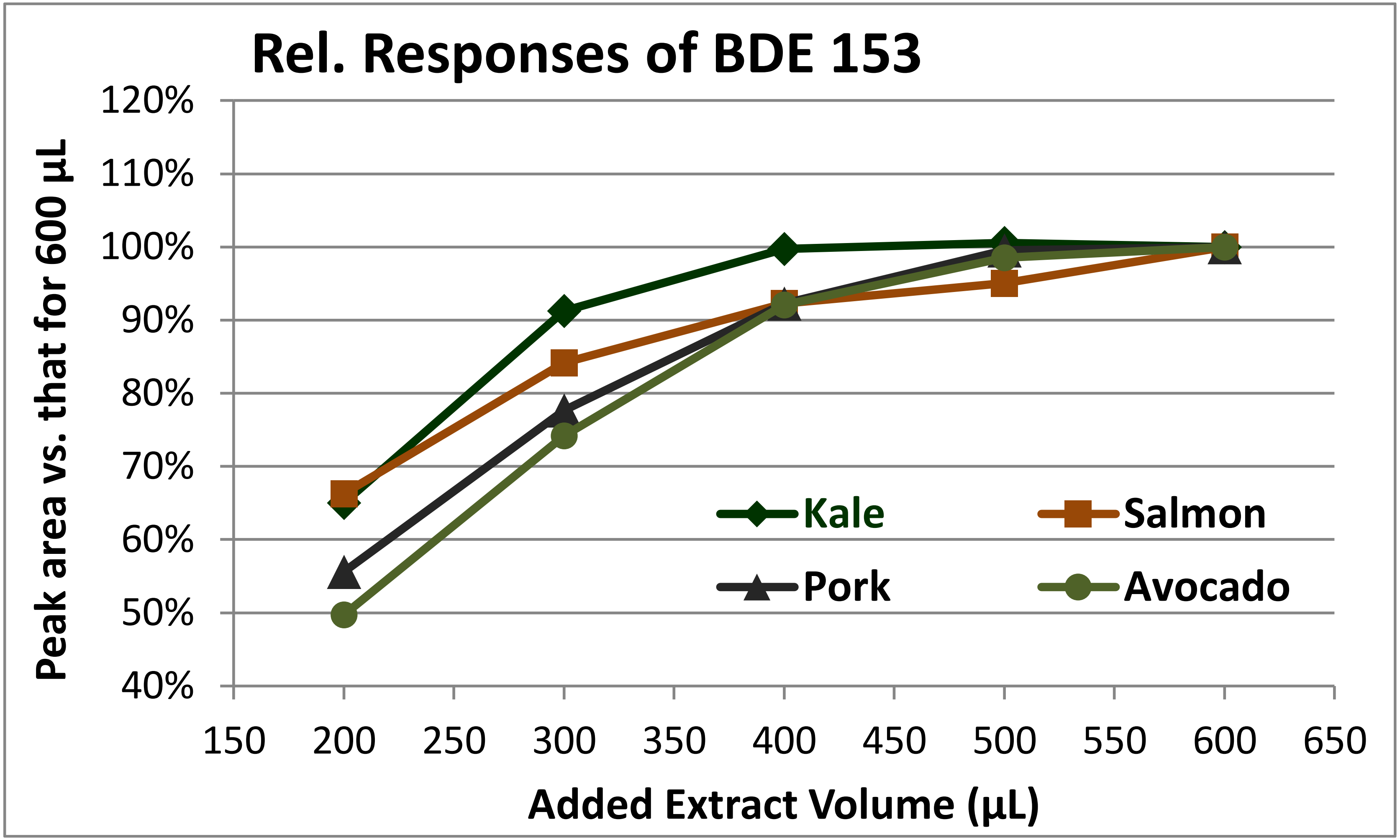

Conclusion: 300 µL extract needed for >70% elution of PBDEs

45 mg $MgSO_4$+C_{18}+PSA+CarbonX ITSP of QuEChERS Extracts

Recovery vs. Extract Vol. Added

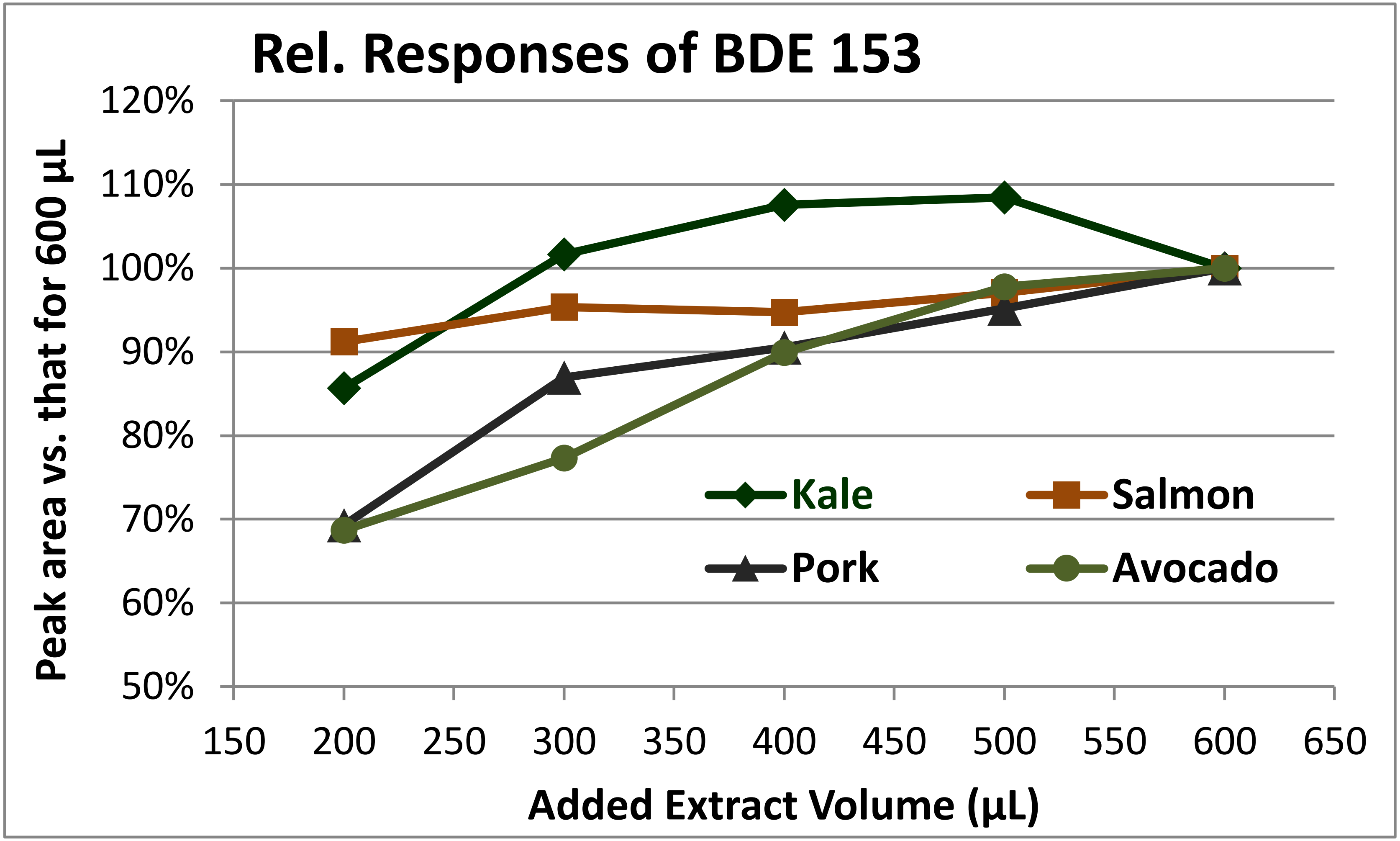

Conclusion: 300 µL extract needed for >70% elution of PBDEs

30 mg C_{18}+Z-Sep+CarbonX Mini-SPE of QuEChERS Extracts

Recovery vs. Extract Vol. Added

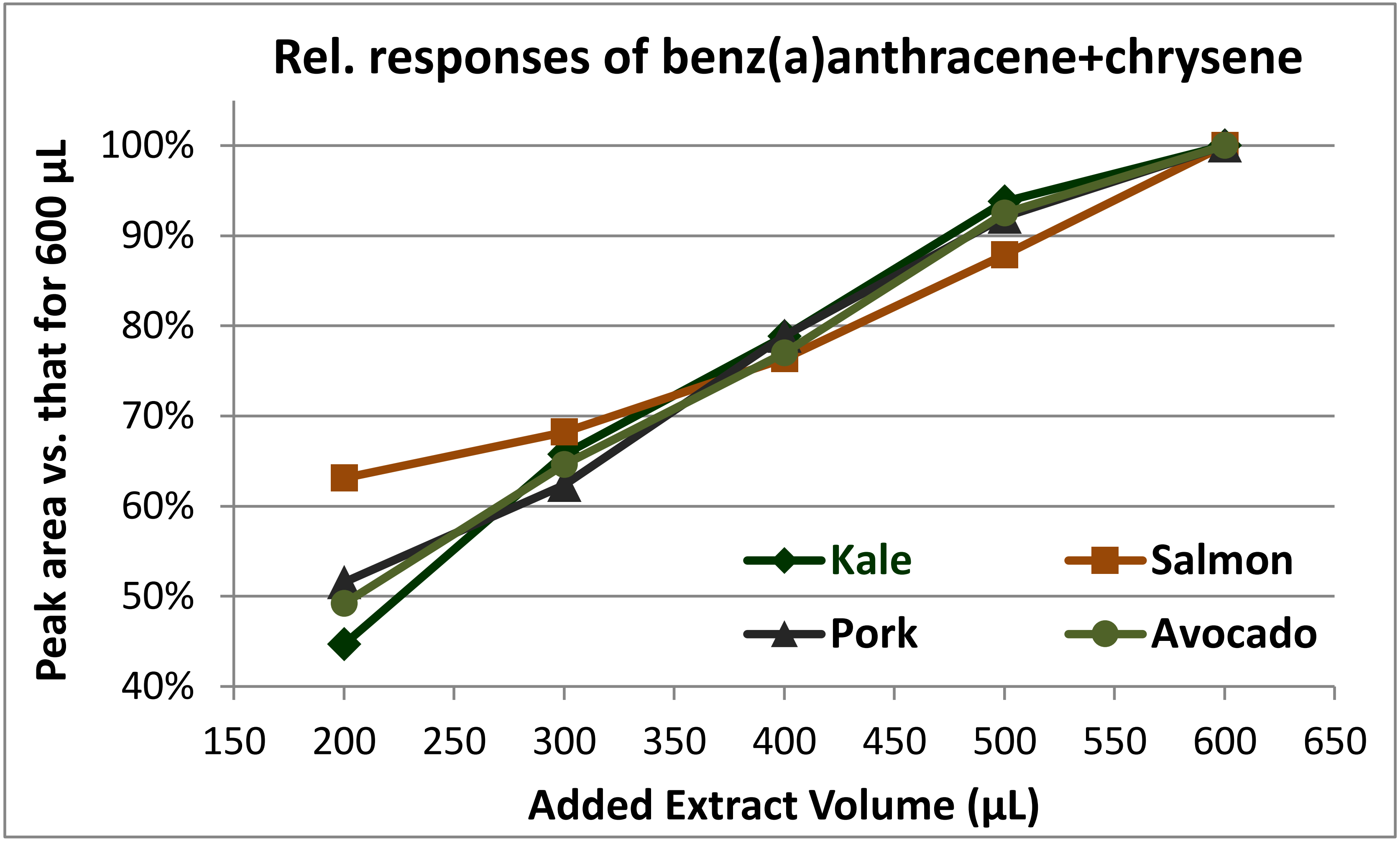

Conclusion: ≥600 µL extract needed for full elution of PAHs

45 mg $MgSO_4$+C_{18}+PSA+CarbonX Mini-SPE of QuEChERS Extracts

Recovery vs. Extract Vol. Added

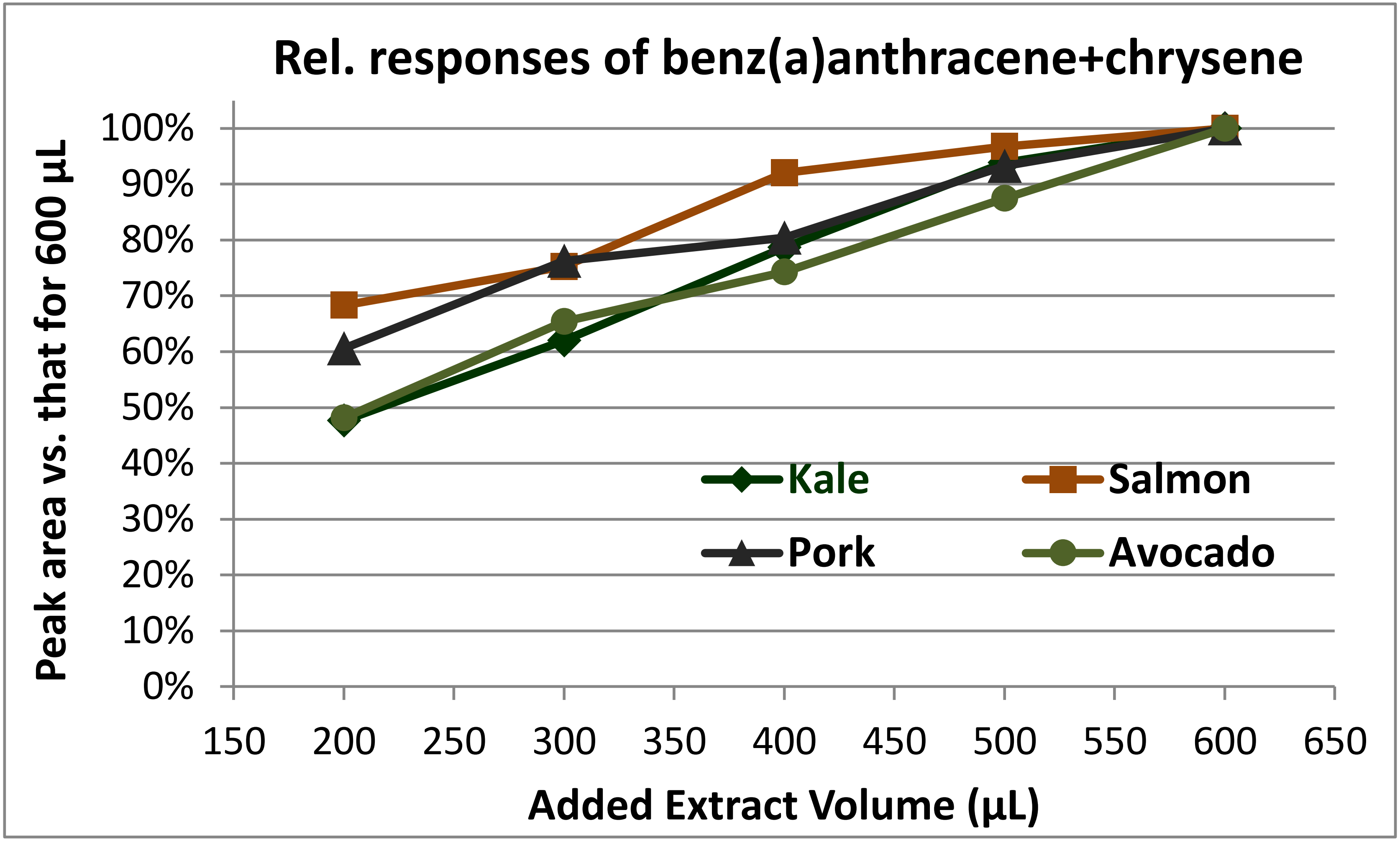

Conclusion: ≥600 µL extract needed for full elution of PAHs

Relative Recovery Differences for the Analytes in Mini-SPE for the 300 µL Extract Volumes vs. Avg. 600 µL Result with 45 mg mini-cartridge results – 30 mg mini-cartridge results (n = 12 for 4 matrices)

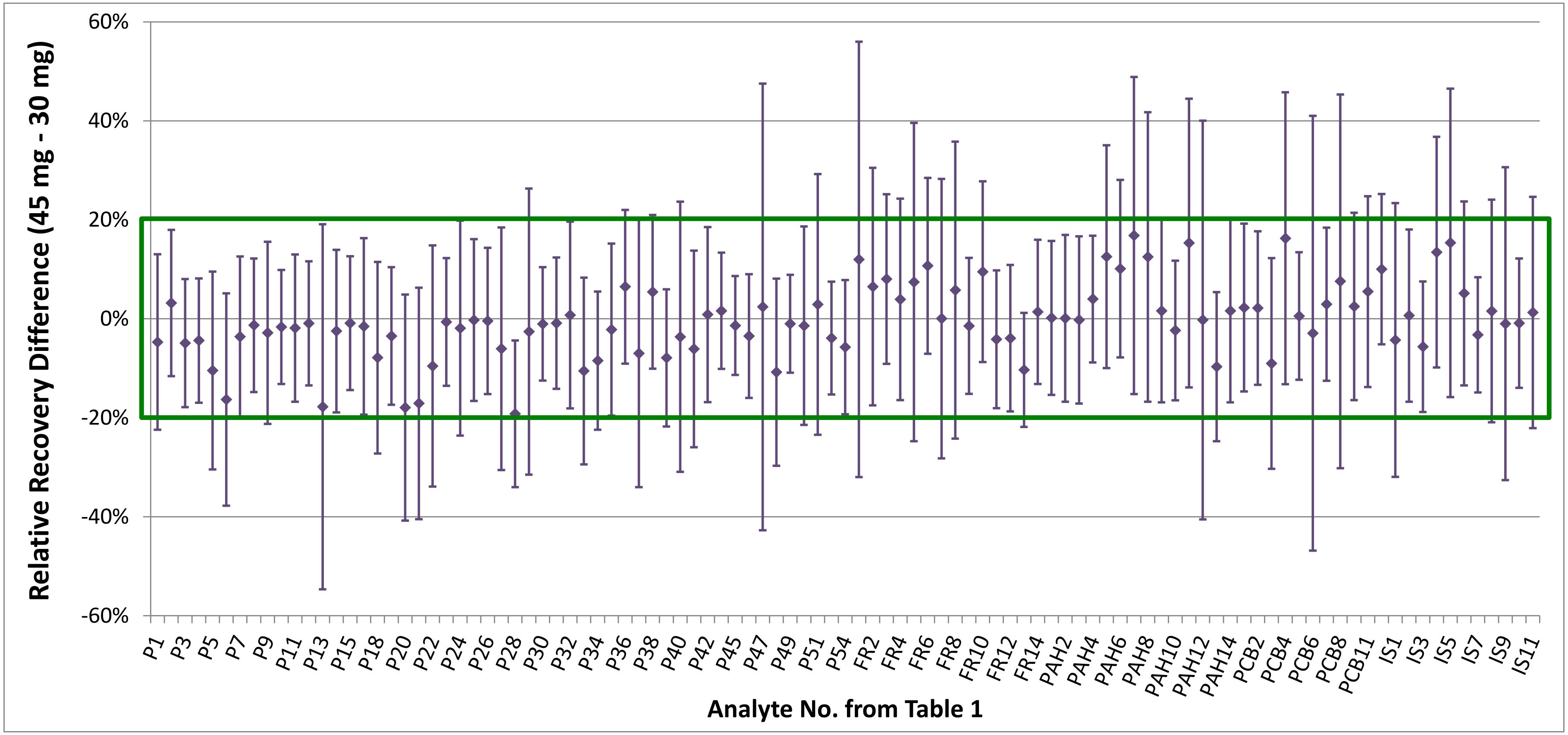

Conclusion: Similar results using either mini-cartridge

Validation Experiments

Determine performance results in the use of automated mini-SPE cleanup in the LPGC-MS/MS analysis of pesticides and other contaminants in QuEChERS extracts of 10 different matrices.

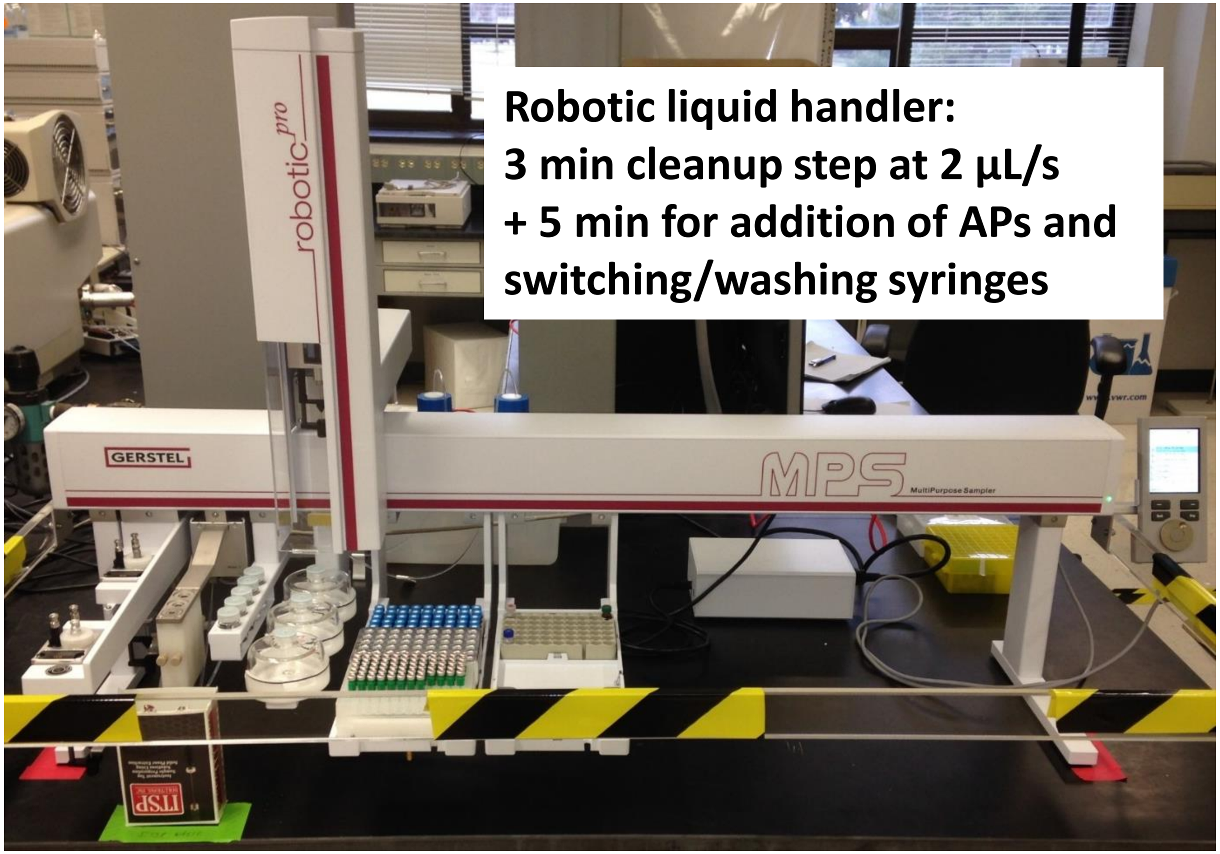

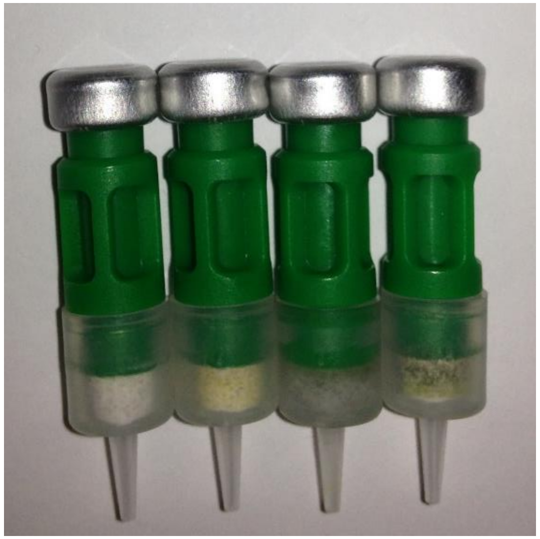

Used mini-cartridges showing removal of chlorophyll and other matrix components

Final extract volumes = 278 ± 5 µL (n = 255) after 50 µL addition of APs (and/MeCN) solution

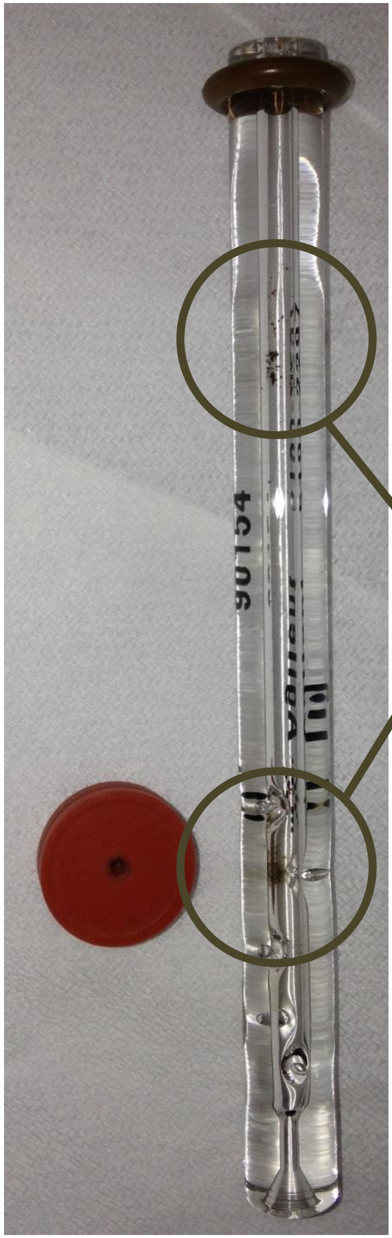

Injection liner and septa after the 5 day experiment including 230 matrix extracts of diverse commodities

A little “dirt” here and there, but the analyte protectants did their job and results still looked great from start to finish.

Coumaphos in salmon after 230 injections of QuEChERS matrix extracts using mini-SPE

No internal standard needed to still yield R^2 = 0.999 calibration curve

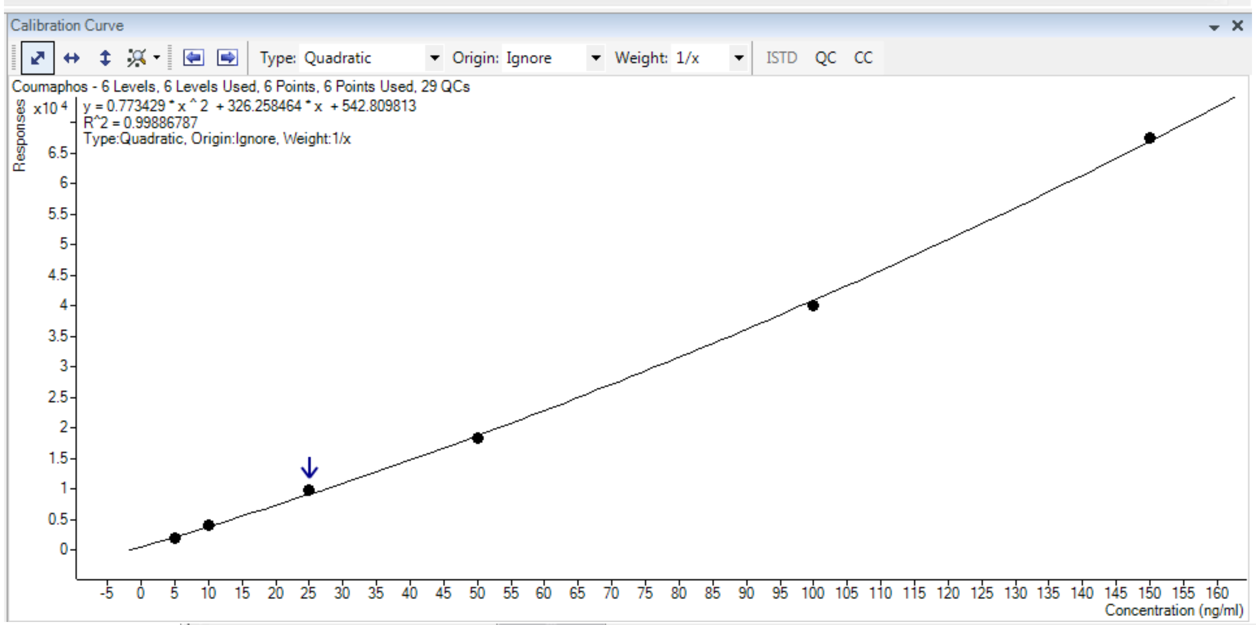

25 ng/mL std in salmon extract

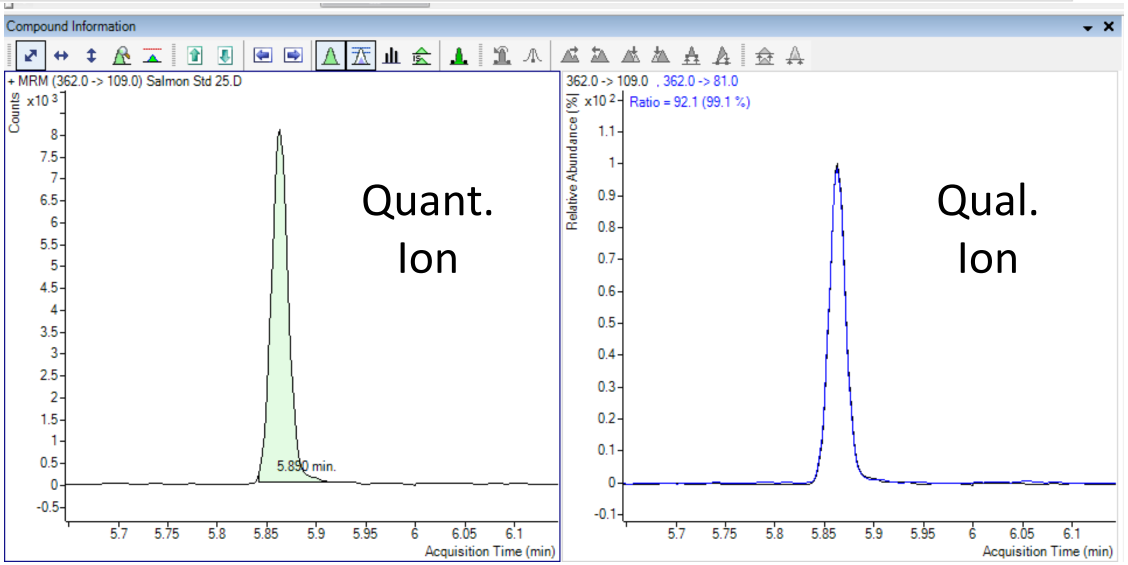

Methamidophos Results (vs. atrazine-d5 IS) – all 325 analyses combined!

Sample: Kale Std 10 | Sample Type: <All> | Compound: Methamidophos | ISTD: Atrazine-d5

Sample					Methami...	Methamidophos Results					Qualifier (95...		Atrazine-d5 (I...	
Name	Data File	Type	Level	Acq. Date-Time	Exp. Conc.	RT	Area	ISTD Resp. Ratio	Calc. Conc.	Accuracy	Area	Ratio	RT	Area
Carrot Std 50	Carrot Std 50.D	QC	5	2/23/2016 9:08 PM	50	2.855	23471	0.7438	44.9	90	20451	87	3.993	31555
Carrot Std 100	Carrot Std 100.D	QC	6	2/23/2016 9:22 PM	100	2.855	61531	1.9166	110.0	110	52894	86	3.993	32104
Carrot Std 150	Carrot Std 150.D	QC	7	2/23/2016 9:36 PM	150	2.855	54644	2.3325	131.6	88	47117	86	3.993	23427
Kale Spk 10a	Kale Spk 10a.D	MatrixSpike		2/24/2016 3:21 AM		2.855	3636	0.1258	7.5		2936	81	3.993	28906
Kale Spk 10b	Kale Spk 10b.D	MatrixSpike		2/24/2016 3:35 AM		2.855	6652	0.1506	9.1		5543	83	3.993	44173
Kale Spk 10c	Kale Spk 10c.D	MatrixSpike		2/24/2016 3:49 AM		2.855	3474	0.1278	7.6		2911	84	3.993	27197
Kale Spk 10d	Kale Spk 10d.D	MatrixSpike		2/24/2016 4:02 AM		2.855	15964	0.4024	24.6		13438	84	3.993	39676
Kale Spk 25a	Kale Spk 25a.D	MatrixSpike		2/24/2016 4:16 AM		2.855	7601	0.2889	17.6		6485	85	3.993	26314
Kale Spk 25b	Kale Spk 25b.D	MatrixSpike		2/24/2016 4:30 AM		2.855	4799	0.1404	8.4		3840	80	3.993	34180
Kale Spk 25c	Kale Spk 25c.D	MatrixSpike		2/24/2016 4:44 AM		2.855	23748	0.7074	42.8		19726	83	3.993	33570
Kale Spk 25d	Kale Spk 25d.D	MatrixSpike		2/24/2016 4:58 AM		2.855	13632	0.3564	21.8		11406	84	3.993	38251
Kale Spk 50a	Kale Spk 50a.D	MatrixSpike		2/24/2016 5:11 AM		2.855	12337	0.3277	20.0		10251	83	3.993	37648
Kale Spk 50b	Kale Spk 50b.D	MatrixSpike		2/24/2016 5:25 AM		2.855	19295	0.5220	31.8		16146	84	3.993	36961
Kale Spk 50c	Kale Spk 50c.D	MatrixSpike		2/24/2016 5:39 AM		2.855	14887	0.5485	33.4		12887	87	3.993	27140
Kale Spk 50d	Kale Spk 50d.D	MatrixSpike		2/24/2016 5:53 AM		2.855	23866	0.7328	44.3		20105	84	3.993	32569
Kale Spk 100a	Kale Spk 100a.D	MatrixSpike		2/24/2016 6:07 AM		2.855	32642	1.1923	70.6		27947	86	3.993	27378
Kale Spk 100b	Kale Spk 100b.D	MatrixSpike		2/24/2016 6:20 AM		2.855	29642	1.2824	75.7		24907	84	3.993	23115
Kale Spk 100c	Kale Spk 100c.D	MatrixSpike		2/24/2016 6:34 AM		2.855	33923	1.2526	74.0		28634	84	3.993	27082
Kale Spk 100d	Kale Spk 100d.D	MatrixSpike		2/24/2016 6:48 AM		2.855	53855	1.5988	93.0		45410	84	3.993	33686
Kale Std 0	Kale Std 0.D	QC	1	2/24/2016 1:44 AM		2.855	371	0.0153	0.5		442	119	3.993	24333
Kale Std 5	Kale Std 5.D	QC	2	2/24/2016 1:58 AM	5	2.855	2009	0.0751	4.3	86	1556	77	3.993	26751
Kale Std 10	Kale Std 10.D	QC	3	2/24/2016 2:12 AM	10	2.855	3912	0.1295	7.7	77	3244	83	3.993	30199
Kale Std 25	Kale Std 25.D	QC	4	2/24/2016 2:26 AM	25	2.855	27267	0.3868	23.6	94	23070	85	3.993	70490
Kale Std 50	Kale Std 50.D	QC	5	2/24/2016 2:40 AM	50	2.855	25164	0.6616	40.1	80	21428	85	3.993	38037

green boxes = atrazine-d5 (IS)

black circles = stds in solvent

blue triangles = matrix-matched stds

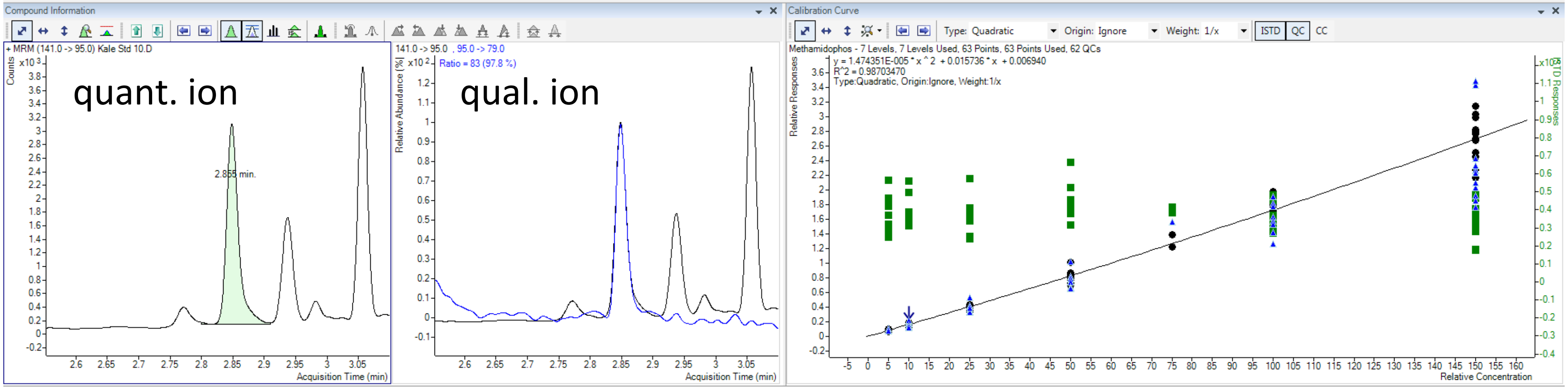

10 ng/mL std in kale

Acephate Results (vs. atrazine-d5 IS) – all 325 analyses combined!

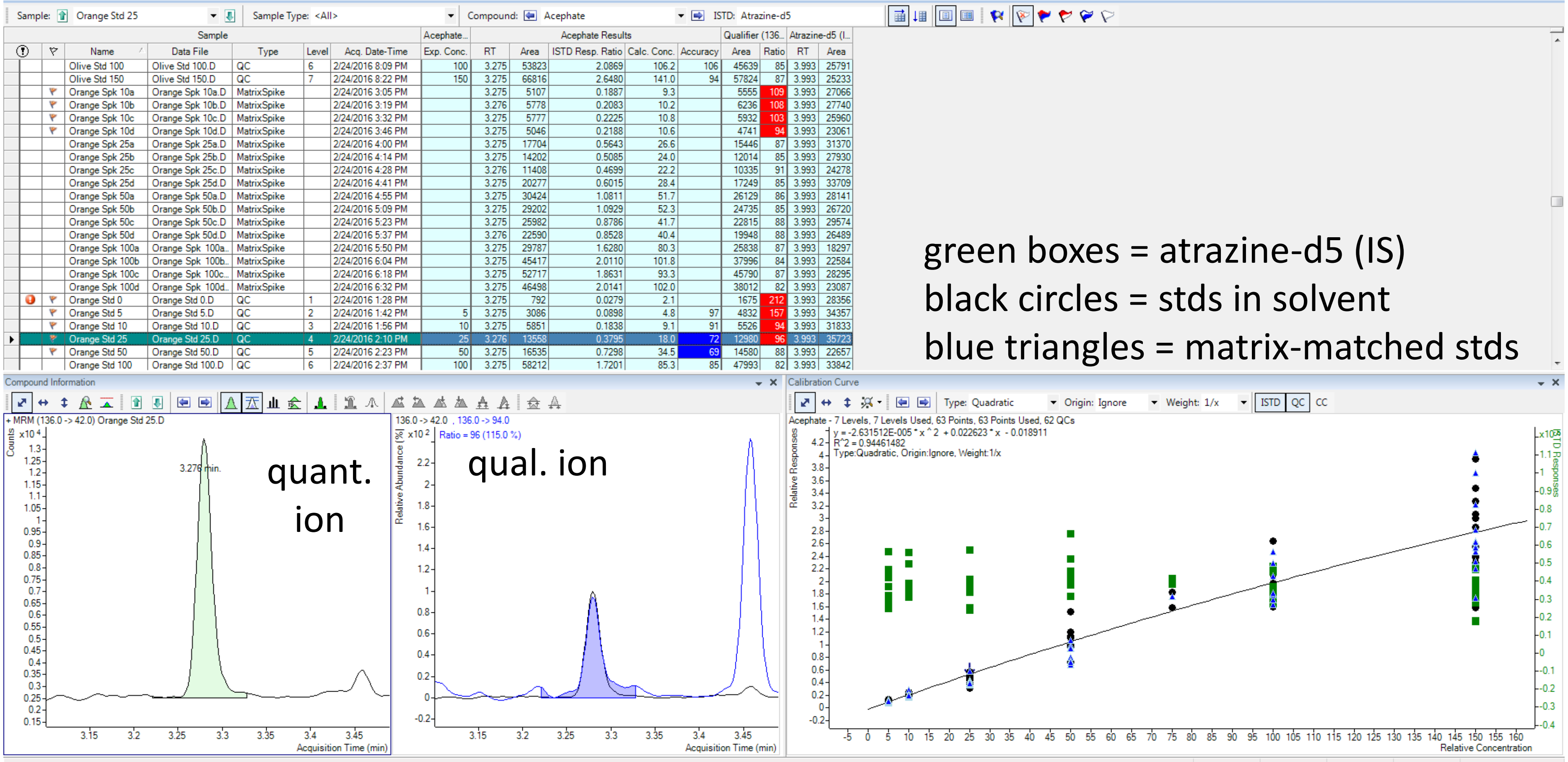

Sample: Orange Std 25 | Sample Type: <All> | Compound: Acephate | ISTD: Atrazine-d5

Sample							Acephate...	Acephate Results						Qualifier (136...		Atrazine-d5 (I...	
(!)	Flag	Name	Data File	Type	Level	Acq. Date-Time	Exp. Conc.	RT	Area	ISTD Resp. Ratio	Calc. Conc.	Accuracy	Area	Ratio	RT	Area
		Olive Std 100	Olive Std 100.D	QC	6	2/24/2016 8:09 PM	100	3.275	53823	2.0869	106.2	106	45639	85	3.993	25791
		Olive Std 150	Olive Std 150.D	QC	7	2/24/2016 8:22 PM	150	3.275	66816	2.6480	141.0	94	57824	87	3.993	25233
		Orange Spk 10a	Orange Spk 10a.D	MatrixSpike		2/24/2016 3:05 PM		3.275	5107	0.1887	9.3		5555	109	3.993	27066
		Orange Spk 10b	Orange Spk 10b.D	MatrixSpike		2/24/2016 3:19 PM		3.276	5778	0.2083	10.2		6236	108	3.993	27740
		Orange Spk 10c	Orange Spk 10c.D	MatrixSpike		2/24/2016 3:32 PM		3.275	5777	0.2225	10.8		5932	103	3.993	25960
		Orange Spk 10d	Orange Spk 10d.D	MatrixSpike		2/24/2016 3:46 PM		3.275	5046	0.2188	10.6		4741	94	3.993	23061
		Orange Spk 25a	Orange Spk 25a.D	MatrixSpike		2/24/2016 4:00 PM		3.275	17704	0.5643	26.6		15446	87	3.993	31370
		Orange Spk 25b	Orange Spk 25b.D	MatrixSpike		2/24/2016 4:14 PM		3.275	14202	0.5085	24.0		12014	85	3.993	27930
		Orange Spk 25c	Orange Spk 25c.D	MatrixSpike		2/24/2016 4:28 PM		3.276	11408	0.4699	22.2		10335	91	3.993	24278
		Orange Spk 25d	Orange Spk 25d.D	MatrixSpike		2/24/2016 4:41 PM		3.275	20277	0.6015	28.4		17249	85	3.993	33709
		Orange Spk 50a	Orange Spk 50a.D	MatrixSpike		2/24/2016 4:55 PM		3.275	30424	1.0811	51.7		26129	86	3.993	28141
		Orange Spk 50b	Orange Spk 50b.D	MatrixSpike		2/24/2016 5:09 PM		3.275	29202	1.0929	52.3		24735	85	3.993	26720
		Orange Spk 50c	Orange Spk 50c.D	MatrixSpike		2/24/2016 5:23 PM		3.275	25982	0.8786	41.7		22815	88	3.993	29574
		Orange Spk 50d	Orange Spk 50d.D	MatrixSpike		2/24/2016 5:37 PM		3.276	22590	0.8528	40.4		19948	88	3.993	26489
		Orange Spk 100a	Orange Spk 100a...	MatrixSpike		2/24/2016 5:50 PM		3.275	29787	1.6280	80.3		25838	87	3.993	18297
		Orange Spk 100b	Orange Spk 100b...	MatrixSpike		2/24/2016 6:04 PM		3.275	45417	2.0110	101.8		37996	84	3.993	22584
		Orange Spk 100c	Orange Spk 100c...	MatrixSpike		2/24/2016 6:18 PM		3.275	52717	1.8631	93.3		45790	87	3.993	28295
		Orange Spk 100d	Orange Spk 100d...	MatrixSpike		2/24/2016 6:32 PM		3.275	46498	2.0141	102.0		38012	82	3.993	23087
		Orange Std 0	Orange Std 0.D	QC	1	2/24/2016 1:28 PM		3.275	792	0.0279	2.1		1675	212	3.993	28356
		Orange Std 5	Orange Std 5.D	QC	2	2/24/2016 1:42 PM	5	3.275	3086	0.0898	4.8	97	4832	157	3.993	34357
		Orange Std 10	Orange Std 10.D	QC	3	2/24/2016 1:56 PM	10	3.275	5851	0.1838	9.1	91	5526	94	3.993	31833
		Orange Std 25	Orange Std 25.D	QC	4	2/24/2016 2:10 PM	25	3.276	13558	0.3795	18.0	72	12980	96	3.993	35723
		Orange Std 50	Orange Std 50.D	QC	5	2/24/2016 2:23 PM	50	3.275	16535	0.7298	34.5	69	14580	88	3.993	22657
		Orange Std 100	Orange Std 100.D	QC	6	2/24/2016 2:37 PM	100	3.275	58212	1.7201	85.3	85	47993	82	3.993	33842

25 ng/mL std in orange

Omethoate Results (vs. atrazine-d5 IS) – all 325 analyses combined!

Sample: Carrot Std 5 | Sample Type: <All> | Compound: Omethoate | ISTD: Atrazine-d5

Sample					Omethoa...	Omethoate Results					Qualifier (156...		Atrazine-d5 (I...	
Name	Data File	Type	Level	Acq. Date-Time	Exp. Conc.	RT	Area	ISTD Resp. Ratio	Calc. Conc.	Accuracy	Area	Ratio	RT	Area
Carrot Spk 10a	Carrot Spk 10a.D	MatrixSpike		2/23/2016 9:50 PM		3.666	23980	0.6894	8.1		18587	78	3.993	34782
Carrot Spk 10b	Carrot Spk 10b.D	MatrixSpike		2/23/2016 10:03 PM		3.666	31934	1.0520	11.8		26182	82	3.993	30356
Carrot Spk 10c	Carrot Spk 10c.D	MatrixSpike		2/23/2016 10:17 PM		3.666	26126	0.7517	8.7		21878	84	3.993	34754
Carrot Spk 10d	Carrot Spk 10d.D	MatrixSpike		2/23/2016 10:31 PM		3.666	21259	0.8172	9.4		16808	79	3.993	26014
Carrot Spk 25a	Carrot Spk 25a.D	MatrixSpike		2/23/2016 10:45 PM		3.666	41846	1.7612	19.2		35204	84	3.993	23760
Carrot Spk 25b	Carrot Spk 25b.D	MatrixSpike		2/23/2016 10:59 PM		3.666	46256	1.8344	19.9		38758	84	3.993	25216
Carrot Spk 25c	Carrot Spk 25c.D	MatrixSpike		2/23/2016 11:13 PM		3.666	30919	1.5299	16.8		25488	82	3.993	20210
Carrot Spk 25d	Carrot Spk 25d.D	MatrixSpike		2/23/2016 11:26 PM		3.666	39446	1.6743	18.3		33363	85	3.993	23560
Carrot Spk 50a	Carrot Spk 50a.D	MatrixSpike		2/23/2016 11:40 PM		3.666	95842	3.8386	41.4		82329	86	3.993	24968
Carrot Spk 50b	Carrot Spk 50b.D	MatrixSpike		2/23/2016 11:54 PM		3.666	71554	3.2315	34.8		60639	85	3.993	22142
Carrot Spk 50c	Carrot Spk 50c.D	MatrixSpike		2/24/2016 12:08 AM		3.666	253457	5.5575	60.7		215365	85	3.993	45606
Carrot Spk 50d	Carrot Spk 50d.D	MatrixSpike		2/24/2016 12:22 AM		3.666	97598	3.2626	35.2		82521	85	3.993	29914
Carrot Spk 100a	Carrot Spk 100a.D	MatrixSpike		2/24/2016 12:35 AM		3.666	184925	7.6107	85.0		162142	88	3.993	24298
Carrot Spk 100b	Carrot Spk 100b.D	MatrixSpike		2/24/2016 12:49 AM		3.666	541964	11.1126	130.1		470142	87	3.993	48770
Carrot Spk 100c	Carrot Spk 100c.D	MatrixSpike		2/24/2016 1:03 AM		3.666	203909	7.2572	80.7		177348	87	3.993	28097
Carrot Spk 100d	Carrot Spk 100d.D	MatrixSpike		2/24/2016 1:17 AM		3.666	236136	7.0397	78.1		203582	86	3.993	33543
Carrot Std 0	Carrot Std 0.D	QC	1	2/23/2016 8:13 PM		3.666	1302	0.0803	1.9		569	44	3.993	16229
Carrot Std 5	Carrot Std 5.D	QC	2	2/23/2016 8:27 PM	5	3.666	10683	0.4031	5.2	103	8444	79	3.993	26503
Carrot Std 10	Carrot Std 10.D	QC	3	2/23/2016 8:41 PM	10	3.666	17239	0.8211	9.4	94	13788	80	3.993	20996
Carrot Std 25	Carrot Std 25.D	QC	4	2/23/2016 8:54 PM	25	3.666	82564	1.8855	20.5	82	70702	86	3.993	43790
Carrot Std 50	Carrot Std 50.D	QC	5	2/23/2016 9:08 PM	50	3.666	103769	3.2885	35.4	71	88605	85	3.993	31555
Carrot Std 100	Carrot Std 100.D	QC	6	2/23/2016 9:22 PM	100	3.666	244262	7.6085	85.0	85	212035	87	3.993	32104
Carrot Std 150	Carrot Std 150.D	QC	7	2/23/2016 9:36 PM	150	3.666	300828	12.8411	154.6	103	263000	87	3.993	23427
Kale Spk 10a	Kale Spk 10a.D	MatrixSpike		2/24/2016 3:21 AM		3.666	19595	0.6779	8.0		17241	88	3.993	28906

black circles = stds in solvent

blue triangles = matrix-matched stds

Compound Information

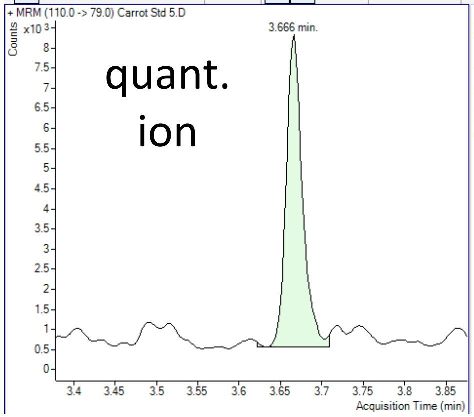

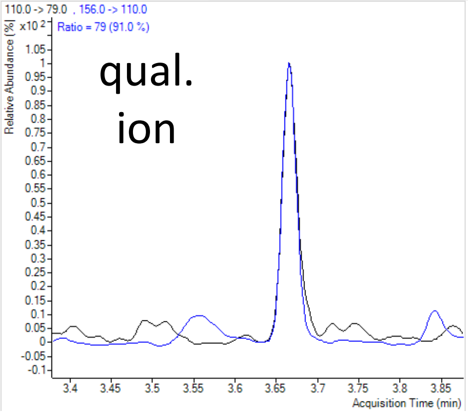

Calibration Curve

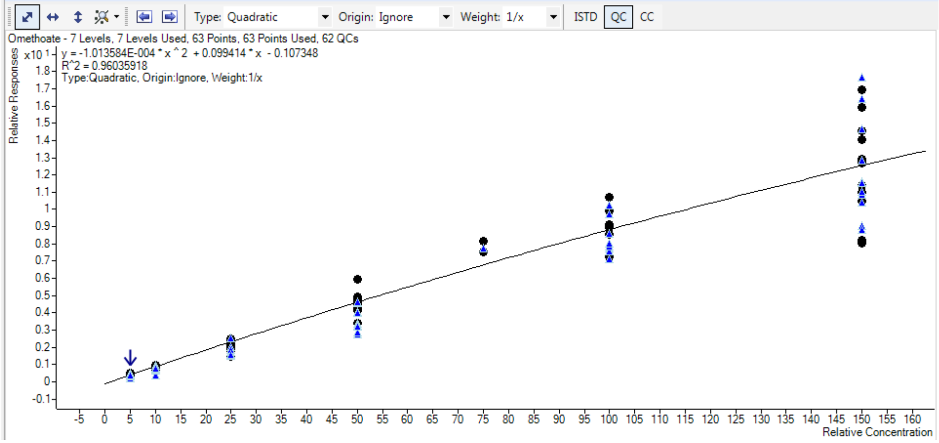

5 ng/mL std in carrot

Dicrotophos Results (vs. atrazine-d5 IS) – all 325 analyses combined!

Sample: Pork Std 5 | Sample Type: <All> | Compound: Dicrotophos | ISTD: Atrazine-d5

Sample					Dicrotoph...	Dicrotophos Results					Qualifier (127...		Atrazine-d5 (I...	
Name	Data File	Type	Level	Acq. Date-Time	Exp. Conc.	RT	Area	ISTD Resp. Ratio	Calc. Conc.	Accuracy	Area	Ratio	RT	Area
Orange Std 150	Orange Std 150.D	QC	7	2/24/2016 2:51 PM	150	3.828	584259	24.0214	149.2	99	260595	45	3.993	24322
Pork Spk 10a	Pork Spk 10a.D	MatrixSpike		2/26/2016 1:20 PM		3.828	36362	1.4710	9.0		15407	42	3.993	24718
Pork Spk 10b	Pork Spk 10b.D	MatrixSpike		2/26/2016 1:34 PM		3.828	43939	1.3847	8.5		19487	44	3.993	31732
Pork Spk 10c	Pork Spk 10c.D	MatrixSpike		2/26/2016 1:48 PM		3.828	35519	1.3298	8.1		15350	43	3.993	26710
Pork Spk 10d	Pork Spk 10d.D	MatrixSpike		2/26/2016 2:02 PM		3.828	59314	1.5902	9.8		25714	43	3.993	37301
Pork Spk 25a	Pork Spk 25a.D	MatrixSpike		2/26/2016 2:16 PM		3.828	80625	3.2677	20.3		35735	44	3.993	24673
Pork Spk 25b	Pork Spk 25b.D	MatrixSpike		2/26/2016 2:29 PM		3.828	78979	3.3353	20.7		34686	44	3.993	23680
Pork Spk 25c	Pork Spk 25c.D	MatrixSpike		2/26/2016 2:43 PM		3.828	96000	3.4685	21.6		42076	44	3.993	27678
Pork Spk 25d	Pork Spk 25d.D	MatrixSpike		2/26/2016 2:57 PM		3.828	108872	3.5257	21.9		46206	42	3.993	30880
Pork Spk 50a	Pork Spk 50a.D	MatrixSpike		2/26/2016 3:11 PM		3.828	208487	6.9528	43.4		90742	44	3.993	29986
Pork Spk 50b	Pork Spk 50b.D	MatrixSpike		2/26/2016 3:25 PM		3.828	233484	7.3242	45.7		101498	43	3.993	31878
Pork Spk 50c	Pork Spk 50c.D	MatrixSpike		2/26/2016 3:38 PM		3.828	367678	8.1614	51.0		161669	44	3.993	45051
Pork Spk 50d	Pork Spk 50d.D	MatrixSpike		2/26/2016 3:52 PM		3.828	198169	6.8791	43.0		87650	44	3.993	28808
Pork Spk 100a	Pork Spk 100a.D	MatrixSpike		2/26/2016 4:06 PM		3.828	640092	16.0735	100.2		281914	44	3.993	39823
Pork Spk 100b	Pork Spk 100b.D	MatrixSpike		2/26/2016 4:20 PM		3.828	555336	15.2830	95.3		242533	44	3.993	36337
Pork Spk 100c	Pork Spk 100c.D	MatrixSpike		2/26/2016 4:34 PM		3.828	334976	14.8246	92.4		146863	44	3.993	22596
Pork Spk 100d	Pork Spk 100d.D	MatrixSpike		2/26/2016 4:48 PM		3.828	533627	14.8127	92.4		235560	44	3.993	36025
Pork Std 0	Pork Std 0.D	QC	1	2/26/2016 11:43 AM		3.828	1998	0.0716	0.2		612	31	3.993	27916
Pork Std 5	Pork Std 5.D	QC	2	2/26/2016 11:57 AM	5	3.828	16554	0.7633	4.6	91	6890	42	3.993	21688
Pork Std 10	Pork Std 10.D	QC	3	2/26/2016 12:11 PM	10	3.828	32739	1.3862	8.5	85	14164	43	3.993	23617
Pork Std 25	Pork Std 25.D	QC	4	2/26/2016 12:25 PM	25	3.828	86211	3.4449	21.4	86	37836	44	3.993	25026
Pork Std 50	Pork Std 50.D	QC	5	2/26/2016 12:39 PM	50	3.828	215532	6.7441	42.1	84	92205	43	3.993	31959
Pork Std 100	Pork Std 100.D	QC	6	2/26/2016 12:53 PM	100	3.828	684614	17.9721	111.9	112	301446	44	3.993	38093
Pork Std 150	Pork Std 150.D	QC	7	2/26/2016 1:06 PM	150	3.828	619199	22.6815	140.9	94	273726	44	3.993	27300

black circles = stds in solvent

blue triangles = matrix-matched stds

Compound Information

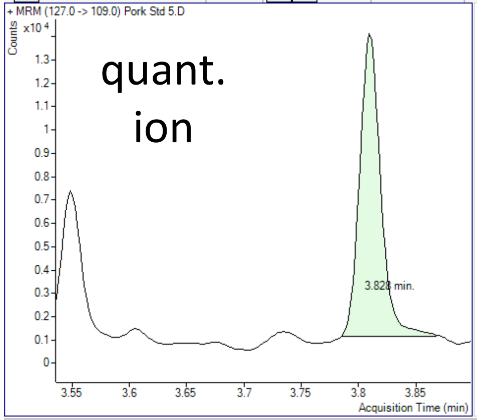

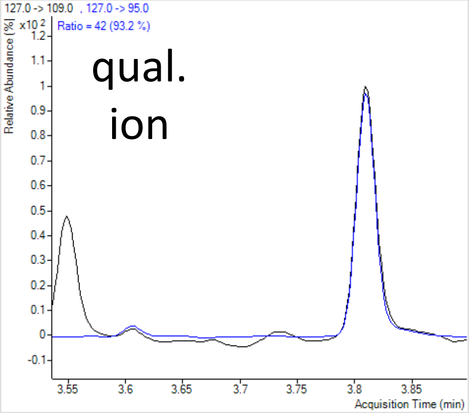

Calibration Curve

Type: Quadratic Origin: Ignore Weight: 1/x

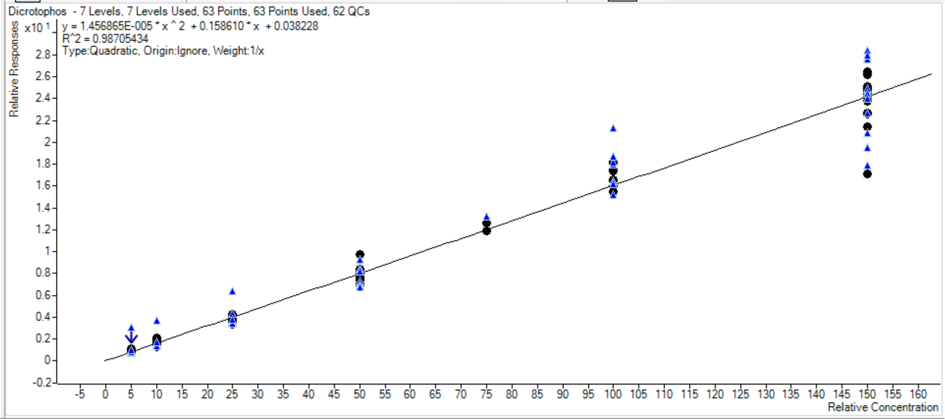

5 ng/mL std in pork

Dimethoate Results (vs. atrazine-d5 IS) – all 325 analyses combined!

Sample: Kiwi Std 5 | Sample Type: <All> | Compound: Dimethoate | ISTD: Atrazine-d5

Sample							Dimethoa...	Dimethoate Results					Qualifier (125...		Atrazine-d5 (I...	
(!)	Flag	Name	Data File	Type	Level	Acq. Date-Time	Exp. Conc.	RT	Area	ISTD Resp. Ratio	Calc. Conc.	Accuracy	Area	Ratio	RT	Area
		Kale Std 150	Kale Std 150.D	QC	7	2/24/2016 3:07 AM	150	3.978	143373	5.6506	146.9	98	5537	4	3.993	25373
		Kiwi Spk 10a	Kiwi Spk 10a.D	MatrixSpike		2/23/2016 4:43 AM		3.979	13221	0.3427	8.4		621	5	3.993	38579
		Kiwi Spk 10b	Kiwi Spk 10b.D	MatrixSpike		2/23/2016 4:58 AM		3.978	12833	0.3525	8.7		565	4	3.993	36410
		Kiwi Spk 10c	Kiwi Spk 10c.D	MatrixSpike		2/23/2016 5:13 AM		3.979	10379	0.3418	8.4		493	5	3.993	30368
		Kiwi Spk 10d	Kiwi Spk 10d.D	MatrixSpike		2/23/2016 5:29 AM		3.978	14012	0.3721	9.2		565	4	3.993	37655
		Kiwi Spk 25a	Kiwi Spk 25a.D	MatrixSpike		2/23/2016 5:44 AM		3.979	32845	0.9202	23.5		1082	3	3.993	35693
		Kiwi Spk 25b	Kiwi Spk 25b.D	MatrixSpike		2/23/2016 5:59 AM		3.978	34693	0.9105	23.2		1327	4	3.993	38103
		Kiwi Spk 25c	Kiwi Spk 25c.D	MatrixSpike		2/23/2016 6:14 AM		3.979	42009	0.9174	23.4		1697	4	3.993	45790
		Kiwi Spk 25d	Kiwi Spk 25d.D	MatrixSpike		2/23/2016 6:29 AM		3.978	36377	0.9025	23.0		1284	4	3.993	40307
		Kiwi Spk 50a	Kiwi Spk 50a.D	MatrixSpike		2/23/2016 6:44 AM		3.979	78970	1.8517	47.7		2926	4	3.993	42647
		Kiwi Spk 50b	Kiwi Spk 50b.D	MatrixSpike		2/23/2016 6:59 AM		3.978	68028	1.8057	46.5		2484	4	3.993	37674
		Kiwi Spk 50c	Kiwi Spk 50c.D	MatrixSpike		2/23/2016 7:14 AM		3.979	70478	1.7888	46.1		2398	3	3.993	39399
		Kiwi Spk 50d	Kiwi Spk 50d.D	MatrixSpike		2/23/2016 7:29 AM		3.978	73314	1.8142	46.7		2704	4	3.993	40410
		Kiwi Spk 100a	Kiwi Spk 100a.D	MatrixSpike		2/23/2016 7:44 AM		3.979	144412	4.2370	110.0		5310	4	3.993	34084
		Kiwi Spk 100b	Kiwi Spk 100b.D	MatrixSpike		2/23/2016 7:59 AM		3.978	339807	4.3677	113.4		12390	4	3.993	77800
		Kiwi Spk 100c	Kiwi Spk 100c.D	MatrixSpike		2/23/2016 8:14 AM		3.979	156411	4.2109	109.3		5925	4	3.993	37144
		Kiwi Spk 100d	Kiwi Spk 100d.D	MatrixSpike		2/23/2016 8:29 AM		3.978	160110	4.1927	108.8		5693	4	3.993	38188
!	Flag	Kiwi Std 0	Kiwi Std 0.D	QC	1	2/23/2016 2:58 AM		3.978	919	0.0210	0.1		131	14	3.993	43731
!		Kiwi Std 5	Kiwi Std 5.D	QC	2	2/23/2016 3:13 AM	5	3.979	8735	0.2170	5.2	103	395	5	3.993	40248
		Kiwi Std 10	Kiwi Std 10.D	QC	3	2/23/2016 3:28 AM	10	3.978	15736	0.3885	9.6	96	698	4	3.993	40501
		Kiwi Std 25	Kiwi Std 25.D	QC	4	2/23/2016 3:43 AM	25	3.979	59080	1.0207	26.1	104	1969	3	3.993	57880
		Kiwi Std 50	Kiwi Std 50.D	QC	5	2/23/2016 3:58 AM	50	3.978	86186	1.9695	50.8	102	3286	4	3.993	43760
		Kiwi Std 100	Kiwi Std 100.D	QC	8	2/23/2016 4:13 AM	75	3.979	143809	3.1749	82.2	110	5426	4	3.993	45295
		Kiwi Std 150	Kiwi Std 150.D	QC	7	2/23/2016 4:28 AM	150	3.978	253167	6.3801	166.0	111	9449	4	3.993	39681

black circles = stds in solvent

blue triangles = matrix-matched stds

Compound Information

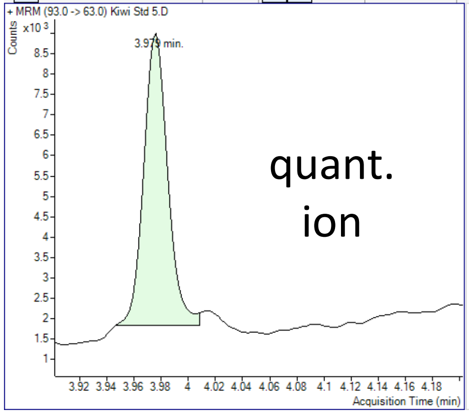

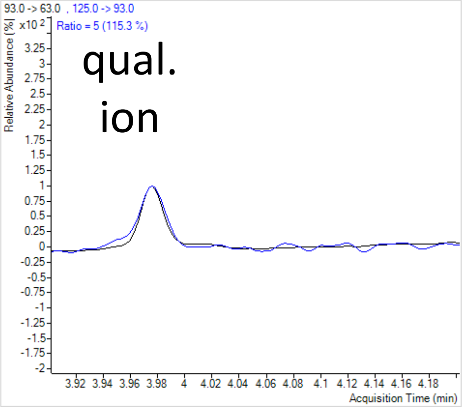

Calibration Curve

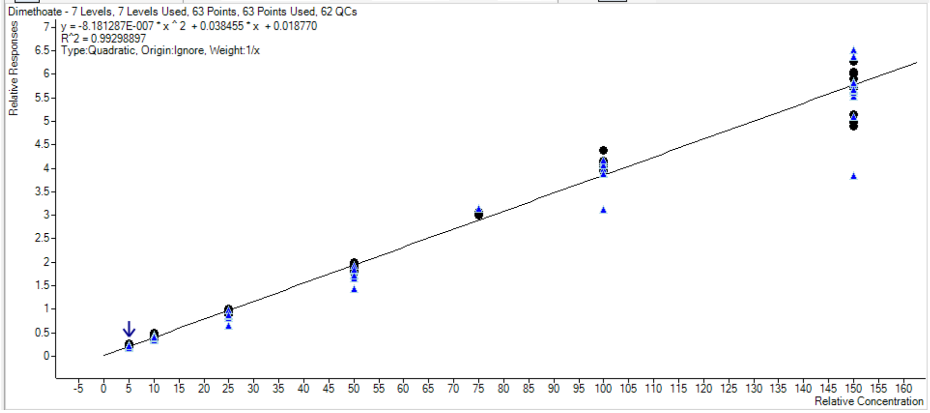

5 ng/mL std in kiwi

Carbofuran Results (vs. atrazine-d5 IS) – all 325 analyses combined!

Sample: Orange Std 10 | Sample Type: <All> | Compound: Carbofuran | ISTD: Atrazine-d5

Sample					Carbofur...	Carbofuran Results					Qualifier (164...		Atrazine-d5 (I...	
Name	Data File	Type	Level	Acq. Date-Time	Exp. Conc.	RT	Area	ISTD Resp. Ratio	Calc. Conc.	Accuracy	Area	Ratio	RT	Area
Olive Std 50	Olive Std 50.D	QC	5	2/24/2016 7:55 PM	50	3.985	60476	1.4855	43.2	86	55395	92	3.993	40710
Olive Std 100	Olive Std 100.D	QC	6	2/24/2016 8:09 PM	100	3.985	74133	2.8744	84.7	85	68002	92	3.993	25791
Olive Std 150	Olive Std 150.D	QC	7	2/24/2016 8:22 PM	150	3.985	97288	3.8556	114.7	76	88300	91	3.993	25233
Orange Spk 10a	Orange Spk 10a.D	MatrixSpike		2/24/2016 3:05 PM		3.985	12211	0.4512	13.0		11103	91	3.993	27066
Orange Spk 10b	Orange Spk 10b.D	MatrixSpike		2/24/2016 3:19 PM		3.985	12152	0.4381	12.6		11288	93	3.993	27740
Orange Spk 10c	Orange Spk 10c.D	MatrixSpike		2/24/2016 3:32 PM		3.985	11982	0.4616	13.3		11524	96	3.993	25960
Orange Spk 10d	Orange Spk 10d.D	MatrixSpike		2/24/2016 3:46 PM		3.985	10678	0.4630	13.3		9548	89	3.993	23061
Orange Spk 25a	Orange Spk 25a.D	MatrixSpike		2/24/2016 4:00 PM		3.985	32578	1.0385	30.1		29264	90	3.993	31370
Orange Spk 25b	Orange Spk 25b.D	MatrixSpike		2/24/2016 4:14 PM		3.985	27425	0.9819	28.4		24847	91	3.993	27930
Orange Spk 25c	Orange Spk 25c.D	MatrixSpike		2/24/2016 4:28 PM		3.985	22194	0.9142	26.5		20186	91	3.993	24278
Orange Spk 25d	Orange Spk 25d.D	MatrixSpike		2/24/2016 4:41 PM		3.985	35002	1.0384	30.1		31842	91	3.993	33709
Orange Spk 50a	Orange Spk 50a.D	MatrixSpike		2/24/2016 4:55 PM		3.985	57448	2.0415	59.7		50298	88	3.993	28141
Orange Spk 50b	Orange Spk 50b.D	MatrixSpike		2/24/2016 5:09 PM		3.985	53065	1.9860	58.1		47752	90	3.993	26720
Orange Spk 50c	Orange Spk 50c.D	MatrixSpike		2/24/2016 5:23 PM		3.985	52767	1.7843	52.1		47835	91	3.993	29574
Orange Spk 50d	Orange Spk 50d.D	MatrixSpike		2/24/2016 5:37 PM		3.985	47564	1.7956	52.4		41535	87	3.993	26489
Orange Spk 100a	Orange Spk 100a...	MatrixSpike		2/24/2016 5:50 PM		3.985	62479	3.4146	101.2		55435	89	3.993	18297
Orange Spk 100b	Orange Spk 100b...	MatrixSpike		2/24/2016 6:04 PM		3.985	88336	3.9115	116.4		79187	90	3.993	22584
Orange Spk 100c	Orange Spk 100c...	MatrixSpike		2/24/2016 6:18 PM		3.985	109143	3.8573	114.8		98099	90	3.993	28295
Orange Spk 100d	Orange Spk 100d...	MatrixSpike		2/24/2016 6:32 PM		3.985	88627	3.8388	114.2		79675	90	3.993	23087
Orange Std 0	Orange Std 0.D	QC	1	2/24/2016 1:28 PM		3.985	1429	0.0504	1.4		2318	162	3.993	28356
Orange Std 5	Orange Std 5.D	QC	2	2/24/2016 1:42 PM	5	3.985	6828	0.1987	5.7	114	6351	93	3.993	34357
Orange Std 10	Orange Std 10.D	QC	3	2/24/2016 1:56 PM	10	3.985	10554	0.3315	9.5	95	9623	91	3.993	31833
Orange Std 25	Orange Std 25.D	QC	4	2/24/2016 2:10 PM	25	3.985	26959	0.7547	21.8	87	24744	92	3.993	35723
Orange Std 50	Orange Std 50.D	QC	5	2/24/2016 2:23 PM	50	3.985	32429	1.4313	41.6	83	28557	88	3.993	22657

black circles = stds in solvent

blue triangles = matrix-matched stds

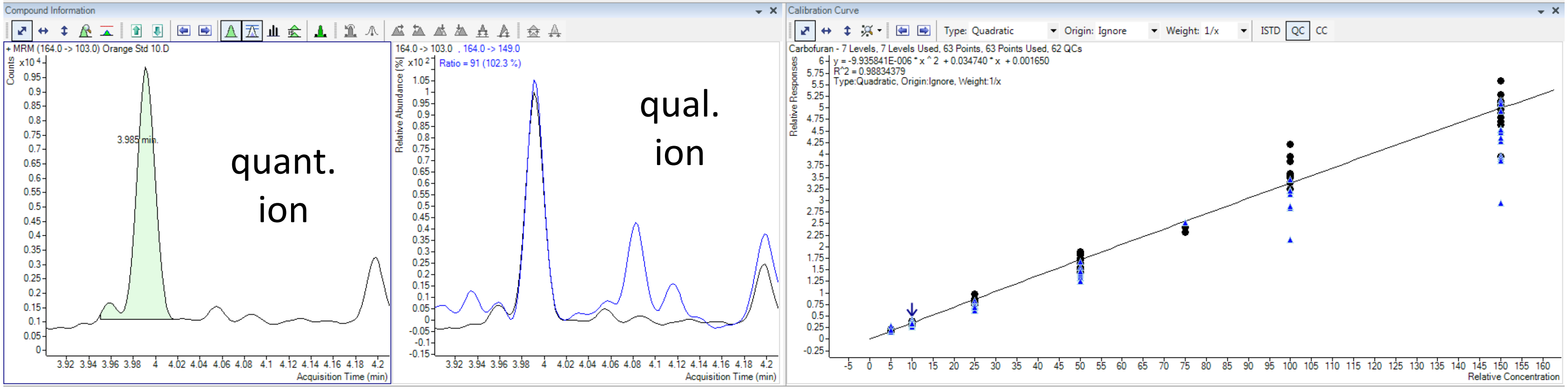

10 ng/mL std in orange

Diazinon Results (vs. atrazine-d5 IS) – all 325 analyses combined!

Sample: Wheat Std 10 | Sample Type: <All> | Compound: Diazinon | ISTD: Atrazine-d5

Sample						Diazinon...	Diazinon Results						Qualifier (304...		Atrazine-d5 (I...	
Name	Data File	Type	Level	Acq. Date-Time		Exp. Conc.	RT	Area	ISTD Resp. Ratio	Calc. Conc.	Accuracy		Area	Ratio	RT	Area
SO Std 150b WB	SO Std 150b WB....	Cal	7	2/26/2016 4:11 AM		150	4.111	65894	1.7462	145.6	97		63601	97	3.993	37736
Wheat Spk 10a	Wheat Spk 10a.D	MatrixSpike		2/25/2016 5:36 PM			4.111	3187	0.1268	10.9			2777	87	3.993	25122
Wheat Spk 10b	Wheat Spk 10b.D	MatrixSpike		2/25/2016 5:50 PM			4.111	4299	0.1293	11.1			3697	86	3.993	33246
Wheat Spk 10c	Wheat Spk 10c.D	MatrixSpike		2/25/2016 6:03 PM			4.111	4961	0.1265	10.9			4281	86	3.993	39230
Wheat Spk 10d	Wheat Spk 10d.D	MatrixSpike		2/25/2016 6:17 PM			4.111	3717	0.1228	10.5			3470	93	3.993	30280
Wheat Spk 25a	Wheat Spk 25a.D	MatrixSpike		2/25/2016 6:31 PM			4.111	9873	0.2921	25.7			9431	96	3.993	33795
Wheat Spk 25b	Wheat Spk 25b.D	MatrixSpike		2/25/2016 6:45 PM			4.111	11616	0.2834	25.0			11002	95	3.993	40991
Wheat Spk 25c	Wheat Spk 25c.D	MatrixSpike		2/25/2016 6:59 PM			4.111	7305	0.3064	27.0			6423	88	3.993	23843
Wheat Spk 25d	Wheat Spk 25d.D	MatrixSpike		2/25/2016 7:12 PM			4.111	7257	0.2919	25.7			6508	90	3.993	24861
Wheat Spk 50a	Wheat Spk 50a.D	MatrixSpike		2/25/2016 7:26 PM			4.111	23376	0.5713	50.2			21888	94	3.993	40921
Wheat Spk 50b	Wheat Spk 50b.D	MatrixSpike		2/25/2016 7:40 PM			4.111	12566	0.5706	50.1			12165	97	3.993	22021
Wheat Spk 50c	Wheat Spk 50c.D	MatrixSpike		2/25/2016 7:54 PM			4.111	17877	0.6096	53.5			16376	92	3.993	29327
Wheat Spk 50d	Wheat Spk 50d.D	MatrixSpike		2/25/2016 8:08 PM			4.111	18152	0.5805	51.0			17438	96	3.993	31270
Wheat Spk 100a	Wheat Spk 100a.D	MatrixSpike		2/25/2016 8:22 PM			4.111	52548	1.2273	104.8			49375	94	3.993	42817
Wheat Spk 100b	Wheat Spk 100b.D	MatrixSpike		2/25/2016 8:35 PM			4.111	34827	1.2103	103.4			32833	94	3.993	28776
Wheat Spk 100c	Wheat Spk 100c.D	MatrixSpike		2/25/2016 8:49 PM			4.111	44267	1.2230	104.4			41085	93	3.993	36194
Wheat Spk 100d	Wheat Spk 100d.D	MatrixSpike		2/25/2016 9:03 PM			4.111	32700	1.2480	106.4			30929	95	3.993	26201
Wheat Std 0	Wheat Std 0.D	QC	1	2/25/2016 3:59 PM			4.111	501	0.0204	1.2			239	48	3.993	24540
Wheat Std 5	Wheat Std 5.D	QC	2	2/25/2016 4:13 PM		5	4.111	1820	0.0763	6.3	126		1387	76	3.993	23867
Wheat Std 10	Wheat Std 10.D	QC	3	2/25/2016 4:27 PM		10	4.111	5221	0.1273	10.9	109		4472	86	3.993	41019
Wheat Std 25	Wheat Std 25.D	QC	4	2/25/2016 4:40 PM		25	4.111	7410	0.3007	26.5	106		6751	91	3.993	24647
Wheat Std 50	Wheat Std 50.D	QC	5	2/25/2016 4:54 PM		50	4.111	25091	0.5728	50.3	101		23324	93	3.993	43802
Wheat Std 100	Wheat Std 100.D	QC	6	2/25/2016 5:08 PM		100	4.111	46591	1.2232	104.4	104		44131	95	3.993	38089
Wheat Std 150	Wheat Std 150.D	QC	7	2/25/2016 5:22 PM		150	4.111	70329	1.6934	141.5	94		66223	94	3.993	41532

black circles = stds in solvent

blue triangles = matrix-matched stds

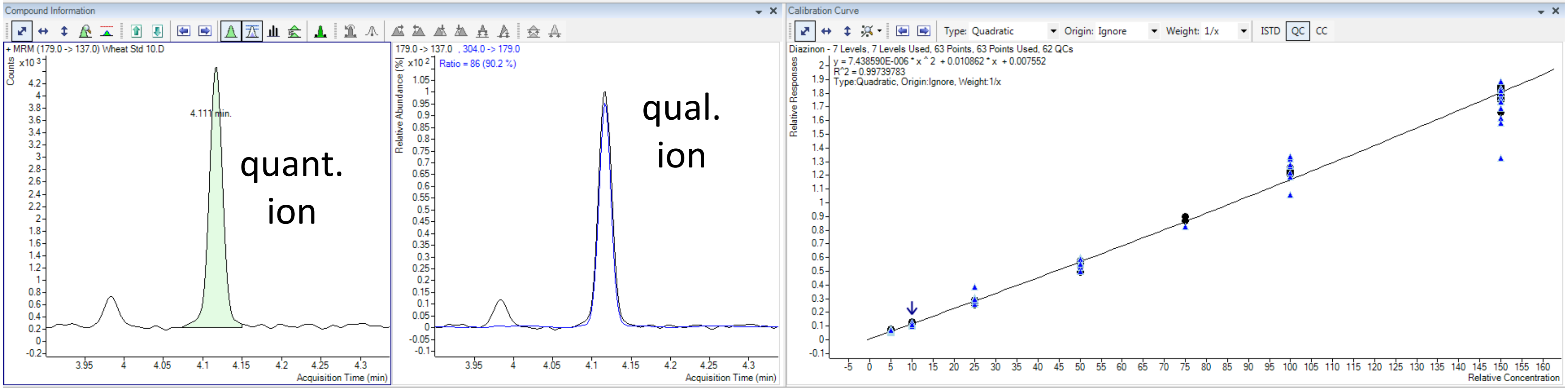

10 ng/mL std in wheat

Chlorothalonil Results (vs. atrazine-d5 IS) – all 325 analyses combined!

Sample: Basil Std 5 | Sample Type: <All> | Compound: Chlorothalonil | ISTD: Atrazine-d5

Sample					Chloroth...	Chlorothalonil Results					Qualifier (266...		Atrazine-d5 (I...	
Name	Data File	Type	Level	Acq. Date-Time	Exp. Conc.	RT	Area	ISTD Resp. Ratio	Calc. Conc.	Accuracy	Area	Ratio	RT	Area
Basil Spk 10d	Basil Spk 10d.D	MatrixSpike		2/25/2016 11:49 PM		4.220	7133	0.1611	5.7		4255	60	3.993	44282
Basil Spk 25a	Basil Spk 25a.D	MatrixSpike		2/26/2016 12:03 AM		4.220	11710	0.4015	14.5		6285	54	3.993	29164
Basil Spk 25b	Basil Spk 25b.D	MatrixSpike		2/26/2016 12:16 AM		4.220	14294	0.4054	14.6		7706	54	3.993	35256
Basil Spk 25c	Basil Spk 25c.D	MatrixSpike		2/26/2016 12:30 AM		4.220	9879	0.3856	13.9		5407	55	3.993	25618
Basil Spk 25d	Basil Spk 25d.D	MatrixSpike		2/26/2016 12:44 AM		4.220	11194	0.4263	15.4		5798	52	3.993	26260
Basil Spk 50a	Basil Spk 50a.D	MatrixSpike		2/26/2016 12:58 AM		4.220	35880	0.7342	26.7		19522	54	3.993	48873
Basil Spk 50b	Basil Spk 50b.D	MatrixSpike		2/26/2016 1:12 AM		4.220	21501	0.8549	31.2		11718	54	3.993	25150
Basil Spk 50c	Basil Spk 50c.D	MatrixSpike		2/26/2016 1:26 AM		4.220	23556	0.8241	30.0		12439	53	3.993	28583
Basil Spk 50d	Basil Spk 50d.D	MatrixSpike		2/26/2016 1:39 AM		4.220	25332	0.8519	31.1		13818	55	3.993	29736
Basil Spk 100a	Basil Spk 100a.D	MatrixSpike		2/26/2016 1:53 AM		4.220	62345	1.8277	68.0		34521	55	3.993	34111
Basil Spk 100b	Basil Spk 100b.D	MatrixSpike		2/26/2016 2:07 AM		4.220	60651	1.8963	70.6		32719	54	3.993	31984
Basil Spk 100c	Basil Spk 100c.D	MatrixSpike		2/26/2016 2:21 AM		4.220	109616	1.9473	72.6		59734	54	3.993	56290
Basil Spk 100d	Basil Spk 100d.D	MatrixSpike		2/26/2016 2:35 AM		4.220	61504	1.8889	70.3		33309	54	3.993	32561
Basil Std 0	Basil Std 0.D	QC	1	2/25/2016 9:31 PM		4.220	721	0.0207	0.6		316	44	3.993	34874
Basil Std 5	Basil Std 5.D	QC	2	2/25/2016 9:44 PM	5	4.220	5154	0.1022	3.6	72	2794	54	3.993	50421
Basil Std 10	Basil Std 10.D	QC	3	2/25/2016 9:58 PM	10	4.220	4666	0.1926	6.9	69	2605	56	3.993	24231
Basil Std 25	Basil Std 25.D	QC	4	2/25/2016 10:12 PM	25	4.220	13034	0.4457	16.1	64	7035	54	3.993	29245
Basil Std 50	Basil Std 50.D	QC	5	2/25/2016 10:26 PM	50	4.220	45050	0.9459	34.6	69	24691	55	3.993	47628
Basil Std 100	Basil Std 100.D	QC	6	2/25/2016 10:40 PM	100	4.220	63723	2.0961	78.4	78	34420	54	3.993	30401
Basil Std 150	Basil Std 150.D	QC	7	2/25/2016 10:54 PM	150	4.220	93056	2.3673	89.0	59	51270	55	3.993	39308
Carrot Spk 10a	Carrot Spk 10a.D	MatrixSpike		2/23/2016 9:50 PM		4.220	8411	0.2418	8.7		4732	56	3.993	34782
Carrot Spk 10b	Carrot Spk 10b.D	MatrixSpike		2/23/2016 10:03 PM		4.220	8604	0.2834	10.2		4638	54	3.993	30356
Carrot Spk 10c	Carrot Spk 10c.D	MatrixSpike		2/23/2016 10:17 PM		4.220	8612	0.2478	8.9		4674	54	3.993	34754
Carrot Spk 10d	Carrot Spk 10d.D	MatrixSpike		2/23/2016 10:31 PM		4.220	6581	0.2530	9.1		3588	55	3.993	26014

black circles = stds in solvent

blue triangles = matrix-matched stds

Compound Information

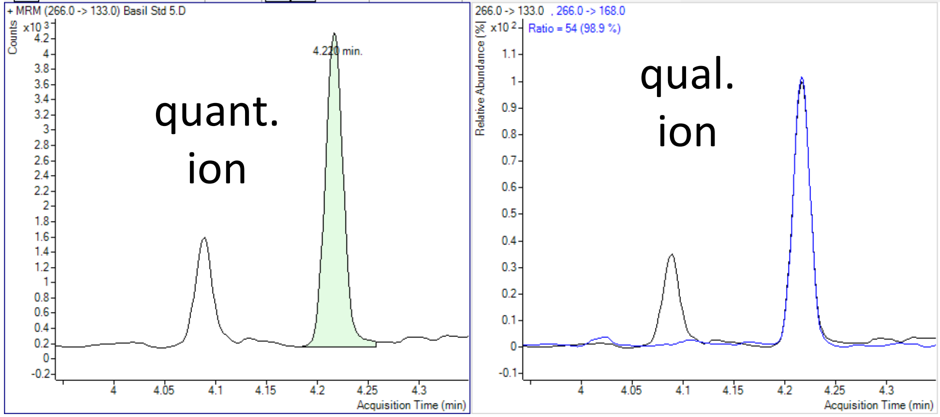

5 ng/mL std in basil

Calibration Curve

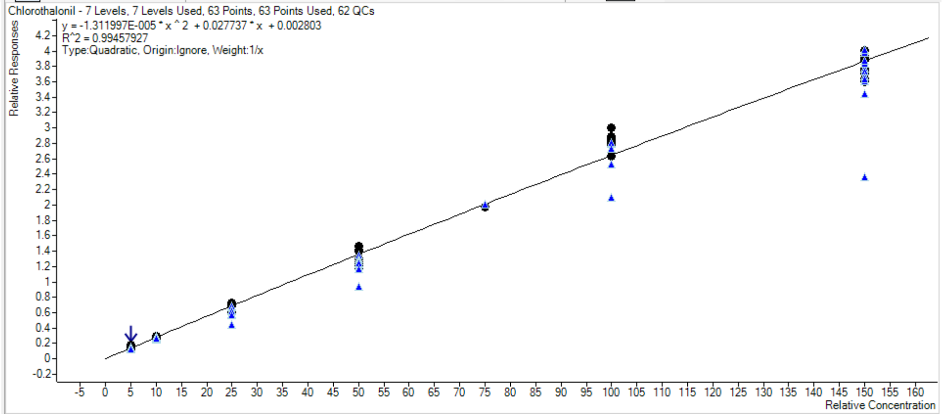

# Chlorothalonil 5 ng/mL calibration standards over the course of 5 days (inj. #)
RO = reagent-only; ion ratios within horizontal lines < |±10%| of ref. ratio

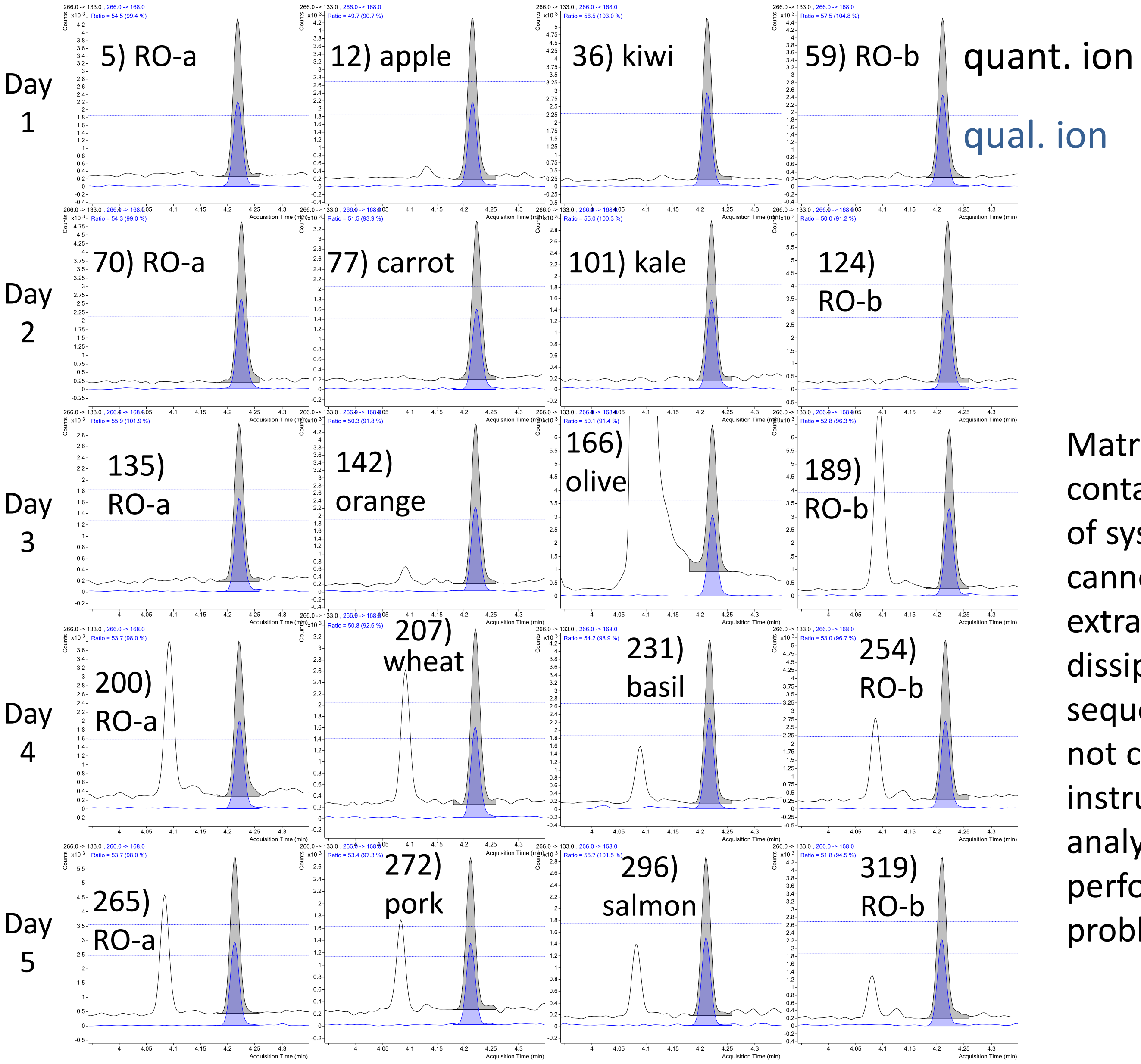

Matrix contamination of system from canned black olive extracts slowly dissipated during sequence, but did not cause instrument or analytical performance problems.

Chlorpyrifos Results (vs. atrazine-d5 IS) – all 325 analyses combined!

Sample: Olive Std 5 | Sample Type: <All> | Compound: Chlorpyrifos | ISTD: Atrazine-d5

Sample					Chlorpyrif...	Chlorpyrifos Results					Qualifier (314...		Atrazine-d5 (I...	
Name	Data File	Type	Level	Acq. Date-Time	Exp. Conc.	RT	Area	ISTD Resp. Ratio	Calc. Conc.	Accuracy	Area	Ratio	RT	Area
Olive Spk 10b	Olive Spk 10b.D	MatrixSpike		2/24/2016 8:50 PM		4.581	13142	0.6518	15.6		7212	55	3.993	20164
Olive Spk 10c	Olive Spk 10c.D	MatrixSpike		2/24/2016 9:04 PM		4.581	10643	0.6344	15.1		6159	58	3.993	16776
Olive Spk 10d	Olive Spk 10d.D	MatrixSpike		2/24/2016 9:18 PM		4.581	18842	0.5844	13.9		10935	58	3.993	32241
Olive Spk 25a	Olive Spk 25a.D	MatrixSpike		2/24/2016 9:31 PM		4.581	26197	1.2132	29.4		14830	57	3.993	21594
Olive Spk 25b	Olive Spk 25b.D	MatrixSpike		2/24/2016 9:45 PM		4.581	22841	1.2178	29.6		12711	56	3.993	18755
Olive Spk 25c	Olive Spk 25c.D	MatrixSpike		2/24/2016 9:59 PM		4.581	31552	1.2570	30.5		18071	57	3.993	25102
Olive Spk 25d	Olive Spk 25d.D	MatrixSpike		2/24/2016 10:13 PM		4.581	29191	1.2037	29.2		17240	59	3.993	24251
Olive Spk 50a	Olive Spk 50a.D	MatrixSpike		2/24/2016 10:27 PM		4.581	83677	2.5228	61.3		48680	58	3.993	33168
Olive Spk 50b	Olive Spk 50b.D	MatrixSpike		2/24/2016 10:41 PM		4.581	91894	2.6533	64.4		51790	56	3.993	34634
Olive Spk 50c	Olive Spk 50c.D	MatrixSpike		2/24/2016 10:54 PM		4.581	84009	2.4850	60.4		48478	58	3.993	33806
Olive Spk 50d	Olive Spk 50d.D	MatrixSpike		2/24/2016 11:08 PM		4.581	88075	2.4645	59.9		50693	58	3.993	35737
Olive Spk 100a	Olive Spk 100a.D	MatrixSpike		2/24/2016 11:22 PM		4.581	99109	5.5373	132.0		56528	57	3.993	17898
Olive Spk 100b	Olive Spk 100b.D	MatrixSpike		2/24/2016 11:36 PM		4.581	102493	5.2609	125.6		58862	57	3.993	19482
Olive Spk 100c	Olive Spk 100c.D	MatrixSpike		2/24/2016 11:50 PM		4.581	182878	5.4194	129.3		104877	57	3.993	33745
Olive Spk 100d	Olive Spk 100d.D	MatrixSpike		2/25/2016 12:03 AM		4.581	125585	5.2857	126.2		72845	58	3.993	23759
Olive Std 0	Olive Std 0.D	QC	1	2/24/2016 7:00 PM		4.581	1724	0.0446	0.4		931	54	3.993	38645
Olive Std 5	Olive Std 5.D	QC	2	2/24/2016 7:13 PM	5	4.581	10796	0.2429	5.4	107	6426	60	3.993	44449
Olive Std 10	Olive Std 10.D	QC	3	2/24/2016 7:27 PM	10	4.581	19941	0.4221	9.8	98	11320	57	3.993	47245
Olive Std 25	Olive Std 25.D	QC	4	2/24/2016 7:41 PM	25	4.581	32157	1.0093	24.4	98	18565	58	3.993	31862
Olive Std 50	Olive Std 50.D	QC	5	2/24/2016 7:55 PM	50	4.581	80754	1.9836	48.3	97	46195	57	3.993	40710
Olive Std 100	Olive Std 100.D	QC	6	2/24/2016 8:09 PM	100	4.581	112098	4.3465	104.5	104	64399	57	3.993	25791
Olive Std 150	Olive Std 150.D	QC	7	2/24/2016 8:22 PM	150	4.581	158235	6.2710	148.7	99	90832	57	3.993	25233
Orange Spk 10a	Orange Spk 10a.D	MatrixSpike		2/24/2016 3:05 PM		4.581	15442	0.5706	13.5		8836	57	3.993	27066
Orange Spk 10b	Orange Spk 10b.D	MatrixSpike		2/24/2016 3:19 PM		4.581	15587	0.5619	13.3		8925	57	3.993	27740

black circles = stds in solvent

blue triangles = matrix-matched stds

Compound Information

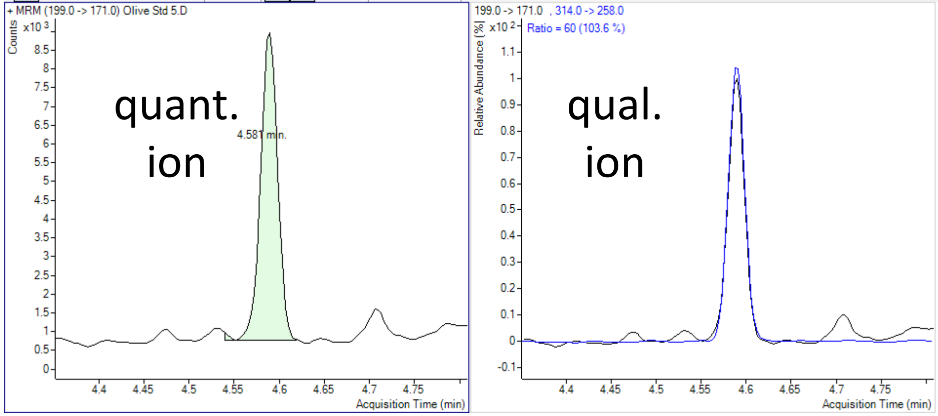

Calibration Curve

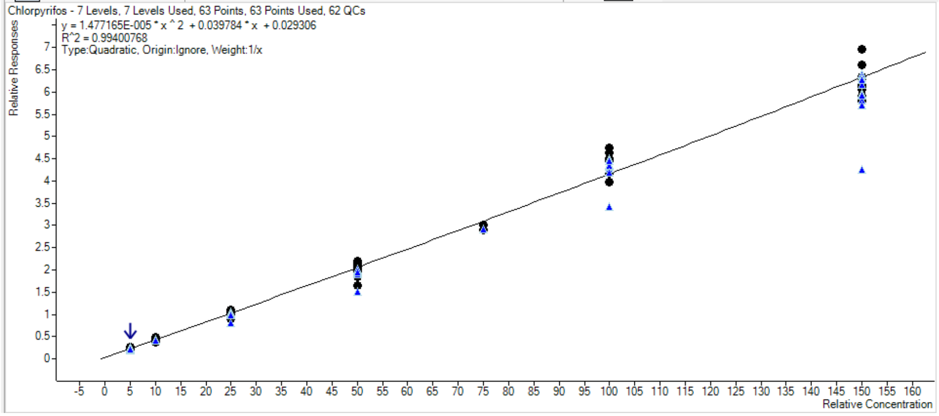

5 ng/mL std in black olive

Cyprodinil Results (vs. atrazine-d5 IS) – all 325 analyses combined!

Sample: Olive Std 5 | Sample Type: <All> | Compound: Cyprodinil | ISTD: Atrazine-d5

Sample						Cyprodini...	Cyprodinil Results					Qualifier (224...		Atrazine-d5 (I...	
		Name	Data File	Type	Level	Acq. Date-Time	Exp. Conc.	RT	Area	ISTD Resp. Ratio	Calc. Conc.	Accuracy	Area	Ratio	RT	Area
		Olive Spk 10b	Olive Spk 10b.D	MatrixSpike		2/24/2016 8:50 PM		4.712	15045	0.7461	11.9		3964	26	3.993	20164
		Olive Spk 10c	Olive Spk 10c.D	MatrixSpike		2/24/2016 9:04 PM		4.712	15421	0.9192	14.8		3686	24	3.993	16776
		Olive Spk 10d	Olive Spk 10d.D	MatrixSpike		2/24/2016 9:18 PM		4.712	31782	0.9858	15.9		7058	22	3.993	32241
		Olive Spk 25a	Olive Spk 25a.D	MatrixSpike		2/24/2016 9:31 PM		4.712	34924	1.6173	26.5		9328	27	3.993	21594
		Olive Spk 25b	Olive Spk 25b.D	MatrixSpike		2/24/2016 9:45 PM		4.712	31881	1.6998	27.8		8155	26	3.993	18755
		Olive Spk 25c	Olive Spk 25c.D	MatrixSpike		2/24/2016 9:59 PM		4.712	43750	1.7429	28.5		10022	23	3.993	25102
		Olive Spk 25d	Olive Spk 25d.D	MatrixSpike		2/24/2016 10:13 PM		4.712	39633	1.6343	26.7		9939	25	3.993	24251
		Olive Spk 50a	Olive Spk 50a.D	MatrixSpike		2/24/2016 10:27 PM		4.712	128552	3.8758	63.9		30847	24	3.993	33168
		Olive Spk 50b	Olive Spk 50b.D	MatrixSpike		2/24/2016 10:41 PM		4.712	137535	3.9711	65.5		34116	25	3.993	34634
		Olive Spk 50c	Olive Spk 50c.D	MatrixSpike		2/24/2016 10:54 PM		4.712	128431	3.7990	62.7		33265	26	3.993	33806
		Olive Spk 50d	Olive Spk 50d.D	MatrixSpike		2/24/2016 11:08 PM		4.712	132762	3.7150	61.3		32236	24	3.993	35737
		Olive Spk 100a	Olive Spk 100a.D	MatrixSpike		2/24/2016 11:22 PM		4.712	136126	7.6055	125.1		36175	27	3.993	17898
		Olive Spk 100b	Olive Spk 100b.D	MatrixSpike		2/24/2016 11:36 PM		4.712	143182	7.3494	121.0		38551	27	3.993	19482
		Olive Spk 100c	Olive Spk 100c.D	MatrixSpike		2/24/2016 11:50 PM		4.712	259339	7.6852	126.4		68497	26	3.993	33745
		Olive Spk 100d	Olive Spk 100d.D	MatrixSpike		2/25/2016 12:03 AM		4.712	172235	7.2491	119.3		45290	26	3.993	23759
		Olive Std 0	Olive Std 0.D	QC	1	2/24/2016 7:00 PM		4.712	2825	0.0731	0.7		486	17	3.993	38645
		Olive Std 5	Olive Std 5.D	QC	2	2/24/2016 7:13 PM	5	4.712	18602	0.4185	6.4	129	4052	22	3.993	44449
		Olive Std 10	Olive Std 10.D	QC	3	2/24/2016 7:27 PM	10	4.712	39485	0.8358	13.4	134	8188	21	3.993	47245
		Olive Std 25	Olive Std 25.D	QC	4	2/24/2016 7:41 PM	25	4.712	45367	1.4239	23.2	93	11668	26	3.993	31862
		Olive Std 50	Olive Std 50.D	QC	5	2/24/2016 7:55 PM	50	4.712	119264	2.9296	48.3	97	30805	26	3.993	40710
		Olive Std 100	Olive Std 100.D	QC	6	2/24/2016 8:09 PM	100	4.712	167590	6.4981	107.1	107	43999	26	3.993	25791
		Olive Std 150	Olive Std 150.D	QC	7	2/24/2016 8:22 PM	150	4.712	237108	9.3968	154.3	103	62286	26	3.993	25233
		Orange Spk 10a	Orange Spk 10a.D	MatrixSpike		2/24/2016 3:05 PM		4.712	17739	0.6554	10.4		5026	28	3.993	27066
		Orange Spk 10b	Orange Spk 10b.D	MatrixSpike		2/24/2016 3:19 PM		4.712	19142	0.6900	11.0		5033	26	3.993	27740

black circles = stds in solvent

blue triangles = matrix-matched stds

Compound Information

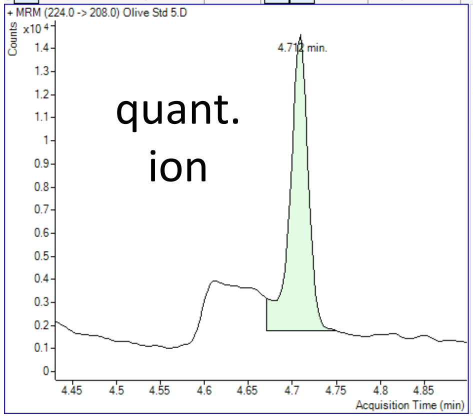

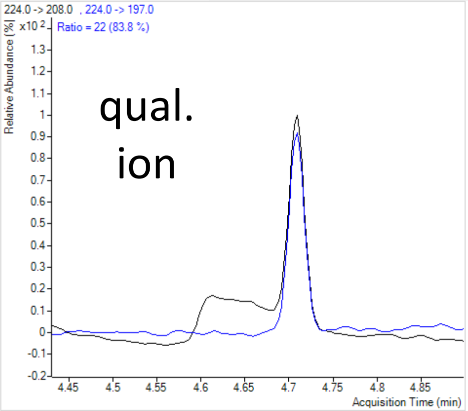

Calibration Curve

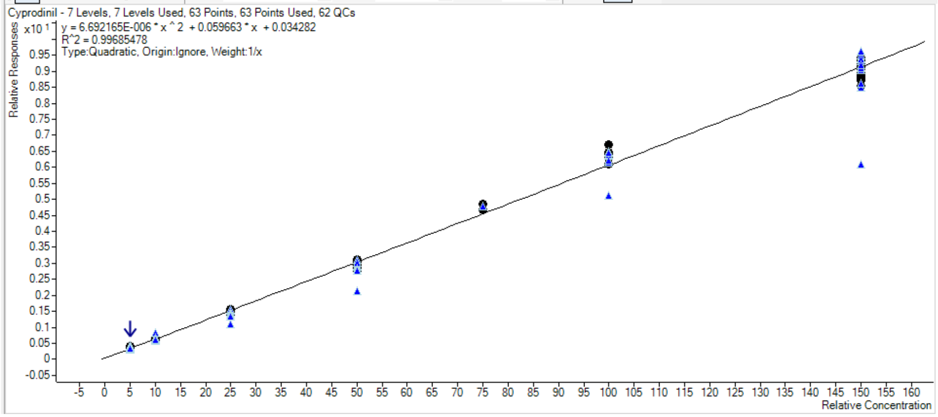

5 ng/mL std in black olive

TBECH Results (vs. atrazine-d5) – all 325 analyses combined!

Sample: Wheat Std 10 | Sample Type: <All> | Compound: TBECH | ISTD: Atrazine-d5

Sample							TBECH ...	TBECH Results							Qualifier (267....		Atrazine-d5 (I...	
Name	Data File	Type	Level	Acq. Date-Time	Vol.		Exp. Conc.	RT	Resp.	Calc. Conc.	Final Conc.	Accuracy	S/N	Area	Ratio	Area	RT	Resp.
SO Std 25a WB	SO Std 25a WB.D	Cal	4	2/25/2016 3:04 PM	1.00		25.0000	4.705	2626	22.6672	22.6672	90.7	4.17	2626	130.5	3426	3.993	24081
SO Std 50a WB	SO Std 50a WB.D	Cal	5	2/25/2016 3:18 PM	1.00		50.0000	4.708	11459	55.5260	55.5260	111.1	31.64	11459	132.5	15188	3.993	41332
SO Std 100a WB	SO Std 100a WB.D	Cal	6	2/25/2016 3:31 PM	1.00		100.0000	4.708	14905	109.5649	109.5649	109.6	27.62	14905	138.8	20691	3.993	27206
SO Std 150a WB	SO Std 150a WB.D	Cal	7	2/25/2016 3:45 PM	1.00		150.0000	4.705	22344	139.4471	139.4471	93.0	153....	22344	128.6	28735	3.993	32185
Wheat Std 0	Wheat Std 0.D	QC	1	2/25/2016 3:59 PM	1.00			4.720	96	2.3968	2.3968		0.67	96	110.9	107	3.993	24540
Wheat Std 5	Wheat Std 5.D	QC	2	2/25/2016 4:13 PM	1.00		5.0000	4.700	349	4.4524	4.4524	89.0	2.69	349	288.3	1007	3.993	23867
Wheat Std 10	Wheat Std 10.D	QC	3	2/25/2016 4:27 PM	1.00		10.0000	4.711	1672	9.4741	9.4741	94.7	3.79	1672	125.1	2092	3.993	41019
Wheat Std 25	Wheat Std 25.D	QC	4	2/25/2016 4:40 PM	1.00		25.0000	4.702	2412	20.5004	20.5004	82.0	4.65	2412	144.4	3484	3.993	24647
Wheat Std 50	Wheat Std 50.D	QC	5	2/25/2016 4:54 PM	1.00		50.0000	4.708	11096	50.8192	50.8192	101.6	95.30	11096	129.9	14410	3.993	43802
Wheat Std 100	Wheat Std 100.D	QC	6	2/25/2016 5:08 PM	1.00		100.0000	4.702	20736	108.8737	108.8737	108.9	92.62	20736	125.5	26017	3.993	38089
Wheat Std 150	Wheat Std 150.D	QC	7	2/25/2016 5:22 PM	1.00		150.0000	4.702	29532	142.9134	142.9134	95.3	Infinity	29532	127.3	37602	3.993	41532
Wheat Spk 10a	Wheat Spk 10a.D	MatrixSpike		2/25/2016 5:36 PM	1.00			4.711	1177	10.6475	10.6475		6.30	1177	99.9	1176	3.993	25122
Wheat Spk 10b	Wheat Spk 10b.D	MatrixSpike		2/25/2016 5:50 PM	1.00			4.711	1886	12.5524	12.5524		10.03	1886	92.8	1751	3.993	33246
Wheat Spk 10c	Wheat Spk 10c.D	MatrixSpike		2/25/2016 6:03 PM	1.00			4.700	1511	9.0429	9.0429		10.82	1511	114.6	1732	3.993	39230
Wheat Spk 10d	Wheat Spk 10d.D	MatrixSpike		2/25/2016 6:17 PM	1.00			4.700	1295	9.8638	9.8638		5.96	1295	134.9	1747	3.993	30280
Wheat Spk 25a	Wheat Spk 25a.D	MatrixSpike		2/25/2016 6:31 PM	1.00			4.702	3210	19.9438	19.9438		5.23	3210	115.8	3717	3.993	33795
Wheat Spk 25b	Wheat Spk 25b.D	MatrixSpike		2/25/2016 6:45 PM	1.00			4.700	4725	23.8700	23.8700		11.53	4725	129.2	6103	3.993	40991
Wheat Spk 25c	Wheat Spk 25c.D	MatrixSpike		2/25/2016 6:59 PM	1.00			4.702	2614	22.7772	22.7772		9.40	2614	115.6	3020	3.993	23843
Wheat Spk 25d	Wheat Spk 25d.D	MatrixSpike		2/25/2016 7:12 PM	1.00			4.702	2836	23.6420	23.6420		7.78	2836	130.8	3711	3.993	24861
Wheat Spk 50a	Wheat Spk 50a.D	MatrixSpike		2/25/2016 7:26 PM	1.00			4.702	9368	46.0295	46.0295		17.74	9368	136.9	12826	3.993	40921
Wheat Spk 50b	Wheat Spk 50b.D	MatrixSpike		2/25/2016 7:40 PM	1.00			4.708	4249	38.9866	38.9866		21.70	4249	147.1	6252	3.993	22021
Wheat Spk 50c	Wheat Spk 50c.D	MatrixSpike		2/25/2016 7:54 PM	1.00			4.705	5964	41.0246	41.0246		38.23	5964	125.7	7498	3.993	29327
Wheat Spk 50d	Wheat Spk 50d.D	MatrixSpike		2/25/2016 8:08 PM	1.00			4.697	6254	40.3694	40.3694		54.62	6254	129.6	8108	3.993	31270
Wheat Spk 100a	Wheat Spk 100a.D	MatrixSpike		2/25/2016 8:22 PM	1.00			4.708	21917	102.2971	102.2971		17.49	21917	133.9	29353	3.993	42817

black circles = stds in solvent

blue triangles = matrix-matched stds

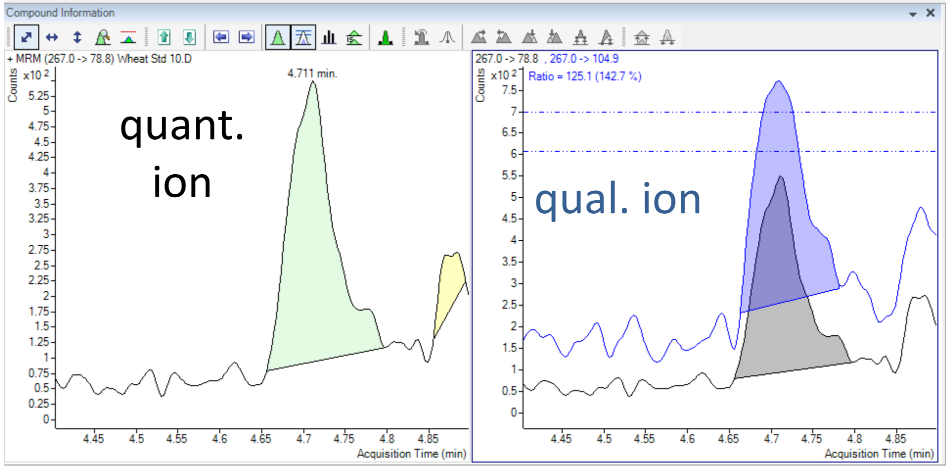

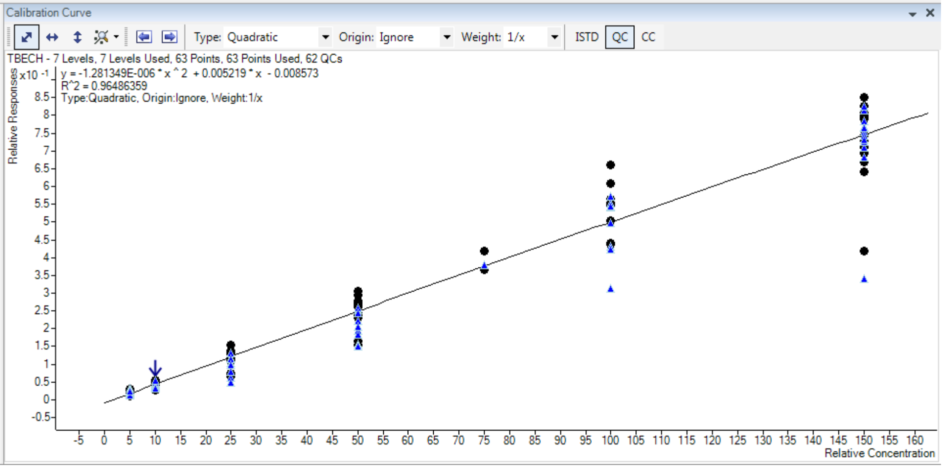

10 ng/mL in wheat grain

Thiabendazole Results (vs. atrazine-d5) – all 325 analyses combined!

Sample: Salmon Std 5 | Sample Type: <All> | Compound: Thiabendazole | ISTD: Atrazine-d5

		Sample						Thiabend...	Thiabendazole Results							Qualifier (174...		Atrazine-d5 (I...	
!	⚑	Name	Data File	Type	Level	Acq. Date-Time	Vol.	Exp. Conc.	RT	Resp.	Calc. Conc.	Final Conc.	Accuracy	S/N	Area	Ratio	Area	RT	Resp.
	⚑	Pork Spk 10c	Pork Spk 10c.D	MatrixSpike		2/26/2016 1:48 PM	1.00		4.939	16926	4.8882	4.8882		Infinity	16926	22.3	3772	3.993	26710
	⚑	Pork Spk 10d	Pork Spk 10d.D	MatrixSpike		2/26/2016 2:02 PM	1.00		4.939	20816	4.1830	4.1830		Infinity	20816	25.0	5197	3.993	37301
		Pork Spk 25a	Pork Spk 25a.D	MatrixSpike		2/26/2016 2:16 PM	1.00		4.939	31536	10.8879	10.8879		Infinity	31536	20.1	6343	3.993	24673
		Pork Spk 25b	Pork Spk 25b.D	MatrixSpike		2/26/2016 2:29 PM	1.00		4.939	34973	12.7357	12.7357		Infinity	34973	19.5	6820	3.993	23680
		Pork Spk 25c	Pork Spk 25c.D	MatrixSpike		2/26/2016 2:43 PM	1.00		4.939	39499	12.2723	12.2723		Infinity	39499	19.7	7780	3.993	27678
		Pork Spk 25d	Pork Spk 25d.D	MatrixSpike		2/26/2016 2:57 PM	1.00		4.939	42438	11.7816	11.7816		Infinity	42438	18.2	7725	3.993	30880
		Pork Spk 50a	Pork Spk 50a.D	MatrixSpike		2/26/2016 3:11 PM	1.00		4.939	82791	24.6405	24.6405		Infinity	82791	19.1	15795	3.993	29986
		Pork Spk 50b	Pork Spk 50b.D	MatrixSpike		2/26/2016 3:25 PM	1.00		4.939	81420	22.7259	22.7259		Infinity	81420	19.5	15888	3.993	31878
		Pork Spk 50c	Pork Spk 50c.D	MatrixSpike		2/26/2016 3:38 PM	1.00		4.939	151193	30.1393	30.1393		Infinity	151193	19.4	29347	3.993	45051
		Pork Spk 50d	Pork Spk 50d.D	MatrixSpike		2/26/2016 3:52 PM	1.00		4.939	83141	25.7975	25.7975		Infinity	83141	18.4	15300	3.993	28808
		Pork Spk 100a	Pork Spk 100a.D	MatrixSpike		2/26/2016 4:06 PM	1.00		4.939	226473	51.5701	51.5701		Infinity	226473	19.1	43200	3.993	39823
		Pork Spk 100b	Pork Spk 100b.D	MatrixSpike		2/26/2016 4:20 PM	1.00		4.939	220809	55.1363	55.1363		Infinity	220809	18.8	41554	3.993	36337
		Pork Spk 100c	Pork Spk 100c.D	MatrixSpike		2/26/2016 4:34 PM	1.00		4.939	116938	46.8791	46.8791		Infinity	116938	18.6	21772	3.993	22596
		Pork Spk 100d	Pork Spk 100d.D	MatrixSpike		2/26/2016 4:48 PM	1.00		4.939	236634	59.6306	59.6306		Infinity	236634	18.9	44788	3.993	36025
!	⚑	MeCN b PS	MeCN b PS.D	Blank		2/26/2016 5:02 PM	1.00		4.939	753	50.6224	50.6224		Infinity	753	76.5	576	3.993	135
!	⚑	Salmon Std 0	Salmon Std 0.D	QC	1	2/26/2016 5:15 PM	1.00		4.939	4680	1.0071	1.0071		Infinity	4680	35.0	1637	3.993	21507
		Salmon Std 5	Salmon Std 5.D	QC	2	2/26/2016 5:29 PM	1.00	5.0000	4.939	16081	4.9901	4.9901	99.8	Infinity	16081	26.4	4240	3.993	24945
		Salmon Std 10	Salmon Std 10.D	QC	3	2/26/2016 5:43 PM	1.00	10.0000	4.939	30673	9.6881	9.6881	96.9	Infinity	30673	20.5	6281	3.993	26691
		Salmon Std 25	Salmon Std 25.D	QC	4	2/26/2016 5:57 PM	1.00	25.0000	4.939	73364	25.5773	25.5773	102.3	Infinity	73364	19.2	14060	3.993	25632
		Salmon Std 50	Salmon Std 50.D	QC	5	2/26/2016 6:11 PM	1.00	50.0000	4.939	132996	50.8840	50.8840	101.8	Infinity	132996	18.8	25060	3.993	23698
		Salmon Std 100	Salmon Std 100.D	QC	6	2/26/2016 6:24 PM	1.00	100.0000	4.939	279508	115.1071	115.1071	115.1	Infinity	279508	18.9	52778	3.993	21994
!		Salmon Std 150	Salmon Std 150.D	QC	7	2/26/2016 6:38 PM	1.00	150.0000	4.939	483889	157.6043	157.6043	105.1	Infinity	483889	19.1	92396	3.993	27655
		Salmon Spk 10a	Salmon Spk 10a.D	MatrixSpike		2/26/2016 6:52 PM	1.00		4.939	27713	8.2578	8.2578		Infinity	27713	21.2	5870	3.993	27839
!		Salmon Spk 10b	Salmon Spk 10b.D	MatrixSpike		2/26/2016 7:06 PM	1.00		4.939	22044	6.2678	6.2678		Infinity	22044	22.7	5009	3.993	28197

black circles = stds in solvent
blue triangles = matrix-matched stds

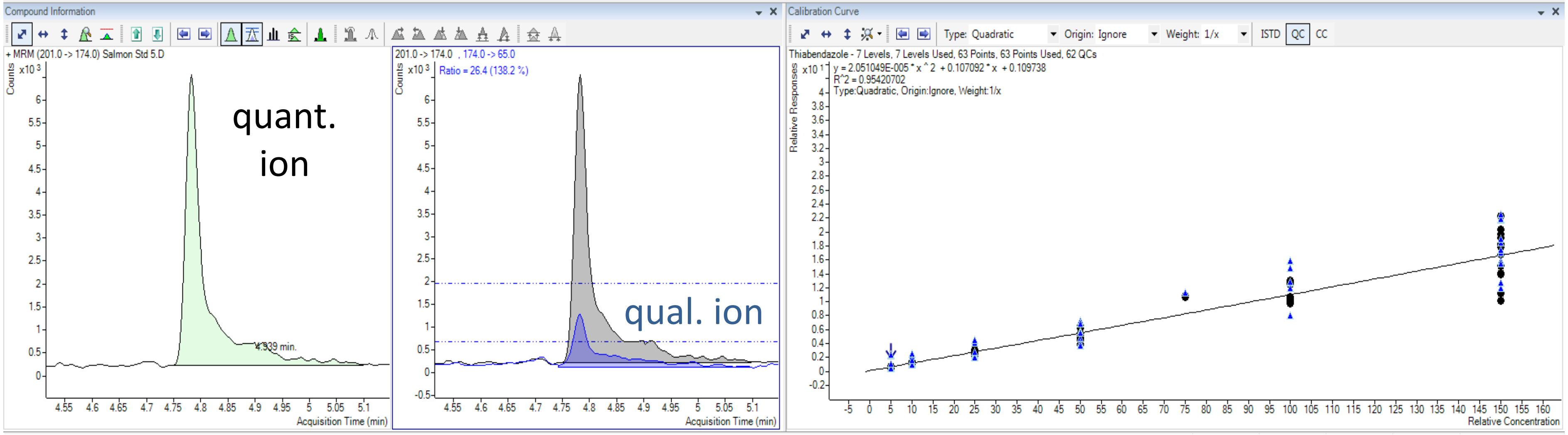

5 ng/mL in salmon

Flutriafol Results (vs. atrazine-d5 IS) – all 325 analyses combined!

Sample: Kale Std 5 | Sample Type: <All> | Compound: Flutriafol | ISTD: Atrazine-d5

Sample					Flutriafol...	Flutriafol Results					Qualifier (219...		Atrazine-d5 (I...	
Name	Data File	Type	Level	Acq. Date-Time	Exp. Conc.	RT	Area	ISTD Resp. Ratio	Calc. Conc.	Accuracy	Area	Ratio	RT	Area
Kale Spk 25b	Kale Spk 25b.D	MatrixSpike		2/24/2016 4:30 AM		4.952	24648	0.7211	10.1		21780	88	3.993	34180
Kale Spk 25c	Kale Spk 25c.D	MatrixSpike		2/24/2016 4:44 AM		4.952	100952	3.0072	43.3		89345	89	3.993	33570
Kale Spk 25d	Kale Spk 25d.D	MatrixSpike		2/24/2016 4:58 AM		4.954	63512	1.6604	23.7		56603	89	3.993	38251
Kale Spk 50a	Kale Spk 50a.D	MatrixSpike		2/24/2016 5:11 AM		4.952	65460	1.7387	24.8		56839	87	3.993	37648
Kale Spk 50b	Kale Spk 50b.D	MatrixSpike		2/24/2016 5:25 AM		4.954	126653	3.4267	49.5		111594	88	3.993	36961
Kale Spk 50c	Kale Spk 50c.D	MatrixSpike		2/24/2016 5:39 AM		4.952	84876	3.1274	45.1		74966	88	3.993	27140
Kale Spk 50d	Kale Spk 50d.D	MatrixSpike		2/24/2016 5:53 AM		4.952	102213	3.1383	45.3		89637	88	3.993	32569
Kale Spk 100a	Kale Spk 100a.D	MatrixSpike		2/24/2016 6:07 AM		4.954	194351	7.0989	103.5		170291	88	3.993	27378
Kale Spk 100b	Kale Spk 100b.D	MatrixSpike		2/24/2016 6:20 AM		4.952	157548	6.8159	99.3		137751	87	3.993	23115
Kale Spk 100c	Kale Spk 100c.D	MatrixSpike		2/24/2016 6:34 AM		4.954	190330	7.0279	102.5		166111	87	3.993	27082
Kale Spk 100d	Kale Spk 100d.D	MatrixSpike		2/24/2016 6:48 AM		4.952	214727	6.3744	92.8		189743	88	3.993	33686
Kale Std 0	Kale Std 0.D	QC	1	2/24/2016 1:44 AM		4.952	1519	0.0624	0.5		1260	83	3.993	24333
Kale Std 5	Kale Std 5.D	QC	2	2/24/2016 1:58 AM	5	4.954	11797	0.4410	6.0	120	10361	88	3.993	26751
Kale Std 10	Kale Std 10.D	QC	3	2/24/2016 2:12 AM	10	4.952	23288	0.7712	10.8	108	19820	85	3.993	30199
Kale Std 25	Kale Std 25.D	QC	4	2/24/2016 2:26 AM	25	4.952	106968	1.5175	21.6	87	94423	88	3.993	70490
Kale Std 50	Kale Std 50.D	QC	5	2/24/2016 2:40 AM	50	4.954	139295	3.6621	52.9	106	121726	87	3.993	38037
Kale Std 100	Kale Std 100.D	QC	6	2/24/2016 2:53 AM	100	4.952	213488	7.6085	111.1	111	187977	88	3.993	28059
Kale Std 150	Kale Std 150.D	QC	7	2/24/2016 3:07 AM	150	4.954	269763	10.6318	156.2	104	237892	88	3.993	25373
Kiwi Spk 10a	Kiwi Spk 10a.D	MatrixSpike		2/23/2016 4:43 AM		4.952	27086	0.7021	9.8		24014	89	3.993	38579
Kiwi Spk 10b	Kiwi Spk 10b.D	MatrixSpike		2/23/2016 4:58 AM		4.954	26625	0.7313	10.2		23722	89	3.993	36410
Kiwi Spk 10c	Kiwi Spk 10c.D	MatrixSpike		2/23/2016 5:13 AM		4.952	21915	0.7217	10.1		19470	89	3.993	30368
Kiwi Spk 10d	Kiwi Spk 10d.D	MatrixSpike		2/23/2016 5:29 AM		4.954	29624	0.7867	11.0		26075	88	3.993	37655
Kiwi Spk 25a	Kiwi Spk 25a.D	MatrixSpike		2/23/2016 5:44 AM		4.952	60840	1.7045	24.3		52622	86	3.993	35693
Kiwi Spk 25b	Kiwi Spk 25b.D	MatrixSpike		2/23/2016 5:59 AM		4.954	69089	1.8132	25.9		61302	89	3.993	38103

black circles = stds in solvent

blue triangles = matrix-matched stds

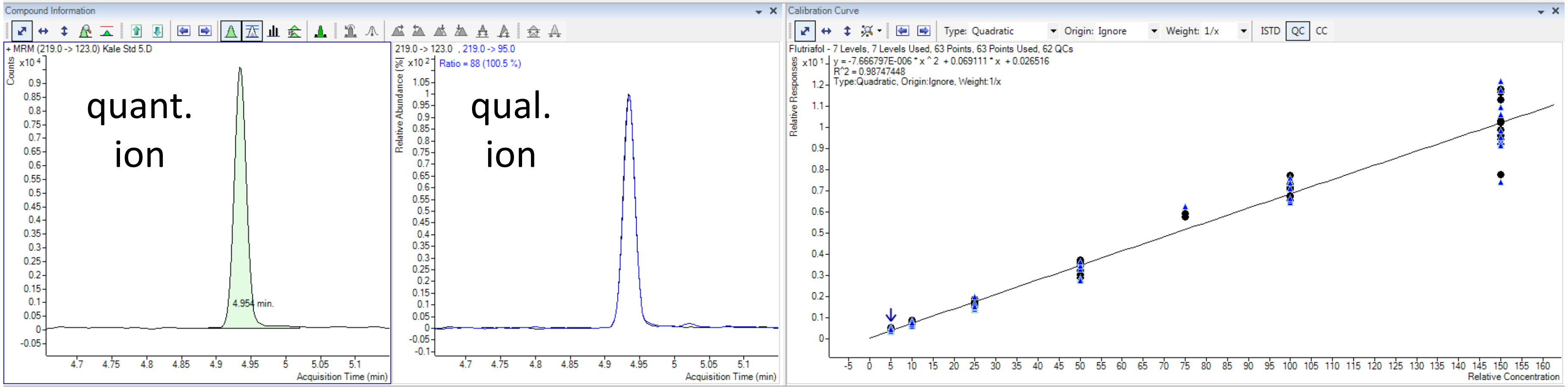

5 ng/mL std in kale

Imazalil Results (vs. atrazine-d5) – all 325 analyses combined!

Sample: Apple Std 5 | Sample Type: <All> | Compound: Imazalil | ISTD: Atrazine-d5

Sample						Imazalil...	Imazalil Results							Qualifier (215...		Atrazine-d5 (I...	
Name	Data File	Type	Level	Acq. Date-Time	Vol.	Exp. Conc.	RT	Resp.	Calc. Conc.	Final Conc.	Accuracy	S/N	Area	Ratio	Area	RT	Resp.
SO Std 150 AK	SO Std 150 AK.D	Cal	7	2/22/2016 6:26 PM	1.00	150.0000	4.986	67657	170.9072	170.9072	113.9	Infinity	67657	23.1	15598	3.993	17409
MeCN a AK	MeCN a AK.D	Blank		2/22/2016 6:41 PM	1.00		4.986	1039	141.9139	141.9139		Infinity	1039	28.7	298	3.993	323
Rgt Blk AK	Rgt Blk AK.D	Blank		2/22/2016 6:56 PM	1.00		4.986	561	104.7403	104.7403		Infinity	561	62.9	353	3.993	238
SO Std 0a AK	SO Std 0a AK.D	Blank		2/22/2016 7:11 PM	1.00		4.986	582	0.8515	0.8515		Infinity	582	41.5	242	3.993	40363
SO Std 5a AK	SO Std 5a AK.D	Cal	2	2/22/2016 7:26 PM	1.00	5.0000	4.986	4199	6.0563	6.0563	121.1	Infinity	4199	27.9	1171	3.993	32218
SO Std 10a AK	SO Std 10a AK.D	Cal	3	2/22/2016 7:41 PM	1.00	10.0000	4.986	8697	10.9338	10.9338	109.3	Infinity	8697	22.3	1941	3.993	36369
SO Std 25a AK	SO Std 25a AK.D	Cal	4	2/22/2016 7:56 PM	1.00	25.0000	4.986	24268	27.3495	27.3495	109.4	Infinity	24268	23.2	5637	3.993	40027
SO Std 50a AK	SO Std 50a AK.D	Cal	5	2/22/2016 8:11 PM	1.00	50.0000	4.986	49476	58.7111	58.7111	117.4	Infinity	49476	23.5	11644	3.993	37704
SO Std 100a AK	SO Std 100a AK.D	Cal	8	2/22/2016 8:26 PM	1.00	75.0000	4.986	83558	97.3270	97.3270	129.8	Infinity	83558	21.4	17920	3.993	38160
SO Std 150a AK	SO Std 150a AK.D	Cal	7	2/22/2016 8:41 PM	1.00	150.0000	4.986	154804	198.5843	198.5843	132.4	Infinity	154804	22.0	34018	3.993	34150
Apple Std 0	Apple Std 0.D	QC	1	2/22/2016 8:56 PM	1.00		4.986	975	1.3566	1.3566		Infinity	975	41.1	401	3.993	37987
Apple Std 5	Apple Std 5.D	QC	2	2/22/2016 9:11 PM	1.00	5.0000	4.986	5024	6.5823	6.5823	131.6	Infinity	5024	28.0	1406	3.993	35363
Apple Std 10	Apple Std 10.D	QC	3	2/22/2016 9:26 PM	1.00	10.0000	4.986	10229	10.5287	10.5287	105.3	Infinity	10229	23.1	2363	3.993	44457
Apple Std 25	Apple Std 25.D	QC	4	2/22/2016 9:42 PM	1.00	25.0000	4.986	21937	27.0693	27.0693	108.3	Infinity	21937	21.7	4766	3.993	36562
Apple Std 50	Apple Std 50.D	QC	5	2/22/2016 9:57 PM	1.00	50.0000	4.986	40423	49.5670	49.5670	99.1	Infinity	40423	21.4	8670	3.993	36556
Apple Std 100	Apple Std 100.D	QC	8	2/22/2016 10:12 PM	1.00	75.0000	4.986	83470	91.0629	91.0629	121.4	Infinity	83470	21.2	17718	3.993	40783
Apple Std 150	Apple Std 150.D	QC	7	2/22/2016 10:27 PM	1.00	150.0000	4.986	185796	188.9310	188.9310	126.0	Infinity	185796	21.2	39397	3.993	43139
Apple Spk 10a	Apple Spk 10a.D	MatrixSpike		2/22/2016 10:42 PM	1.00		4.986	10569	10.1919	10.1919		Infinity	10569	26.2	2768	3.993	47486
Apple Spk 10b	Apple Spk 10b.D	MatrixSpike		2/22/2016 10:57 PM	1.00		4.986	8146	8.7945	8.7945		Infinity	8146	27.6	2250	3.993	42561
Apple Spk 10c	Apple Spk 10c.D	MatrixSpike		2/22/2016 11:12 PM	1.00		4.986	9660	9.3349	9.3349		Infinity	9660	26.7	2581	3.993	47483
Apple Spk 10d	Apple Spk 10d.D	MatrixSpike		2/22/2016 11:27 PM	1.00		4.986	10186	10.1485	10.1485		Infinity	10186	25.3	2577	3.993	45965
Apple Spk 25a	Apple Spk 25a.D	MatrixSpike		2/22/2016 11:42 PM	1.00		4.986	20326	22.8189	22.8189		Infinity	20326	22.2	4504	3.993	40267
Apple Spk 25b	Apple Spk 25b.D	MatrixSpike		2/22/2016 11:57 PM	1.00		4.986	21517	21.7513	21.7513		Infinity	21517	23.2	4981	3.993	44743
Apple Spk 25c	Apple Spk 25c.D	MatrixSpike		2/23/2016 12:12 AM	1.00		4.986	21151	22.7845	22.7845		Infinity	21151	21.4	4526	3.993	41965

black circles = stds in solvent

blue triangles = matrix-matched stds

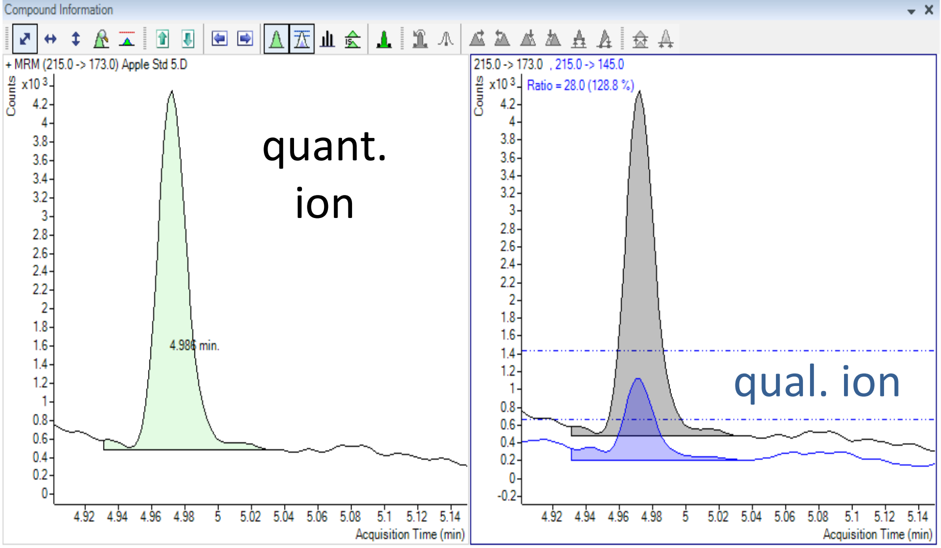

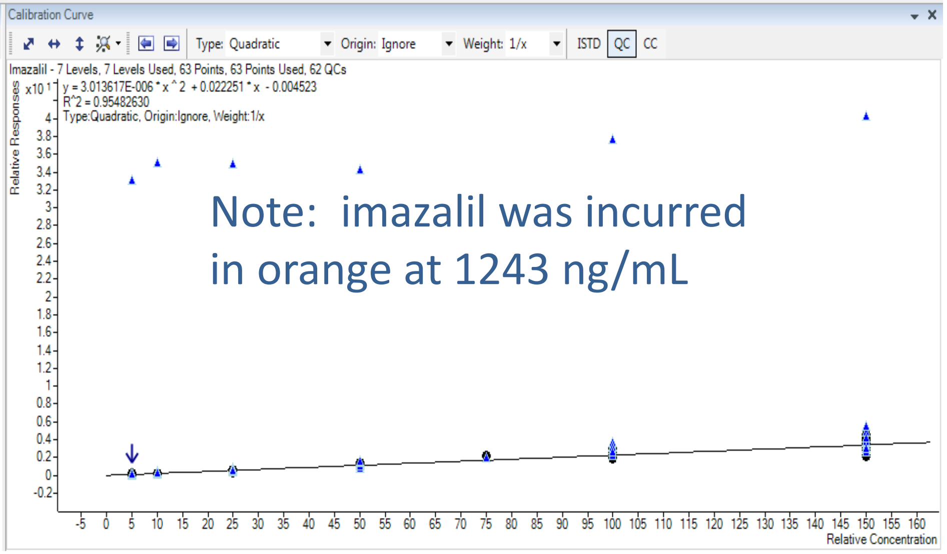

5 ng/mL std in apple

Myclobutanil Results (vs. atrazine-d5 IS) – all 325 analyses combined!

Sample: Apple Std 5 | Sample Type: <All> | Compound: Myclobutanil | ISTD: Atrazine-d5

Sample					Myclobut...	Myclobutanil Results					Qualifier (179...		Atrazine-d5 (I...	
Name	Data File	Type	Level	Acq. Date-Time	Exp. Conc.	RT	Area	ISTD Resp. Ratio	Calc. Conc.	Accuracy	Area	Ratio	RT	Area
Apple Spk 10a	Apple Spk 10a.D	MatrixSpike		2/22/2016 10:42 PM		5.016	54169	1.1407	11.1		29895	55	3.993	47486
Apple Spk 10b	Apple Spk 10b.D	MatrixSpike		2/22/2016 10:57 PM		5.016	45987	1.0805	10.4		25059	54	3.993	42561
Apple Spk 10c	Apple Spk 10c.D	MatrixSpike		2/22/2016 11:12 PM		5.016	51251	1.0794	10.4		28246	55	3.993	47483
Apple Spk 10d	Apple Spk 10d.D	MatrixSpike		2/22/2016 11:27 PM		5.016	52916	1.1512	11.2		28827	54	3.993	45965
Apple Spk 25a	Apple Spk 25a.D	MatrixSpike		2/22/2016 11:42 PM		5.016	104321	2.5907	25.5		56383	54	3.993	40267
Apple Spk 25b	Apple Spk 25b.D	MatrixSpike		2/22/2016 11:57 PM		5.016	117436	2.6247	25.9		63961	54	3.993	44743
Apple Spk 25c	Apple Spk 25c.D	MatrixSpike		2/23/2016 12:12 AM		5.016	111269	2.6515	26.1		60318	54	3.993	41965
Apple Spk 25d	Apple Spk 25d.D	MatrixSpike		2/23/2016 12:27 AM		5.016	113244	2.6845	26.5		62192	55	3.993	42185
Apple Spk 50a	Apple Spk 50a.D	MatrixSpike		2/23/2016 12:42 AM		5.016	204487	5.0392	49.6		111620	55	3.993	40579
Apple Spk 50b	Apple Spk 50b.D	MatrixSpike		2/23/2016 12:57 AM		5.016	172190	4.8783	48.0		93988	55	3.993	35298
Apple Spk 50c	Apple Spk 50c.D	MatrixSpike		2/23/2016 1:13 AM		5.016	150781	4.8035	47.3		81820	54	3.993	31390
Apple Spk 50d	Apple Spk 50d.D	MatrixSpike		2/23/2016 1:28 AM		5.016	211374	4.8138	47.4		115200	55	3.993	43910
Apple Spk 100a	Apple Spk 100a.D	MatrixSpike		2/23/2016 1:43 AM		5.016	445676	11.2499	108.9		242664	54	3.993	39616
Apple Spk 100b	Apple Spk 100b.D	MatrixSpike		2/23/2016 1:58 AM		5.016	411005	10.8567	105.2		222137	54	3.993	37857
Apple Spk 100c	Apple Spk 100c.D	MatrixSpike		2/23/2016 2:13 AM		5.016	552201	11.5877	112.0		301094	55	3.993	47654
Apple Spk 100d	Apple Spk 100d.D	MatrixSpike		2/23/2016 2:28 AM		5.016	496913	11.7729	113.8		272073	55	3.993	42208
Apple Std 0	Apple Std 0.D	QC	1	2/22/2016 8:56 PM		5.016	5620	0.1479	1.0		3180	57	3.993	37987
Apple Std 5	Apple Std 5.D	QC	2	2/22/2016 9:11 PM	5	5.016	24608	0.6959	6.6	132	13646	55	3.993	35363
Apple Std 10	Apple Std 10.D	QC	3	2/22/2016 9:26 PM	10	5.016	48736	1.0962	10.6	106	26208	54	3.993	44457
Apple Std 25	Apple Std 25.D	QC	4	2/22/2016 9:42 PM	25	5.016	100522	2.7494	27.1	108	56005	56	3.993	36562
Apple Std 50	Apple Std 50.D	QC	5	2/22/2016 9:57 PM	50	5.016	184761	5.0542	49.8	100	100667	54	3.993	36556
Apple Std 100	Apple Std 100.D	QC	8	2/22/2016 10:12 PM	75	5.016	350386	8.5916	83.8	112	192994	55	3.993	40783
Apple Std 150	Apple Std 150.D	QC	7	2/22/2016 10:27 PM	150	5.016	780333	18.0889	171.4	114	425136	54	3.993	43139
Basil Spk 10a	Basil Spk 10a.D	MatrixSpike		2/25/2016 11:07 PM		5.016	49037	1.1701	11.3		26888	55	3.993	41909

black circles = stds in solvent

blue triangles = matrix-matched stds

Compound Information

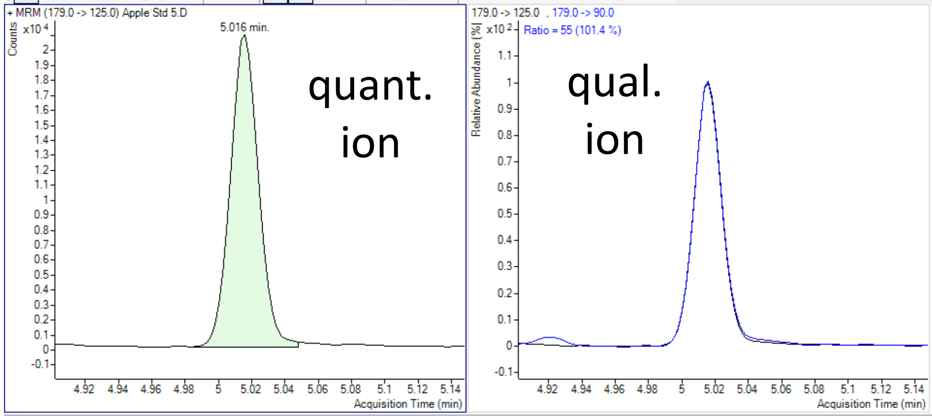

Calibration Curve

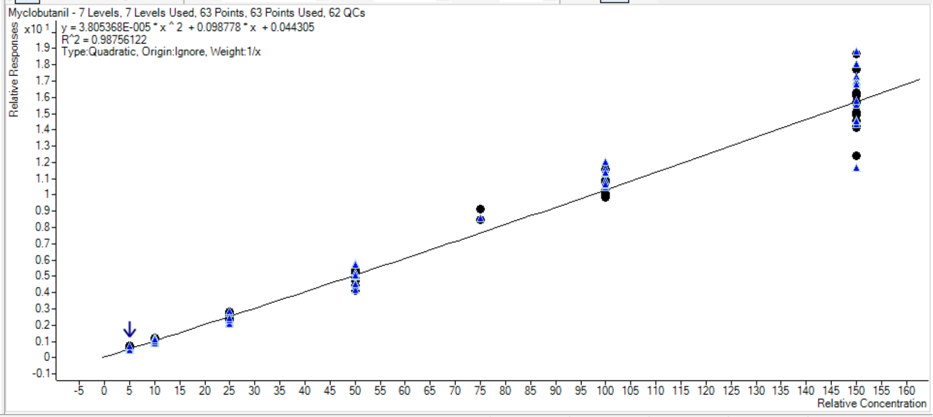

5 ng/mL std in apple

Endosulfan II Results (vs. atrazine-d5 IS) – all 325 analyses combined!

Sample: Apple Std 5 | Sample Type: <All> | Compound: Endosulfan II | ISTD: Atrazine-d5

Sample					Endosulf...	Endosulfan II Results					Qualifier (241...		Atrazine-d5 (I...	
Name	Data File	Type	Level	Acq. Date-Time	Exp. Conc.	RT	Area	ISTD Resp. Ratio	Calc. Conc.	Accuracy	Area	Ratio	RT	Area
Apple Spk 10a	Apple Spk 10a.D	MatrixSpike		2/22/2016 10:42 PM		5.165	1746	0.0368	9.8		1099	63	3.993	47486
Apple Spk 10b	Apple Spk 10b.D	MatrixSpike		2/22/2016 10:57 PM		5.165	1718	0.0404	10.7		914	53	3.993	42561
Apple Spk 10c	Apple Spk 10c.D	MatrixSpike		2/22/2016 11:12 PM		5.165	1758	0.0370	9.8		955	54	3.993	47483
Apple Spk 10d	Apple Spk 10d.D	MatrixSpike		2/22/2016 11:27 PM		5.165	1680	0.0366	9.7		971	58	3.993	45965
Apple Spk 25a	Apple Spk 25a.D	MatrixSpike		2/22/2016 11:42 PM		5.165	4292	0.1066	28.4		2177	51	3.993	40267
Apple Spk 25b	Apple Spk 25b.D	MatrixSpike		2/22/2016 11:57 PM		5.165	4442	0.0993	26.4		2449	55	3.993	44743
Apple Spk 25c	Apple Spk 25c.D	MatrixSpike		2/23/2016 12:12 AM		5.165	3868	0.0922	24.5		2123	55	3.993	41965
Apple Spk 25d	Apple Spk 25d.D	MatrixSpike		2/23/2016 12:27 AM		5.165	3898	0.0924	24.6		2240	57	3.993	42185
Apple Spk 50a	Apple Spk 50a.D	MatrixSpike		2/23/2016 12:42 AM		5.165	7673	0.1891	50.4		4382	57	3.993	40579
Apple Spk 50b	Apple Spk 50b.D	MatrixSpike		2/23/2016 12:57 AM		5.165	6649	0.1884	50.2		3651	55	3.993	35298
Apple Spk 50c	Apple Spk 50c.D	MatrixSpike		2/23/2016 1:13 AM		5.165	5720	0.1822	48.5		2998	52	3.993	31390
Apple Spk 50d	Apple Spk 50d.D	MatrixSpike		2/23/2016 1:28 AM		5.165	7980	0.1817	48.4		4699	59	3.993	43910
Apple Spk 100a	Apple Spk 100a.D	MatrixSpike		2/23/2016 1:43 AM		5.165	15958	0.4028	107.6		8777	55	3.993	39616
Apple Spk 100b	Apple Spk 100b.D	MatrixSpike		2/23/2016 1:58 AM		5.165	14911	0.3939	105.1		8056	54	3.993	37857
Apple Spk 100c	Apple Spk 100c.D	MatrixSpike		2/23/2016 2:13 AM		5.165	19566	0.4106	109.6		10548	54	3.993	47654
Apple Spk 100d	Apple Spk 100d.D	MatrixSpike		2/23/2016 2:28 AM		5.165	16667	0.3949	105.4		9183	55	3.993	42208
Apple Std 0	Apple Std 0.D	QC	1	2/22/2016 8:56 PM		5.165	53	0.0014	0.4		72	134	3.993	37987
Apple Std 5	Apple Std 5.D	QC	2	2/22/2016 9:11 PM	5	5.165	663	0.0188	5.0	99	400	60	3.993	35363
Apple Std 10	Apple Std 10.D	QC	3	2/22/2016 9:26 PM	10	5.165	1964	0.0442	11.7	117	1042	53	3.993	44457
Apple Std 25	Apple Std 25.D	QC	4	2/22/2016 9:42 PM	25	5.165	3578	0.0979	26.0	104	1956	55	3.993	36562
Apple Std 50	Apple Std 50.D	QC	5	2/22/2016 9:57 PM	50	5.165	7152	0.1956	52.1	104	3855	54	3.993	36556
Apple Std 100	Apple Std 100.D	QC	8	2/22/2016 10:12 PM	75	5.165	12430	0.3048	81.3	108	7025	57	3.993	40783
Apple Std 150	Apple Std 150.D	QC	7	2/22/2016 10:27 PM	150	5.165	26905	0.6237	166.9	111	14588	54	3.993	43139
Basil Spk 10a	Basil Spk 10a.D	MatrixSpike		2/25/2016 11:07 PM		5.165	1341	0.0320	8.5		967	72	3.993	41909

black circles = stds in solvent

blue triangles = matrix-matched stds

Compound Information

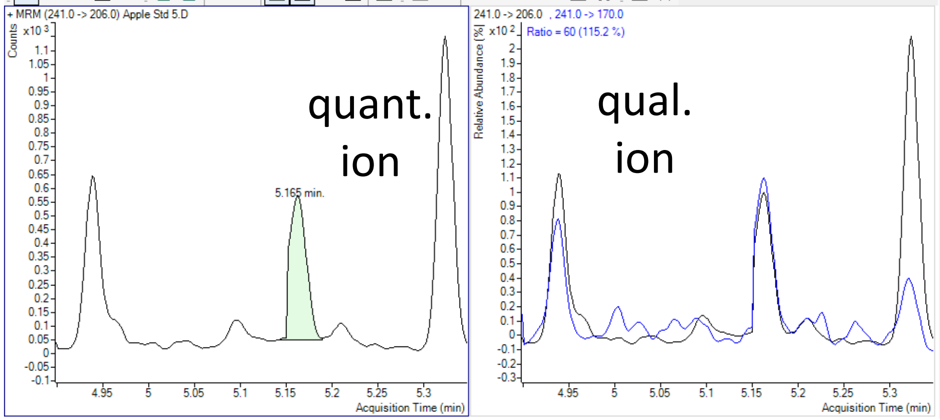

Calibration Curve

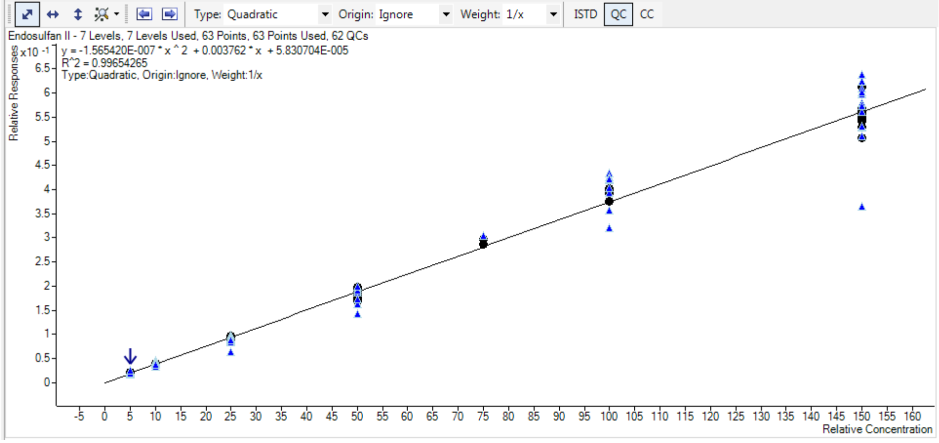

5 ng/mL std in apple

Endosulfan Sulfate Results (vs. atrazine-d5 IS) – all 325 analyses combined!

Sample: Orange Std 10 | Sample Type: <All> | Compound: Endosulfan sulfate | ISTD: Atrazine-d5

Sample							Endosulf...	Endosulfan sulfate Results					Qualifier (387...		Atrazine-d5 (I...	
!	Flag	Name	Data File	Type	Level	Acq. Date-Time	Exp. Conc.	RT	Area	ISTD Resp. Ratio	Calc. Conc.	Accuracy	Area	Ratio	RT	Area
		Olive Std 25	Olive Std 25.D	QC	4	2/24/2016 7:41 PM	25	5.323	961	0.0302	21.5	86	750	78	3.993	31862
		Olive Std 50	Olive Std 50.D	QC	5	2/24/2016 7:55 PM	50	5.323	2454	0.0603	44.1	88	2075	85	3.993	40710
		Olive Std 100	Olive Std 100.D	QC	6	2/24/2016 8:09 PM	100	5.323	3621	0.1404	105.9	106	3193	88	3.993	25791
		Olive Std 150	Olive Std 150.D	QC	7	2/24/2016 8:22 PM	150	5.323	5147	0.2040	156.8	105	4117	80	3.993	25233
		Orange Spk 10a	Orange Spk 10a.D	MatrixSpike		2/24/2016 3:05 PM		5.323	444	0.0164	11.3		388	87	3.993	27066
	⚑	Orange Spk 10b	Orange Spk 10b.D	MatrixSpike		2/24/2016 3:19 PM		5.323	508	0.0183	12.7		345	68	3.993	27740
		Orange Spk 10c	Orange Spk 10c.D	MatrixSpike		2/24/2016 3:32 PM		5.323	463	0.0178	12.4		407	88	3.993	25960
	⚑	Orange Spk 10d	Orange Spk 10d.D	MatrixSpike		2/24/2016 3:46 PM		5.323	321	0.0139	9.5		354	110	3.993	23061
	⚑	Orange Spk 25a	Orange Spk 25a.D	MatrixSpike		2/24/2016 4:00 PM		5.323	1296	0.0413	29.8		1213	94	3.993	31370
		Orange Spk 25b	Orange Spk 25b.D	MatrixSpike		2/24/2016 4:14 PM		5.323	1290	0.0462	33.5		925	72	3.993	27930
		Orange Spk 25c	Orange Spk 25c.D	MatrixSpike		2/24/2016 4:28 PM		5.323	1035	0.0426	30.8		909	88	3.993	24278
		Orange Spk 25d	Orange Spk 25d.D	MatrixSpike		2/24/2016 4:41 PM		5.323	1576	0.0468	33.9		1297	82	3.993	33709
		Orange Spk 50a	Orange Spk 50a.D	MatrixSpike		2/24/2016 4:55 PM		5.323	2544	0.0904	67.0		1970	77	3.993	28141
		Orange Spk 50b	Orange Spk 50b.D	MatrixSpike		2/24/2016 5:09 PM		5.323	2334	0.0873	64.7		1912	82	3.993	26720
		Orange Spk 50c	Orange Spk 50c.D	MatrixSpike		2/24/2016 5:23 PM		5.323	2115	0.0715	52.6		1912	90	3.993	29574
		Orange Spk 50d	Orange Spk 50d.D	MatrixSpike		2/24/2016 5:37 PM		5.323	1945	0.0734	54.1		1625	84	3.993	26489
		Orange Spk 100a	Orange Spk 100a...	MatrixSpike		2/24/2016 5:50 PM		5.323	2816	0.1539	116.5		2324	83	3.993	18297
		Orange Spk 100b	Orange Spk 100b...	MatrixSpike		2/24/2016 6:04 PM		5.323	4022	0.1781	135.8		3329	83	3.993	22584
		Orange Spk 100c	Orange Spk 100c...	MatrixSpike		2/24/2016 6:18 PM		5.323	4272	0.1510	114.2		3673	86	3.993	28295
		Orange Spk 100d	Orange Spk 100d...	MatrixSpike		2/24/2016 6:32 PM		5.323	3701	0.1603	121.6		3134	85	3.993	23087
!	⚑	Orange Std 0	Orange Std 0.D	QC	1	2/24/2016 1:28 PM		5.323	32	0.0011	0.1		37	114	3.993	28356
	⚑	Orange Std 5	Orange Std 5.D	QC	2	2/24/2016 1:42 PM	5	5.323	274	0.0080	5.1	102	152	56	3.993	34357
		Orange Std 10	Orange Std 10.D	QC	3	2/24/2016 1:56 PM	10	5.323	508	0.0160	11.0	110	385	76	3.993	31833
		Orange Std 25	Orange Std 25.D	QC	4	2/24/2016 2:10 PM	25	5.323	1090	0.0305	21.8	87	833	76	3.993	35723

black circles = stds in solvent

blue triangles = matrix-matched stds

Compound Information

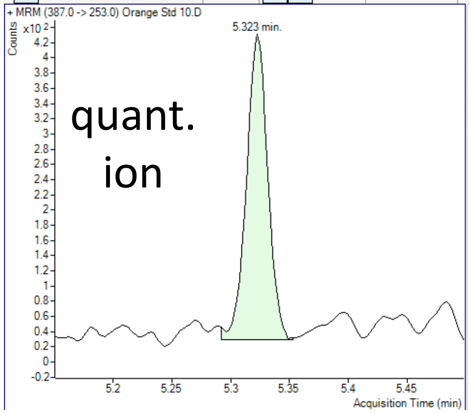

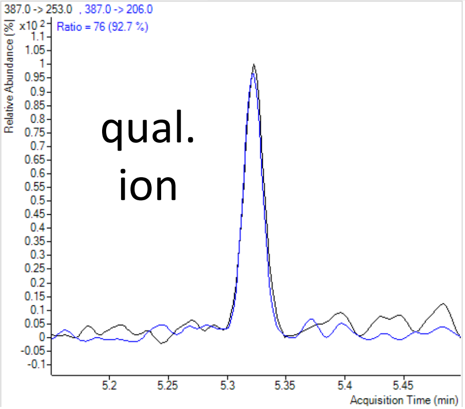

Calibration Curve

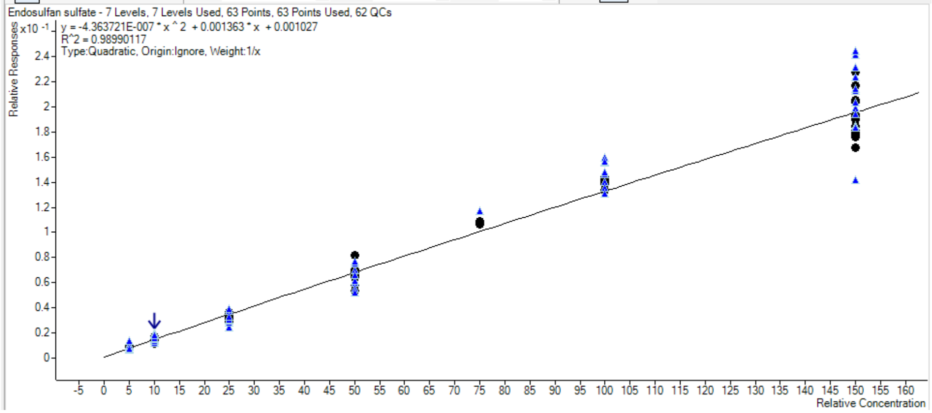

10 ng/mL std in orange

Endosulfan sulfate 5 ng/mL reagent-only and matrix-matched calibration standards

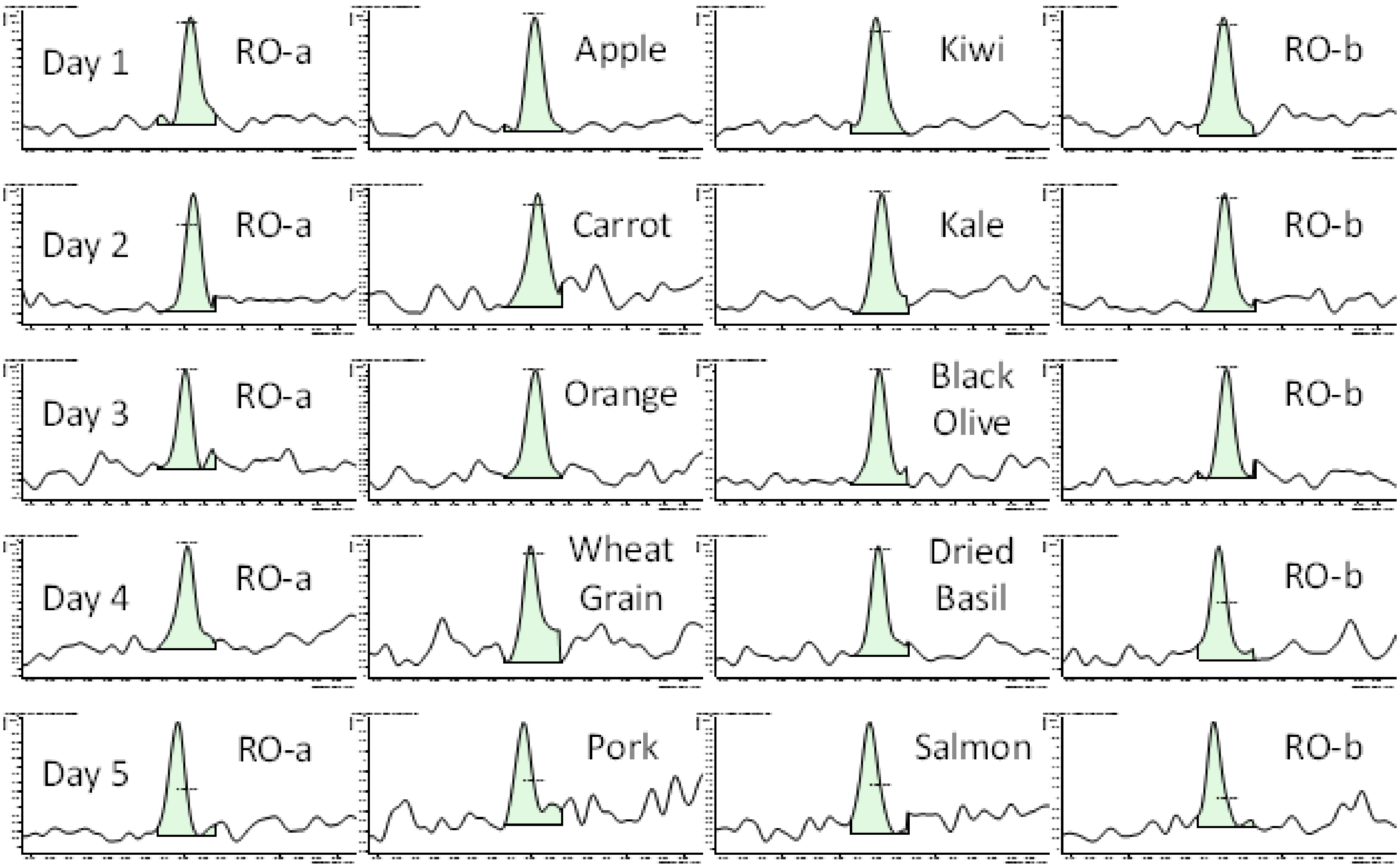

LOQ ≈2 ng/mL in all matrices; even after 325 injections, including 230 food extracts

Bifenthrin Results (vs. atrazine-d5 IS) – all 325 analyses combined!

Sample: Basil Std 5 | Sample Type: <All> | Compound: Bifenthrin | ISTD: Atrazine-d5

Sample					Bifenthrin...	Bifenthrin Results					Qualifier (181...		Atrazine-d5 (I...	
Name	Data File	Type	Level	Acq. Date-Time	Exp. Conc.	RT	Area	ISTD Resp. Ratio	Calc. Conc.	Accuracy	Area	Ratio	RT	Area
Basil Spk 10b	Basil Spk 10b.D	MatrixSpike		2/25/2016 11:21 PM		5.478	199844	3.9821	11.6		134597	67	3.993	50185
Basil Spk 10c	Basil Spk 10c.D	MatrixSpike		2/25/2016 11:35 PM		5.479	208940	4.4649	13.0		142950	68	3.993	46796
Basil Spk 10d	Basil Spk 10d.D	MatrixSpike		2/25/2016 11:49 PM		5.479	190392	4.2995	12.5		130381	68	3.993	44282
Basil Spk 25a	Basil Spk 25a.D	MatrixSpike		2/26/2016 12:03 AM		5.479	258684	8.8700	25.7		181419	70	3.993	29164
Basil Spk 25b	Basil Spk 25b.D	MatrixSpike		2/26/2016 12:16 AM		5.479	314324	8.9155	25.8		218962	70	3.993	35256
Basil Spk 25c	Basil Spk 25c.D	MatrixSpike		2/26/2016 12:30 AM		5.478	238814	9.3222	27.0		166028	70	3.993	25618
Basil Spk 25d	Basil Spk 25d.D	MatrixSpike		2/26/2016 12:44 AM		5.479	279545	10.6452	30.9		195977	70	3.993	26260
Basil Spk 50a	Basil Spk 50a.D	MatrixSpike		2/26/2016 12:58 AM		5.479	727185	14.8792	43.2		506446	70	3.993	48873
Basil Spk 50b	Basil Spk 50b.D	MatrixSpike		2/26/2016 1:12 AM		5.479	507358	20.1732	58.9		354625	70	3.993	25150
Basil Spk 50c	Basil Spk 50c.D	MatrixSpike		2/26/2016 1:26 AM		5.479	521687	18.2518	53.2		363985	70	3.993	28583
Basil Spk 50d	Basil Spk 50d.D	MatrixSpike		2/26/2016 1:39 AM		5.478	565931	19.0320	55.5		400053	71	3.993	29736
Basil Spk 100a	Basil Spk 100a.D	MatrixSpike		2/26/2016 1:53 AM		5.479	12651...	37.0898	109.9		876472	69	3.993	34111
Basil Spk 100b	Basil Spk 100b.D	MatrixSpike		2/26/2016 2:07 AM		5.479	12053...	37.6845	111.7		845838	70	3.993	31984
Basil Spk 100c	Basil Spk 100c.D	MatrixSpike		2/26/2016 2:21 AM		5.479	21562...	38.3068	113.6		15078...	70	3.993	56290
Basil Spk 100d	Basil Spk 100d.D	MatrixSpike		2/26/2016 2:35 AM		5.479	13750...	42.2305	125.7		958857	70	3.993	32561
Basil Std 0	Basil Std 0.D	QC	1	2/25/2016 9:31 PM		5.479	13123	0.3763	1.2		10274	78	3.993	34874
Basil Std 5	Basil Std 5.D	QC	2	2/25/2016 9:44 PM	5	5.479	125170	2.4825	7.3	145	81864	65	3.993	50421
Basil Std 10	Basil Std 10.D	QC	3	2/25/2016 9:58 PM	10	5.479	115471	4.7655	13.8	138	79589	69	3.993	24231
Basil Std 25	Basil Std 25.D	QC	4	2/25/2016 10:12 PM	25	5.478	287413	9.8276	28.5	114	200906	70	3.993	29245
Basil Std 50	Basil Std 50.D	QC	5	2/25/2016 10:26 PM	50	5.479	893327	18.7563	54.7	109	625677	70	3.993	47628
Basil Std 100	Basil Std 100.D	QC	6	2/25/2016 10:40 PM	100	5.479	13448...	44.2361	132.0	132	946920	70	3.993	30401
Basil Std 150	Basil Std 150.D	QC	7	2/25/2016 10:54 PM	150	5.479	18756...	47.7173	142.9	95	13103...	70	3.993	39308
Carrot Spk 10a	Carrot Spk 10a.D	MatrixSpike		2/23/2016 9:50 PM		5.479	133104	3.8268	11.1		91902	69	3.993	34782
Carrot Spk 10b	Carrot Spk 10b.D	MatrixSpike		2/23/2016 10:03 PM		5.478	156939	5.1699	15.0		107993	69	3.993	30356

black circles = stds in solvent

blue triangles = matrix-matched stds

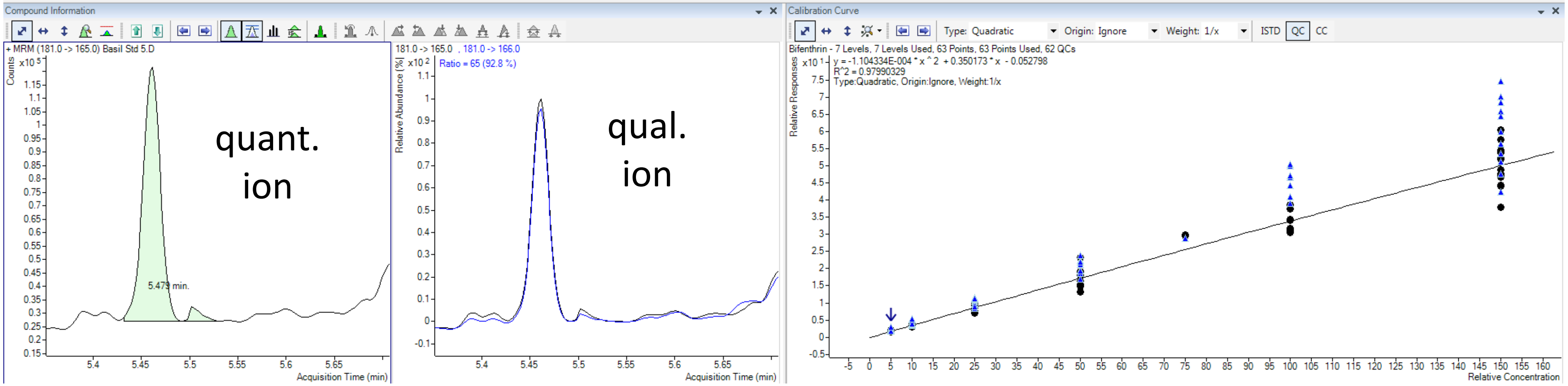

5 ng/mL std in basil

Benzo(b+k)fluoranthene (vs. benzo(a)pyrene-d12 IS) – all 325 analyses combined!

Sample: Wheat Std 5 | Sample Type: <All> | Compound: Benzo(b+k)fluoranthene | ISTD: Benzo(a)pyrene-d12

Sample					Benzo(b+k)fluorant...	Benzo(b+k)fluoranthene Results					Qualifier (252.0 -> 226.0) Res...		Benzo(a)pyrene-d12 (ISTD)...	
Name	Data File	Type	Level	Acq. Date-Time	Exp. Conc.	RT	Area	Calc. Conc.	Accuracy	S/N	Area	Ratio	RT	Area
SO Std 100a WB	SO Std 100a WB....	Cal	6	2/25/2016 3:31 PM	100.0	6.064	1936972	112.6	112.6	Infinity	279131	14.4	6.231	624903
SO Std 150a WB	SO Std 150a WB....	Cal	7	2/25/2016 3:45 PM	150.0	6.064	3035832	160.8	107.2	Infinity	437294	14.4	6.231	693060
Wheat Std 0	Wheat Std 0.D	Cal	1	2/25/2016 3:59 PM	0.0								6.231	693060
Wheat Std 5	Wheat Std 5.D	Cal	2	2/25/2016 4:13 PM	5.0	6.064	82796	6.3	125.2	Infinity	12084	14.6	6.231	565034
Wheat Std 10	Wheat Std 10.D	Cal	3	2/25/2016 4:27 PM	10.0	6.064	293664	10.8	108.1	Infinity	42073	14.3	6.231	1061756
Wheat Std 25	Wheat Std 25.D	Cal	4	2/25/2016 4:40 PM	25.0	6.064	418094	24.8	99.3	Infinity	60457	14.5	6.231	619127
Wheat Std 50	Wheat Std 50.D	Cal	5	2/25/2016 4:54 PM	50.0	6.064	1564800	47.6	95.1	Infinity	227514	14.5	6.231	1190053
Wheat Std 100	Wheat Std 100.D	Cal	6	2/25/2016 5:08 PM	100.0	6.064	2959694	104.3	104.3	Infinity	428522	14.5	6.231	1028953
Wheat Std 150	Wheat Std 150.D	Cal	7	2/25/2016 5:22 PM	150.0	6.064	4508028	138.5	92.3	Infinity	649652	14.4	6.231	1188690
Wheat Spk 10a	Wheat Spk 10a.D	MatrixSpike		2/25/2016 5:36 PM		6.064	47831	13.8		Infinity			6.231	132607
Wheat Spk 10b	Wheat Spk 10b.D	MatrixSpike		2/25/2016 5:50 PM		6.064	55259	13.1		Infinity			6.231	161655
Wheat Spk 10c	Wheat Spk 10c.D	MatrixSpike		2/25/2016 6:03 PM		6.064	70466	12.8		Infinity	10507	14.9	6.231	211725
Wheat Spk 10d	Wheat Spk 10d.D	MatrixSpike		2/25/2016 6:17 PM		6.064	49995	13.0		Infinity			6.231	147366
Wheat Spk 25a	Wheat Spk 25a.D	MatrixSpike		2/25/2016 6:31 PM		6.064	121877	29.2		Infinity	17673	14.5	6.231	152481
Wheat Spk 25b	Wheat Spk 25b.D	MatrixSpike		2/25/2016 6:45 PM		6.064	169398	28.5		Infinity	24432	14.4	6.231	217599
Wheat Spk 25c	Wheat Spk 25c.D	MatrixSpike		2/25/2016 6:59 PM		6.064	114582	28.1		Infinity	16959	14.8	6.231	149044
Wheat Spk 25d	Wheat Spk 25d.D	MatrixSpike		2/25/2016 7:12 PM		6.064	93860	28.8		Infinity	13579	14.5	6.231	119124
Wheat Spk 50a	Wheat Spk 50a.D	MatrixSpike		2/25/2016 7:26 PM		6.064	336425	55.7		Infinity	48392	14.4	6.231	218013
Wheat Spk 50b	Wheat Spk 50b.D	MatrixSpike		2/25/2016 7:40 PM		6.064	180299	57.3		Infinity	26029	14.4	6.231	113565
Wheat Spk 50c	Wheat Spk 50c.D	MatrixSpike		2/25/2016 7:54 PM		6.064	231220	55.9		Infinity	34338	14.9	6.231	149433
Wheat Spk 50d	Wheat Spk 50d.D	MatrixSpike		2/25/2016 8:08 PM		6.064	233703	55.9		Infinity	33808	14.5	6.231	150866
Wheat Spk 100a	Wheat Spk 100a.D	MatrixSpike		2/25/2016 8:22 PM		6.064	825055	121.8		Infinity	119013	14.4	6.231	246358
Wheat Spk 100b	Wheat Spk 100b.D	MatrixSpike		2/25/2016 8:35 PM		6.064	481118	124.3		Infinity	69170	14.4	6.231	140834
Wheat Spk 100c	Wheat Spk 100c.D	MatrixSpike		2/25/2016 8:49 PM		6.064	781689	121.1		Infinity	112560	14.4	6.231	234811

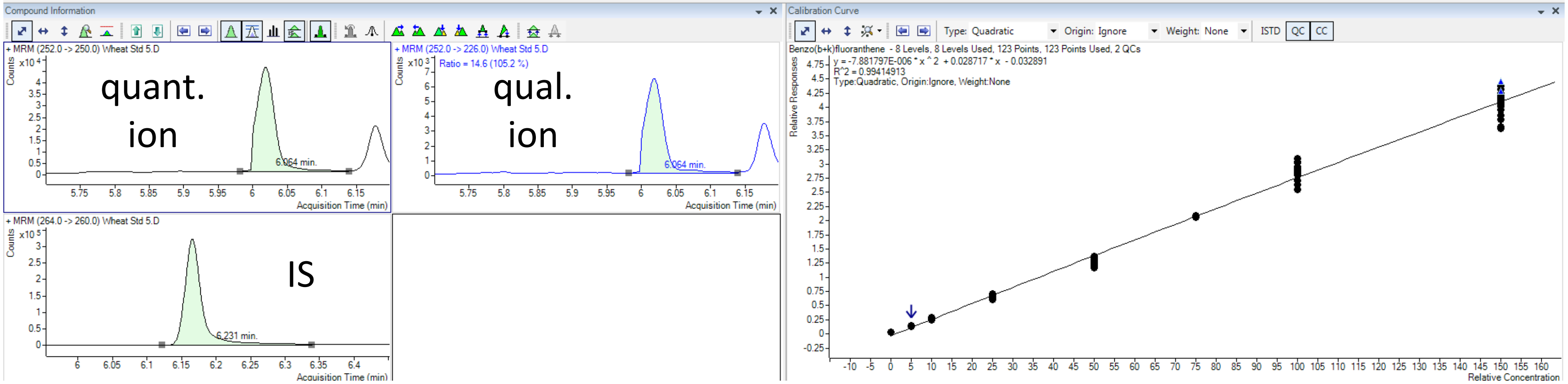

5 ng/mL std in wheat grain

(Es)fenvalerate Results (vs. atrazine-d5 IS) – all 325 analyses combined!

Sample: Orange Std 10 | Sample Type: <All> | Compound: Esfenvalerate | ISTD: Atrazine-d5

Name	Data File	Type	Level	Acq. Date-Time	Esfe... Exp. Conc.	Esfenvalerate Results RT	Area	ISTD Resp. Ratio	Calc. Conc.	Accuracy	Qualifier (167.0 ->... Area	Ratio	Atrazine-d5 (ISTD) R... RT	Area
Olive Std 100	Olive Std 100.D	QC	6	2/24/2016 8:09 PM	100	6.259	248420	9.6322	191.1	191	72009	29	3.993	25791
Olive Std 150	Olive Std 150.D	QC	7	2/24/2016 8:22 PM	150	6.259	349170	13.8378	286.1	191	100387	29	3.993	25233
Orange Spk 10a	Orange Spk 10a.D	MatrixSpike		2/24/2016 3:05 PM		6.259	22523	0.8321	16.0		5875	26	3.993	27066
Orange Spk 10b	Orange Spk 10b.D	MatrixSpike		2/24/2016 3:19 PM		6.259	23044	0.8307	15.9		6380	28	3.993	27740
Orange Spk 10c	Orange Spk 10c.D	MatrixSpike		2/24/2016 3:32 PM		6.259	23669	0.9118	17.4		6340	27	3.993	25960
Orange Spk 10d	Orange Spk 10d.D	MatrixSpike		2/24/2016 3:46 PM		6.259	19765	0.8571	16.4		5882	30	3.993	23061
Orange Spk 25a	Orange Spk 25a.D	MatrixSpike		2/24/2016 4:00 PM		6.259	55591	1.7721	33.5		16341	29	3.993	31370
Orange Spk 25b	Orange Spk 25b.D	MatrixSpike		2/24/2016 4:14 PM		6.259	56016	2.0056	37.8		16096	29	3.993	27930
Orange Spk 25c	Orange Spk 25c.D	MatrixSpike		2/24/2016 4:28 PM		6.259	48337	1.9910	37.6		14201	29	3.993	24278
Orange Spk 25d	Orange Spk 25d.D	MatrixSpike		2/24/2016 4:41 PM		6.259	61739	1.8315	34.6		18103	29	3.993	33709
Orange Spk 50a	Orange Spk 50a.D	MatrixSpike		2/24/2016 4:55 PM		6.259	109309	3.8843	73.7		31245	29	3.993	28141
Orange Spk 50b	Orange Spk 50b.D	MatrixSpike		2/24/2016 5:09 PM		6.259	99846	3.7368	70.8		28779	29	3.993	26720
Orange Spk 50c	Orange Spk 50c.D	MatrixSpike		2/24/2016 5:23 PM		6.259	110998	3.7533	71.2		31963	29	3.993	29574
Orange Spk 50d	Orange Spk 50d.D	MatrixSpike		2/24/2016 5:37 PM		6.259	97928	3.6970	70.1		29405	30	3.993	26489
Orange Spk 100a	Orange Spk 100a...	MatrixSpike		2/24/2016 5:50 PM		6.259	141594	7.7385	151.0		40646	29	3.993	18297
Orange Spk 100b	Orange Spk 100b...	MatrixSpike		2/24/2016 6:04 PM		6.259	171594	7.5981	148.1		49731	29	3.993	22584
Orange Spk 100c	Orange Spk 100c...	MatrixSpike		2/24/2016 6:18 PM		6.259	193961	6.8550	132.8		56368	29	3.993	28295
Orange Spk 100d	Orange Spk 100d...	MatrixSpike		2/24/2016 6:32 PM		6.259	176785	7.6574	149.3		51517	29	3.993	23087
Orange Std 0	Orange Std 0.D	QC	1	2/24/2016 1:28 PM		6.259	549	0.0194	1.1		1041	190	3.993	28356
Orange Std 5	Orange Std 5.D	QC	2	2/24/2016 1:42 PM	5	6.259	13151	0.3828	7.7	154	3179	24	3.993	34357
Orange Std 10	Orange Std 10.D	QC	3	2/24/2016 1:56 PM	10	6.259	24199	0.7602	14.6	146	7132	29	3.993	31833
Orange Std 25	Orange Std 25.D	QC	4	2/24/2016 2:10 PM	25	6.259	55971	1.5668	29.6	118	16233	29	3.993	35723
Orange Std 50	Orange Std 50.D	QC	5	2/24/2016 2:23 PM	50	6.259	70276	3.1018	58.6	117	19968	28	3.993	22657

black circles = stds in solvent

blue triangles = matrix-matched stds

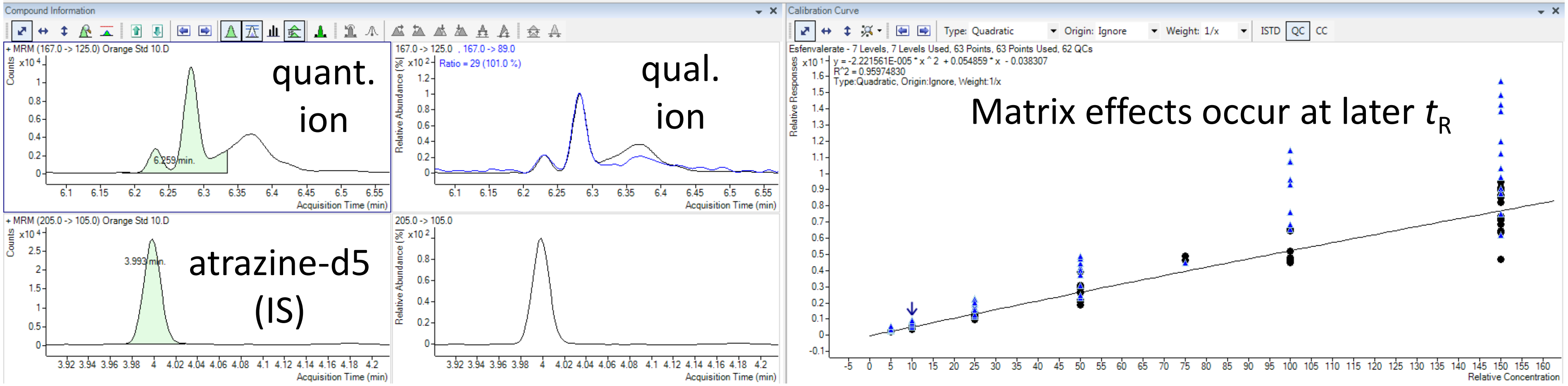

10 ng/mL std in orange

a ghost peak occurs in orange 1 injection later

Dechlorane Plus Results (vs. FBDE 126 IS) – all 325 analyses combined!

Sample: Wheat Std 5 | Sample Type: <All> | Compound: Dechlorane plus | ISTD: FBDE 126

Name	Data File	Type	Level	Acq. Date-Time	Dechlorane plus M... Exp. Conc.	Dechlorane plus Results RT	Area	Calc. Conc.	Accuracy	S/N	Qualifier (272.0 -> 235.0) Res... Area	Ratio	FBDE 126 (ISTD) Results RT	Area
SO Std 100a WB	SO Std 100a WB....	Cal	6	2/25/2016 3:31 PM	100.0	8.300	41527	115.6	115.6	Infinity	8225	19.8	6.121	9082
SO Std 150a WB	SO Std 150a WB....	Cal	7	2/25/2016 3:45 PM	150.0	8.300	58046	153.6	102.4	Infinity	11728	20.2	6.121	9783
Wheat Std 0	Wheat Std 0.D	Cal	1	2/25/2016 3:59 PM	0.0								6.121	8343
Wheat Std 5	Wheat Std 5.D	Cal	2	2/25/2016 4:13 PM	5.0	8.300	2063	6.8	135.9	Infinity			6.121	8520
Wheat Std 10	Wheat Std 10.D	Cal	3	2/25/2016 4:27 PM	10.0	8.300	7756	11.4	114.2	Infinity	1608	20.7	6.121	17664
Wheat Std 25	Wheat Std 25.D	Cal	4	2/25/2016 4:40 PM	25.0	8.300	10484	27.2	108.6	Infinity	2181	20.8	6.121	9523
Wheat Std 50	Wheat Std 50.D	Cal	5	2/25/2016 4:54 PM	50.0	8.300	42590	49.7	99.3	Infinity	8531	20.0	6.121	21040
Wheat Std 100	Wheat Std 100.D	Cal	6	2/25/2016 5:08 PM	100.0	8.300	78508	115.0	115.0	Infinity	15726	20.0	6.121	17250
Wheat Std 150	Wheat Std 150.D	Cal	7	2/25/2016 5:22 PM	150.0	8.300	130511	159.7	106.5	Infinity	26530	20.3	6.121	21235
Wheat Spk 10a	Wheat Spk 10a.D	MatrixSpike		2/25/2016 5:36 PM		8.300	3735	12.1		Infinity			6.121	7964
Wheat Spk 10b	Wheat Spk 10b.D	MatrixSpike		2/25/2016 5:50 PM		8.300	4466	11.3		Infinity	1017	22.8	6.121	10259
Wheat Spk 10c	Wheat Spk 10c.D	MatrixSpike		2/25/2016 6:03 PM		8.300	5940	11.1		Infinity	1314	22.1	6.121	13912
Wheat Spk 10d	Wheat Spk 10d.D	MatrixSpike		2/25/2016 6:17 PM		8.300	4331	11.5		Infinity	1003	23.2	6.121	9809
Wheat Spk 25a	Wheat Spk 25a.D	MatrixSpike		2/25/2016 6:31 PM		8.300	9536	22.4		Infinity	1996	20.9	6.121	10590
Wheat Spk 25b	Wheat Spk 25b.D	MatrixSpike		2/25/2016 6:45 PM		8.300	13918	25.6		Infinity	2908	20.9	6.121	13440
Wheat Spk 25c	Wheat Spk 25c.D	MatrixSpike		2/25/2016 6:59 PM		8.300	8867	27.1		Infinity	1820	20.5	6.121	8085
Wheat Spk 25d	Wheat Spk 25d.D	MatrixSpike		2/25/2016 7:12 PM		8.300	7767	25.3		Infinity	1640	21.1	6.121	7588
Wheat Spk 50a	Wheat Spk 50a.D	MatrixSpike		2/25/2016 7:26 PM		8.300	31155	51.2		Infinity	6322	20.3	6.121	14926
Wheat Spk 50b	Wheat Spk 50b.D	MatrixSpike		2/25/2016 7:40 PM		8.300	14057	51.7		Infinity	2992	21.3	6.121	6669
Wheat Spk 50c	Wheat Spk 50c.D	MatrixSpike		2/25/2016 7:54 PM		8.300	17368	48.3		Infinity	3538	20.4	6.121	8827
Wheat Spk 50d	Wheat Spk 50d.D	MatrixSpike		2/25/2016 8:08 PM		8.300	18843	48.9		Infinity	3874	20.6	6.121	9457
Wheat Spk 100a	Wheat Spk 100a.D	MatrixSpike		2/25/2016 8:22 PM		8.300	64986	101.8		Infinity	13133	20.2	6.121	16006
Wheat Spk 100b	Wheat Spk 100b.D	MatrixSpike		2/25/2016 8:35 PM		8.300	35795	103.5		Infinity	7285	20.4	6.121	8680
Wheat Spk 100c	Wheat Spk 100c.D	MatrixSpike		2/25/2016 8:49 PM		8.300	54251	105.0		Infinity	11049	20.4	6.121	12985

black circles = stds in solvent

blue triangles = matrix-matched stds

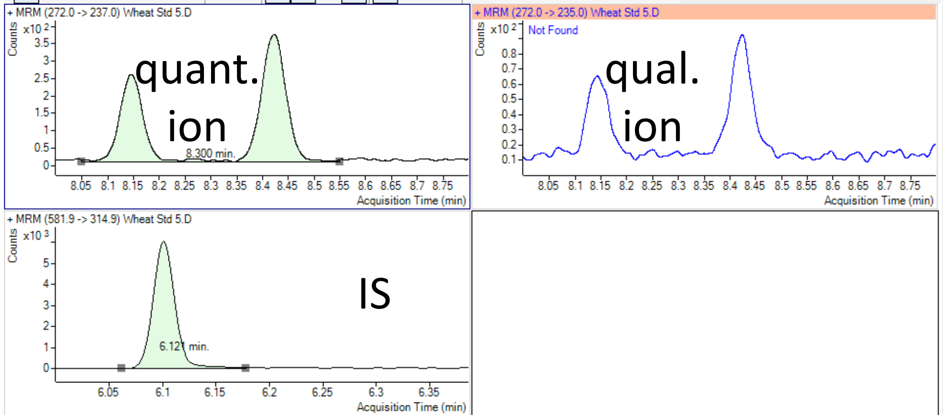

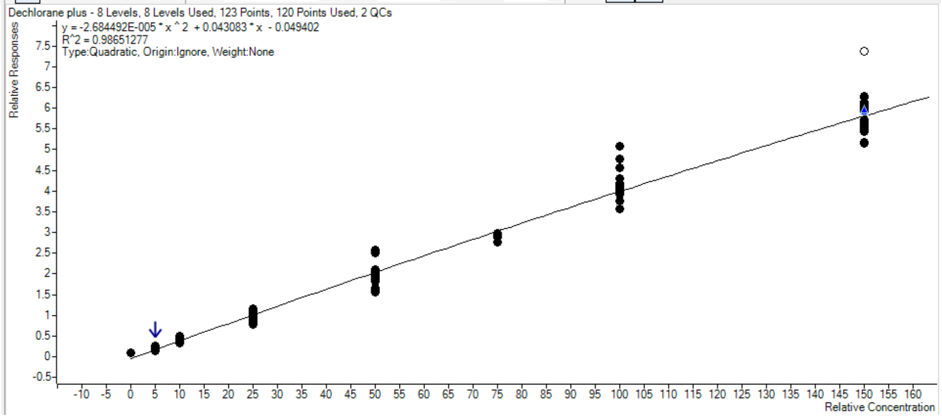

5 ng/mL std in wheat grain

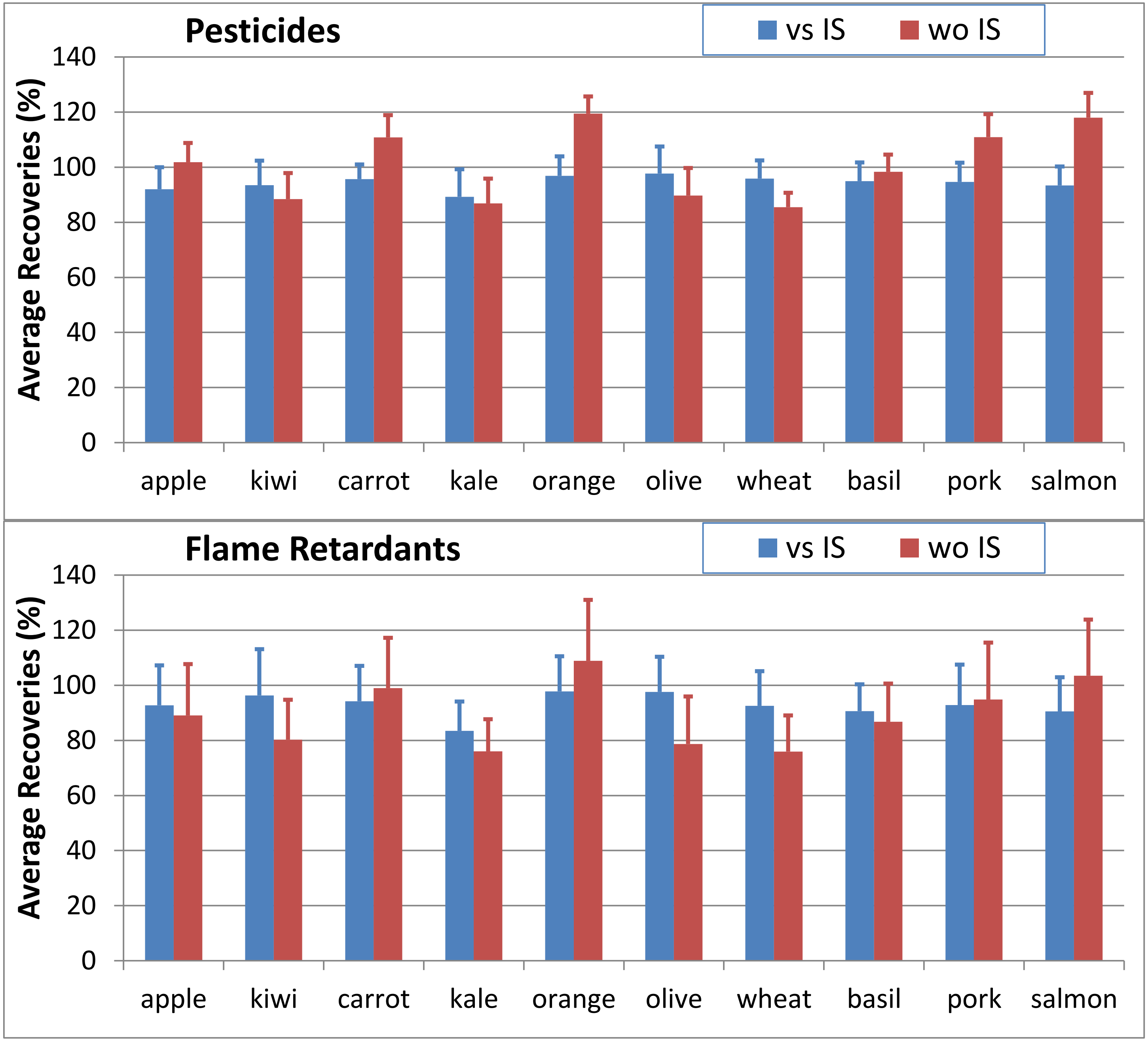
Pesticides
vs IS
wo IS
Average Recoveries (%)
140
120
100
80
60
40
20
0
apple
kiwi
carrot
kale
orange
olive
wheat
basil
pork
salmon
Flame Retardants
vs IS
wo IS
Average Recoveries (%)
140
120
100
80
60
40
20
0
apple
kiwi
carrot
kale
orange
olive
wheat
basil
pork
salmon

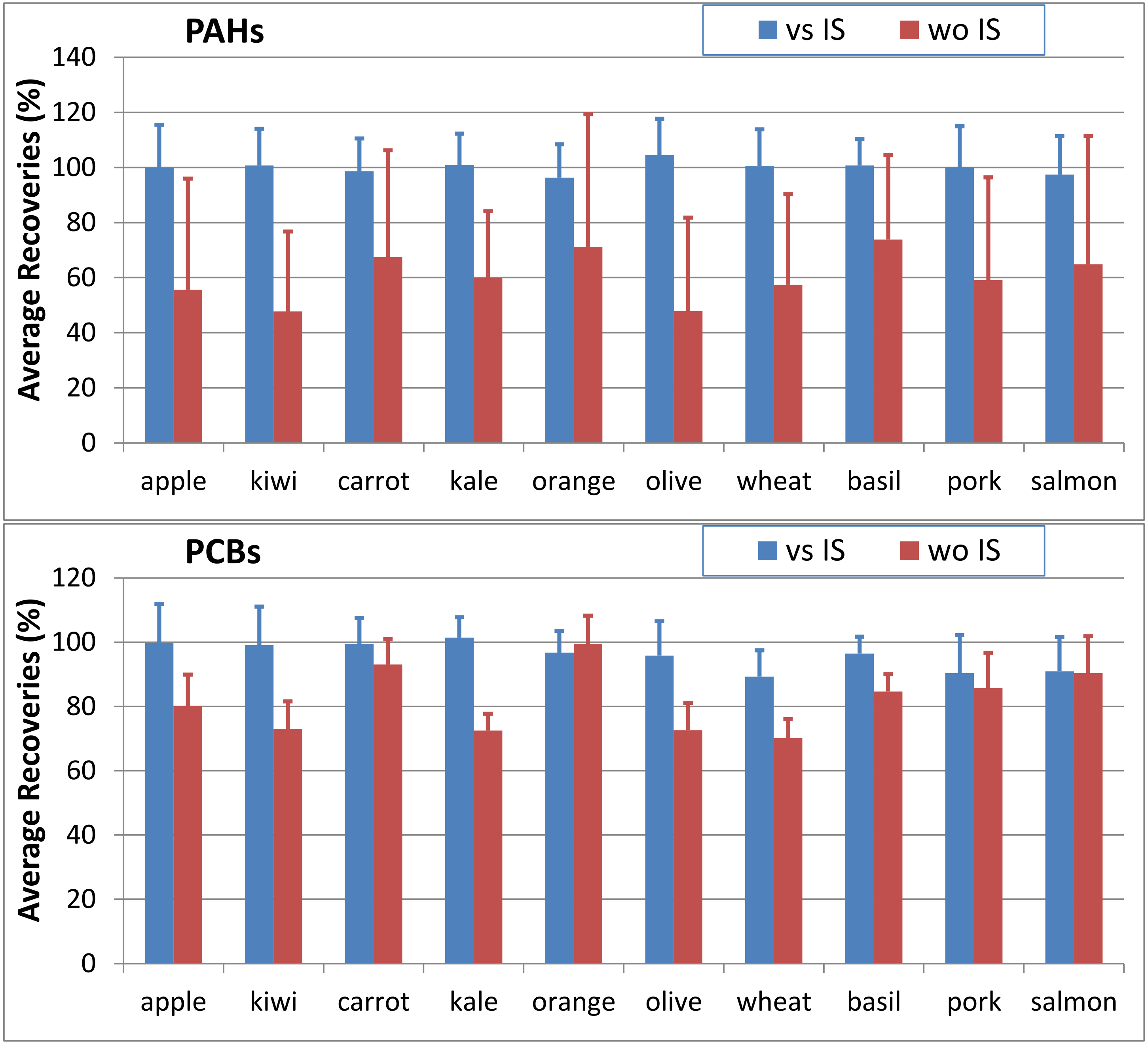
PAHs
vs IS
wo IS
Average Recoveries (%)
0
20
40
60
80
100
120
140
apple
kiwi
carrot
kale
orange
olive
wheat
basil
pork
salmon
PCBs
vs IS
wo IS
Average Recoveries (%)
0
20
40
60
80
100
120
apple
kiwi
carrot
kale
orange
olive
wheat
basil
pork
salmon

Numbers correspond to analytes listed in Table 1

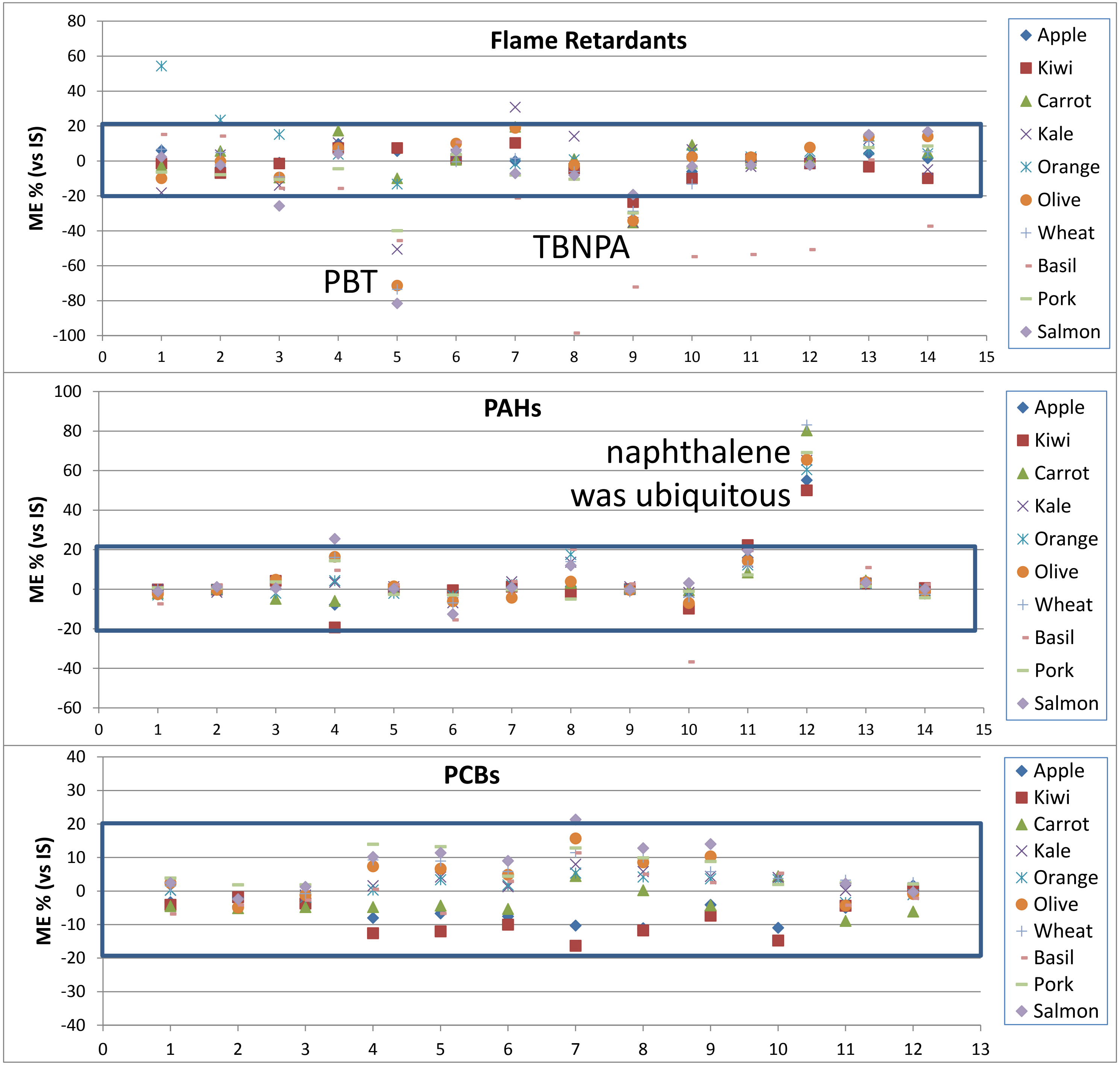

Supplement: Supplementary file 1 — Supplementary material 1 (PDF 4212 kb) [file 10337_2016_3116_MOESM1_ESM.pdf]
